# Nutritional restriction during the peri-conceptional period alters the myometrial transcriptome during the peri-implantation period

**DOI:** 10.1038/s41598-021-00533-x

**Published:** 2021-10-27

**Authors:** Ewa Monika Drzewiecka, Wiktoria Kozlowska, Agata Zmijewska, Anita Franczak

**Affiliations:** Department of Animal Anatomy and Physiology, Faculty of Biology and Biotechnology, University of Warmia and Mazury in Olsztyn, Oczapowskiego 1A, 10-719 Olsztyn, Poland

**Keywords:** Risk factors, Transcriptomics, Animal physiology

## Abstract

This study hypothesized that female peri-conceptional undernutrition evokes transcriptomic alterations in the pig myometrium during the peri-implantation period. Myometrium was collected on days 15–16 of pregnancy from pigs fed a normal- (n = 4) or restricted-diet (n = 4) from conception until day 9th of pregnancy, and the transcriptomic profiles of the tissue were compared using Porcine (V2) Expression Microarrays 4 × 44 K. In restricted diet-fed pigs, 1021 differentially expressed genes (DEGs) with fold change ≥ 1.5, *P* ≤ 0.05 were revealed, and 708 of them were up-regulated. Based on the count score, the top within GOs was GO cellular components “*extracellular exosome*”, and the top KEGG pathway was the metabolic pathway. Ten selected DEGs, i.e.* hydroxysteroid (17β) dehydrogenase 8*, *cyclooxygenase 2, prostaglandin F receptor*, *progesterone receptor membrane component 1*, *progesterone receptor membrane component 2*, *annexin A2*, *homeobox A10*, *S-phase cyclin A-associated protein in the ER*, *SRC proto-oncogene*, *non-receptor tyrosine kinase*, and *proliferating cell nuclear antigen* were conducted through qPCR to validate microarray data. In conclusion, dietary restriction during the peri-conceptional period causes alterations in the expression of genes encoding proteins involved i.a. in the endocrine activity of the myometrium, embryo-maternal interactions, and mechanisms regulating cell cycle and proliferation.

## Introduction

The proper nutrition of a female during the peri-conceptional period impacts the reproductive success, pregnancy outcome, and health of future offspring^1–9^. Females fed an unbalanced diet exhibit endocrine disruptions, the decreased rate of ovulation, the deteriorated oocytes quality, and the lowered potential of embryos to develop^1–3,7–12^. Poor nutrition in pregnant females is one of the risk factors of high priority that may evoke fetal growth retardation^13,14^. Moreover, an unbalanced diet during pregnancy can cause low birth weight, and excessive growth, obesity, circulatory system disorders, and hypertension in adulthood^1–6,12^. However, the origin and the molecular background of these effects still appear not fully understood.

According to the Developmental Origins of Health and Disease concept, the changes in embryos and offspring starting at the early stages of development may depend on the intrauterine milieu^1,2,4,13,15,16^. It is believed that alterations in this milieu may be especially hazardous while appearing during the peri-conceptional period^1,4,15,17–20^. The composition of the intrauterine environment depends on i.a. maternal diet^16^, but also secretory activity of uterine tissues, including the myometrium^21–26^. Studies performed on pigs demonstrated that the peri-conceptional undernutrition leads to the decrease of the total content of estradiol-17*β* (E_2_) in the uterine flushings during the peri-implantation period^17^. This effect can be a result of the altered steroidogenic activity of the uterus. Furthermore, the previous studies have shown that the peri-conceptional undernutrition induces global transcriptomic changes in conceptuses and the endometrium of females during the peri-implantation period^18,27^ suggesting that dietary restriction may affect a variety of mechanisms occurring in the uterus for proper embryo implantation and future development. Until now the effect of dietary restriction on myometrial transcriptome was not studied. However, there was documented an effect of restricted diet on the myometrial expression of methyltransferases, determining the potential for DNA methylation^17^. Therefore, it cannot be excluded that changes in the myometrial potential for DNA methylation, as an effect of an unbalanced diet, may induce transcriptomic alterations in the tissue, determining its potential and activity.

This study hypothesized that undernutrition in females during the peri-conceptional period evokes alterations in the transcriptomic profile of the myometrium during the peri-implantation period. Thus, the current study aimed to determine and compare the transcriptomic profiles of the peri-implantation myometrium collected from females fed a normal- or restricted-diet during the peri-conceptional period. The study was performed on pig model, however, it is intended to imitate deprivation of nutritional habits in women who, during early stages of pregnancy, i.e. during the peri-conceptional period, are not aware of carrying a child. In this study, a particular focus was put on differentially expressed genes (DEGs) that are known to encode proteins crucial for the creation of the proper intrauterine environment, embryo-maternal interactions, and myometrial morphogenesis. Furthermore, there was performed transcription factor enrichment analysis to provide insight into the mechanisms of alterations in the transcriptomic activity of the myometrium in response to dietary restriction. Finally, there were evaluated common DEGs in the myometrium, endometrium^18^, conceptuses^27^ of pigs fed a restricted diet during the periconceptional period. This study provides new data concerning molecular alterations in the uterine tissues and conceptuses during the peri-implantation period that occur as the consequence of female peri-conceptional undernutrition.

## Results

### Differentially expressed genes (DEGs)

The transcriptomic analysis evaluated 1021 DEGs (FC ≥ 1.5; *P* ≤ 0.05) in the myometrium collected during the peri-implantation period from restricted-diet-fed pigs compared to the myometrium collected from normal-diet-fed pigs. Among evaluated DEGs, 69.34% (708 DEGs) were up-regulated and 30.66% (313 DEGs) were down-regulated. The full list of DEGs is presented in Supplementary Table 1, and the top 15 up- and top 15 down-regulated genes are listed in Table 1. Considering the function of the proteins encoded by listed DEGs, based on GeneCards: The Human Gene Database (https://www.genecards.org/), three groups of DEGs were evaluated. The first grouped genes which products are involved in the creation of a proper intrauterine environment, i.e.: synthesis of cholesterol, steroid hormones, and prostaglandins in the myometrium, and myometrial response to their action (Group 1: The endocrine activity), second grouped genes which products are involved in the regulation of embryo-maternal interactions during the peri-implantation period and involved in processes associated with the implantation (Group 2: Embryo-maternal interactions), and third one grouped genes involved in the regulation of myometrial cell proliferation and differentiation, and tissue morphogenesis (Group 3: Mechanisms of proliferation and differentiation). The selected genes within Groups 1, 2, and 3 are presented in Table 2.

**Table 1:** The top 15 up-, and down-regulated differentially expressed genes (DEGs) in the myometrium of pigs during the peri-implantation period in response to dietary restriction during the peri-conceptional period.

*P*-value	FC (abs)	Official gene symbol	Accession No. to reference sequence in GenBank	Direction of regulation
0.01653065	5.71	*NMB*	NM_001123145	Up
0.04747134	4.75	*RBP4*	XM_021072103	Up
0.01679052	4.60	*UFBP-2*	NM_213845	Up
0.04731457	4.31	*WNT2*	XM_003134756	Up
0.01802151	4.14	*HTR1B*	XM_005659424	Up
0.02969932	4.07	*UABP-2*	NM_213845	Up
0.04464868	3.87	*ACTA1*	XM_005670976	Up
0.02330657	3.56	*SAA3*	XM_013994503	Up
0.02747832	3.16	*FXYD3*	NM_214208	Up
0.00449847	3.07	*NPY*	NM_001256367	Up
0.01936780	3.04	*EPC1*	AK348170	Up
0.04365449	3.02	*SLC16A1*	NM_001128445	Up
0.02760882	2.95	*SRPX*	XM_001927105	Up
0.04386596	2.90	*GPX3*	NM_001115155	Up
0.04180541	2.84	*SCNN1A*	XM_005653156	Up
0.00848236	4.90	*PELO*	XM_013984761	Down
0.01282775	4.80	*FOXP2*	XM_021078435	Down
0.00351351	4.55	*SLC4A7*	XM_021071476	Down
0.02605828	4.50	*IRF2BP2*	XM_021074288	Down
0.00014711	4.31	*FZD1*	XM_021102361	Down
0.00164190	4.24	*PHACTR2*	XM_021087164	Down
0.00472723	3.78	*LOC100621006*	XM_021101377	Down
0.01119047	3.62	*LOC106509575*	XR_001307225.2	Down
0.00337159	3.59	*HNRNPLL*	XM_019942803	Down
0.02503123	3.57	*REST*	XM_005666690	Down
0.01500155	3.56	*HOOK3*	XM_021077850	Down
0.00461170	3.52	*DDX39B*	XR_002345591	Down
0.01241197	3.49	*MEF2C*	XM_021082279	Down
0.02681823	3.49	*ZFHX4*	XM_021089177	Down
0.00503120	3.34	*ZBTB44*	XM_003130079	Down

**Table 2:** The selected differentially expressed genes (DEGs) in response to peri-conceptional undernutrition in the myometrium of pigs during the peri-implantation period, classified based on the function of encoded products.

Regulation	Gene symbol	Description	Fold change
**Group 1: The endocrine activity**			
Up	*PGRMC1*	*Progesterone receptor membrane component 1*	2.43
	*HMGCR*	*3-Hydroxy-3-methylglutaryl-CoA reductase*	2.32
	*CYP39A1*	*Cytochrome P450 family 39 subfamily A polypeptide 1*	2.22
	*HSD17B8*	*Hydroxysteroid (17β) dehydrogenase 8*	1.91
	*NSDHL*	*NAD(P) dependent steroid dehydrogenase-like*	1.69
	*HSD11B1*	*Hydroxysteroid (11β) dehydrogenase 1*	1.55
Down	*PTGFR*	*Prostaglandin F receptor*	2.01
	*COX2*	*Cyclooxygenase 2*	1.78
	*PGRMC2*	*Progesterone receptor membrane component 2*	1.54
**Group 2: Embryo-maternal interactions**			
Up	*TPBG*	*Trophoblast glycoprotein*	2.10
	*PGRMC1*	*Progesterone receptor membrane component 1*	2.43
	*PECAM1*	*Platelet/endothelial cell adhesion molecule 1*	1.57
	*ICAM3*	*Intercellular adhesion molecule 3*	1.94
	*ICAM1*	*Intercellular adhesion molecule 1*	1.68
	*ANXA2*	*Annexin A2*	1.62
	*ANXA11*	*Annexin A11*	1.74
	*ANGPTL2*	*Angiopoietin-like 2*	1.67
Down	*GHR*	*Growth hormone receptor*	2.12
	*HOXA10*	*Homeobox A10*	1.79
	*MUC13*	*Mucin 13 cell surface associated*	1.64
	*PGRMC2*	*Progesterone receptor membrane component 2*	1.54
**Group 3: Mechanisms of proliferation and differentiation**			
Up	*ABCB7*	*ATP-binding cassette sub-family B (MDR/TAP) member 7*	2.51
	*CSRNP1*	*Cysteine-serine-rich nuclear protein 1*	2.18
	*ANAPC7*	*Anaphase promoting complex subunit 7*	2.17
	*GADD45A*	*Growth arrest and DNA damage-inducible alpha*	2.02
	*ETS2*	*ETS proto-oncogene 2 transcription factor*	2.01
	*SRC*	*SRC proto-oncogene non-receptor tyrosine kinase*	1.97
	*ANAPC5*	*Anaphase promoting complex subunit 5*	1.95
	*NHEJ1*	*Nonhomologous end-joining factor 1*	1.90
	*PCNA*	*Proliferating cell nuclear antigen*	1.75
	*MAP2K1*	*Mitogen-activated protein kinase 1*	1.66
	*HIF1A*	*Hypoxia-inducible factor 1 alpha subunit (basic helix-loop-helix transcription factor)*	1.65
	*ABCB8*	*ATP-binding cassette. sub-family B (MDR/TAP) member 8*	1.61
	*ABCF1*	*ATP-binding cassette sub-family F*	1.60
	*ABCG2*	*ATP-binding cassette sub-family G (WHITE) member 2 (ABCG2)*	1.58
	*ANAPC16*	*Anaphase promoting complex subunit 16*	1.56
	*CD9*	*CD9 molecule*	1.54
	*AIFM2*	*Apoptosis-inducing factor mitochondrion-associated 2*	1.54
	*SDF2L1*	*Stromal cell-derived factor 2-like 1*	1.54
	*ABCD4*	*ATP binding cassette subfamily D member 4*	1.51
Down	*SCAPER*	*S-phase cyclin A-associated protein in the ER*	2.05
	*HOXA3*	*Sus scrofa homeobox A3*	1.99
	*POU2F1*	*POU class 2 homeobox 1*	1.84
	*HOXA10*	*Homeobox A10*	1.79
	*MARCH7*	*Membrane-associated ring finger (C3HC4) 7 E3 ubiquitin-protein ligase*	1.64

### Gene ontologies terms (GO)

DAVID functional annotations analysis was done using the official gene symbol as an identifier, with *Sus scrofa* as selected species, and the automatic defaults i.e. threshold: Count 2, and EASE 0.1. Among all of the evaluated DEGs 91.58% (658 up-regulated and 277 down-regulated) possessed identified DAVID ID (Supplementary Table 1). In further analyses, only DEGs possessing DAVID ID were used. Up-regulated genes were classified into 1 category within GO BP, 49 categories within GO CC, and 9 categories within GO MF. Down-regulated genes were classified into 5 categories within GO CC, and 5 categories within GO MF. The full list of GO terms within specific categories, classified genes, counts, *P*-values, fold enrichments, and statistical data are presented in Supplementary Table 2. The GO terms with the highest gene count within each GO direct are presented in Table 3.

**Table 3:** Selected based on the high gene count gene ontology (GO) terms evaluated in the myometrium of pigs during the peri-implantation period in response to peri-conceptional undernutrition. *BP* biological processes, *CC* cellular components, *MF* molecular functions.

GO term	Count	P-value	Fold enrichment	FDR
**Up-regulation**				
GO BP:0055114 Oxidation–reduction process	18	5.83E−05	3.12	1.06E−01
GO CC:0070062 Extracellular exosome	139	4.06E−19	2.14	1.67E−16
GO CC:0005829 Cytosol	55	9.42E−06	1.87	1.29E−03
GO CC:0005737 Cytoplasm	125	1.94E−05	1.42	1.59E−03
GO MF:0005525 GTP binding	29	1.06E−05	2.53	5.97E−03
GO MF:0003924 GTPase activity	19	3.53E−05	3.12	9.91E−03
GO MF:0044822 Poly(A) RNA binding	47	2.79E−04	1.73	5.22E−02
**Down-regulation**				
GO CC:0005654 Nucleoplasm	29	1.85E−04	2.13	3.80E−02
GO CC:0005829 Cytosol	20	9.59E−03	1.88	7.13E−01
GO CC:0031410 Cytoplasmic vesicle	5	1.62E−02	5.12	8.28E−01
GO MF:0000166 Nucleotide binding	12	1.55E−03	3.13	3.59E−01
GO MF:0046872 Metal ion binding	23	1.05E−02	1.76	8.11E−01
GO MF:0003682 Chromatin binding	10	1.55E−02	2.58	8.99E−01

### Biological pathways

Using a DAVID functional annotations analysis, evaluated up-, and down-regulated DEGs were classified into 50 Kyoto Encyclopedia of Genes and Genomes (KEGG)^28–30^ biological pathways, using automatic defaults i.e. threshold: Count 2, and EASE 0.1. The full list of biological pathways, classified genes, counts, *P*-values, fold enrichments, FDR, and Fisher exact data are presented in Supplementary Table 3. The largest number of counts was within the *metabolic pathway*, which is a global and overview map in KEGG. Here, it contained 102 counts (*P*-value = 0.00012, fold enrichment = 1.4, FDR = 0.01), i.a.* HMGCR*, encoding 3-hydroxy-3-methylglutaryl-CoA reductase, *COX2* encoding cyclooxygenase 2, *NSDHL* encoding NAD(P) dependent steroid-dehydrogenase-like, *HSD11B1* encoding hydroxysteroid (11*β*) dehydrogenase 1, and *HSD17B8* encoding hydroxysteroid (17*β*) dehydrogenase 8.

### Genes interaction network

Genes interaction networks were created for groups of DEGs indicated in Table 2. The analysis was performed with customized options, specifically a selection of 10 resultant genes, 10 resultant attributes, and Biological processes based GO weighting. The created gene interaction networks were limited to co-expression, co-localization, genetic interactions, and physical interactions, and are presented in Supplementary Figs. 1, 2, 3.

### Validation of microarray results

**Figure 1:**
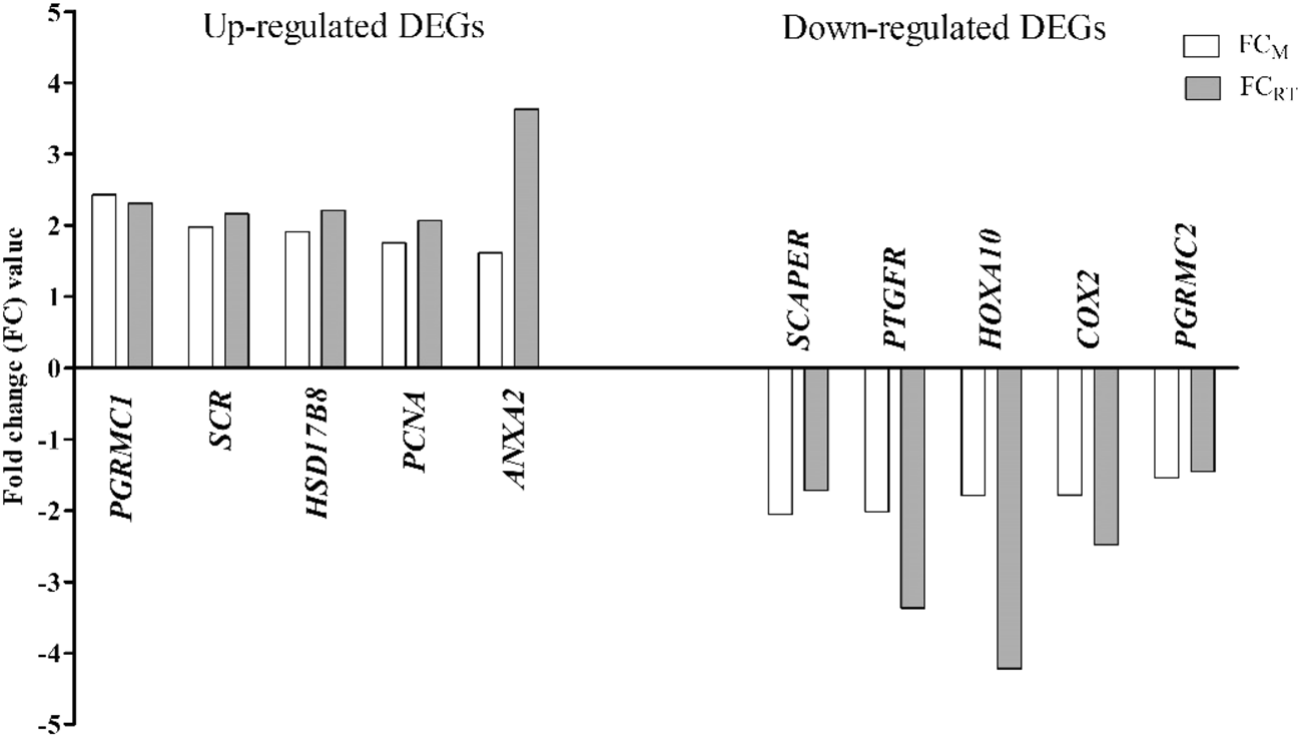
The validation of microarray results. The fold change values obtained with microarray analysis (FC_M_) and Real-Time PCR (FC_RT_) for validated up- and down-regulated genes are presented as bars. Figure generated in GraphPad Prism (trial version, https://www.graphpad.com/scientific-software/prism/).

The FC values obtained using microarray analysis (FC_M_) and Real-time PCR (FC_RT_) are presented in Fig. 1. The multiple regression coefficient (R) between FC_M_ values and FC_RT_ values was 0.947 (*P* ≤ 0.05), confirming that results obtained using microarray are validated.

### Transcription factor enrichment analysis (TFEA)

Using TFEA.CHiP tool based on the list of DEGs (Supplementary Table 1) there were evaluated 1154 TFs. After the removal of duplicates, the list was limited to 333 TFs. The VENN analysis showed 24 TFs that were common with DEGs. These were: *EP300, MAX, SMC3, NELFE, REST, SMARCB1, ETS2, KLF4, BDP1, ARID2, ARNTL, TCF7L1, FOXP2, SMAD2, CHD2, CREB1, ZNF449, RELA, HIF1A, TARDBP, XBP1, RAD21, PBX2, MEF2C*. Summary results are present in Supplementary Fig. 4, and Supplementary Table 4.

### Comparison of the myometrium, endometrium, and embryos transcriptomes of restricted-diet-fed gilts

**Figure 2:**
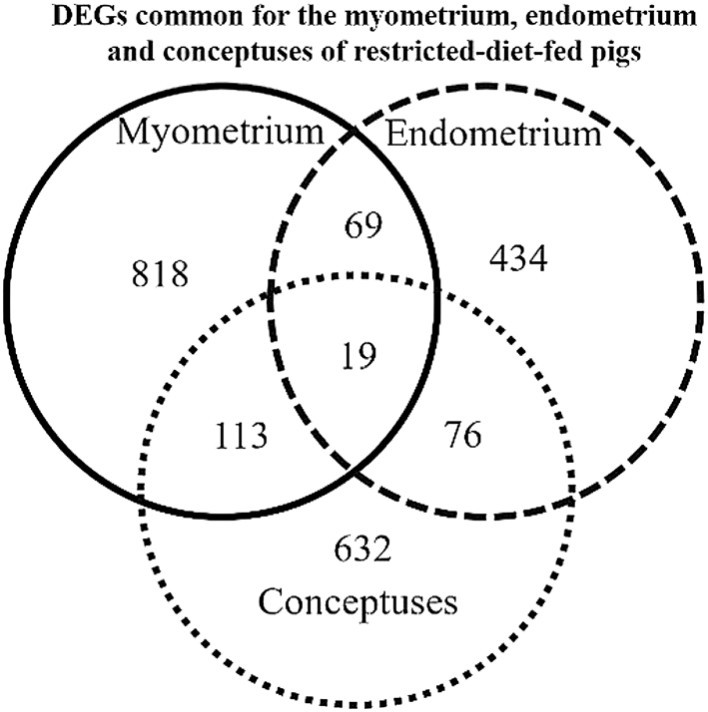
Venn diagram presenting the differentially expressed genes (DEGs) common for the myometrium, endometrium, and embryos collected from pigs during the peri-implantation period that were fed a restrictive diet during the peri-conceptional period compared to pigs fed a normal diet. Diagram generated with the use of online tool available at http://bioinformatics.psb.ugent.be/webtools/Venn/, with further modifications.

**Figure 3:**
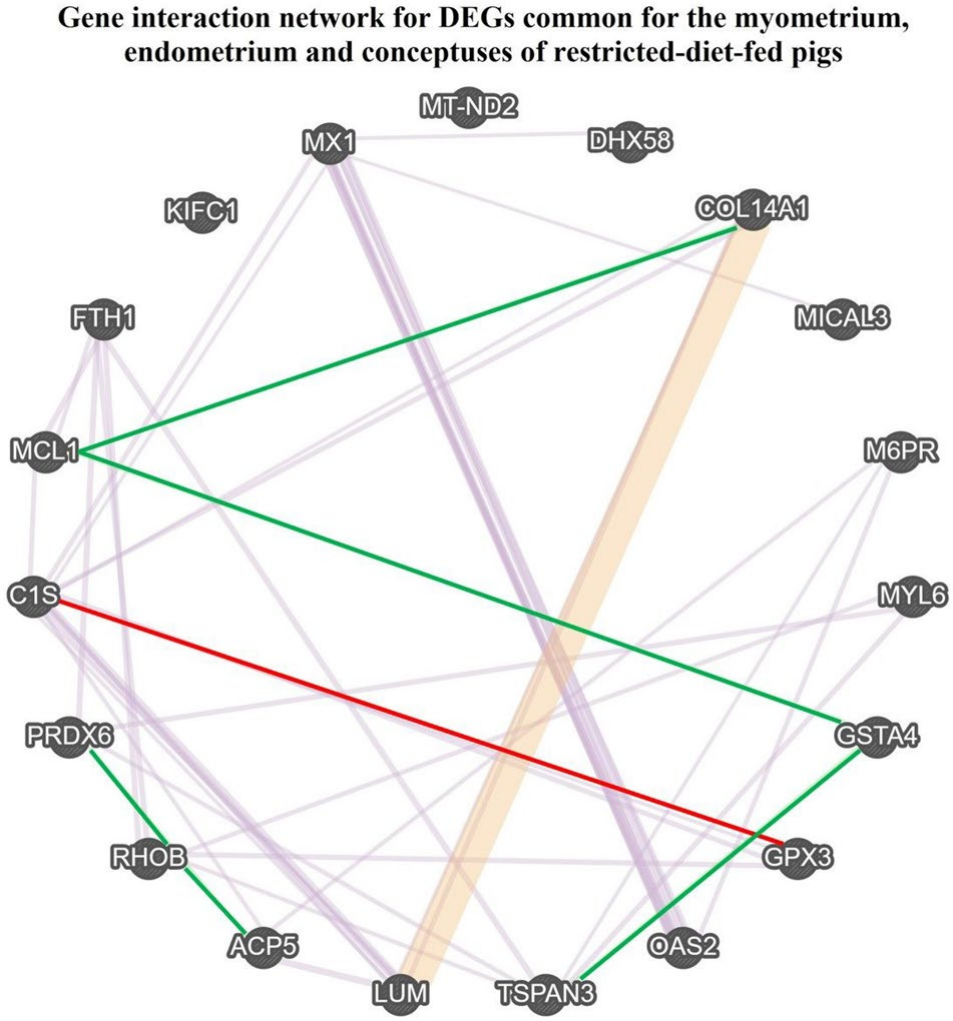
The gene interaction networks among common genes in the myometrium, endometrium, and embryos of pigs during the peri-implantation period that were differentially expressed (DEGs) in response to peri-conceptional undernutrition. Thread contacting visualized genes indicate specific gene interactions: red: co-localization, violet: co-expression, pink: physical interactions, green: genetic interactions. Figure generated in GeneMania Prediction server (https://genemania.org/) with modifications.

The dietary restriction during the peri-conceptional period resulted in the alterations in the transcriptomes of the myometrium, endometrium^18^, and embryos^27^ collected during the peri-implantation period. The Venn diagram is shown in Fig. 2. Among the evaluated DEGs, 19 were common for all studied tissues, 113 were common for myometrium and embryos, 69 were common for myometrium, and endometrium and 113 were common for endometrium 76 and embryos. Details are presented in Supplementary Table 5. The GO terms evaluated for DEGs common for myometrium, endometrium, and embryos are presented in Table 4. The interactions among DEGs common for the myometrium, endometrium, and embryos are presented in Fig. 3.

**Table 4:** Gene ontologies (GO) terms evaluated for DEGs common in the myometrium, endometrium, and conceptuses of pigs during the peri-implantation period that were fed a restrictive diet during the peri-conceptional period comparing to the myometrium of pigs during the peri-implantation period that were fed a normal diet during the peri-conceptional period. *CC* cellular components, *MF* molecular function.

GO term	DEGs	FDR	Fisher exact
GO CC: 0070062 Extracellular exosome	*ACP5; COL14A1; LUM; PRDX6; RHOB; TSPAN3*	0.55	0.0066
GO CC: 0048471 Perinuclear region of cytoplasm	*OAS2; M6PR; MX1*	0.55	0.0030
GO CC: 0005770 Late endosome	*M6PR; RHOB*	0.74	0.0020
GO MF: 0004602 Glutathione peroxidse activity	*GPX3; PRDX6*	0.75	0.0002
GO MF: 0003725 Double-stranded RNA binding	*OAS2; DHX58*	0.93	0.0015

## Discussion

The present study demonstrated that the short-lasting undernutrition in pigs during the peri-conceptional period alters the transcriptomic profile of the myometrium during the peri-implantation period. Specifically, as a consequence of the females’ nutritional restriction, the expression of 1021 genes encoding proteins with known biological functions has been changed ≥ 1.5-fold, and the majority of them, i.e. 69.34%, were up-regulated. Based on the results of TF enrichment analysis, there are 333 TFs that may be involved in the regulation of the expression of DEGs identified in the current microarray experiment. Interestingly, 24 of these TFs were differentially expressed in response to dietary restriction. Thus, the restriction diet used during the peri-conceptional period may alter the transcriptional activity of the myometrium by affecting the expression of TFs. In the current study, the highest number of gene count was within GO CC. Similar findings were demonstrated previously in the endometrium of restricted-diet-fed pigs^18^. Furthermore, among 50 evaluated KEGG biological pathways^28–30^ assessed in the myometrium of restricted-diet-fed pigs, the highest number of genes was classified to “*metabolic pathways*”, similarly as in the endometrium and conceptuses of restricted-diet-fed pigs^18,27^. In the conceptuses harvested from restricted-diet-fed pigs, the majority of DEGs were up-regulated (~ 80%), whereas in the endometrium above half of them (58%) were down-regulated^18,27^. Thus, the transcriptomic activity in the myometrium and conceptuses during the peri-implantation period in response to dietary restriction during the peri-conceptional period may be mostly up-regulated, while in the endometrium it is rather down-regulated.

In this study, we have selected day 9 as the cut-off of under-nutrition protocol based on epigenetic events, namely methylation profile establishment, occurring during the early stages of pregnancy^31^. Until reaching the blastocyst stage, which occurs approximately at day 9 in this species, the embryonic genome undergoes active and passive demethylation^31^. In consequence of the disruptions in genome-wide methylation and demethylation, embryos are likely to develop with less than fully penetrant phenotypes^31^. It is known that both restricted and excessive maternal dietary protein intake during pregnancy alter the offspring's epigenetic marks and influence the expression of genes^32^. Moreover, DNA demethylation mechanisms act during early embryonic development, and a drastic reduction of methylation levels is expected to be found at later preimplantation stages^31^. Providing that DNA methylation is one of the mechanisms regulating the transcriptomic activity, the selection of a peri-conceptional period for dietary restriction for further transcriptome profiling during later stages of pregnancy is justified. We believe that the myometrium possesses “epigenetic sensitivity”, mainly during intensive changes occurring during early pregnancy, and the epigenetic alterations, including DNA methylation, are one of possible mechanisms underlying the diet-related alterations in the transcriptomic activity of the uterus.

The previous studies determined that the myometrium is an endocrine tissue, and its secretory activity differs regarding the physiological status of the female (pregnant vs. estrous cyclic pigs) and in the following days of pregnancy and the estrous cycle^22,23,26,33–35^. These alterations contribute to the creation of a specific, intrauterine milieu, which is critical for proper embryo-maternal dialog during early pregnancy^36,37^. It is worth highlighting that our previous study showed that restricted-diet fed gilts exhibit a decreased intrauterine concentration of E_2_^17^. It is known that in pigs E_2_ is one of maternal recognition of pregnancy signal^38^. Thus, the decrease in its concentration may deteriorate the developmental potential of embryos and impact mechanisms of pregnancy establishment. Notably, E_2_ is also an important regulator of transcriptional activity of the uterus^39^. Thus, the alterations in the intrauterine concentration of E_2_ evoked by dietary restriction^17^ could stand for an additional possible mechanism underlying the observed diet-related alterations in the transcriptional activity observed in the current and previous study^18^.

Among the evaluated DEGs in the myometrium of restricted-diet-fed pigs, there was *HMGCR*, encoding the 3-hydroxy-3-methylglutaryl coenzyme A reductase (HMGCR) classified to some of evaluated KEGG pathways^28–30^, i.a. metabolic pathways and also to some GOs, i.e. GO BP: oxidation–reduction process (GO:0055114), GO CC: endoplasmic reticulum membrane (GO:0005789), and GO CC: peroxisomal membrane (GO:0005778). The HMGCR is the rate-limiting enzyme in the biosynthesis of cholesterol and isoprenoids, which are substrates required for post-translational modifications of proteins involved in a signaling pathway that regulates embryonic development (for rev. Eisa-Beygi et al.^40^), and for steroid hormones biosynthesis pathway^41^. Respecting that porcine myometrium possesses an active steroidogenic pathway^22–24,26,34^, the observed increased expression of *HMGCR* in the myometrium of the restricted diet-fed pigs may lead to a higher supply of cholesterol, for further synthesis of steroid hormones in the myometrium. However, the previous studies demonstrated that the dietary restriction during the peri-conceptional period results in the decrease of the total content of E_2_ in the uterine flushings during the peri-implantation period^17^. This finding might be underlaid by previously determined diet-related up-regulation of endometrial *HSD17B4*^18^, encoding hydroxysteroid (17*β*) dehydrogenase type 4 (17*β*HSD4), which converts estradiol-17*β* (E_2_) to estrone (E_1_)^42^. Based on the current results, the expression of *HSD17B4* was not altered in the myometrium of restricted-diet-fed pigs, but there was observed an up-regulation of the *HSD17B8* gene. This gene codes for 17*β*HSD type 8 catalyzing the interconversion of androstenedione (A_4_) to testosterone (T), and E_1_ to E_2_. Thus, the diet-related decrease in the uterine content of E_2_ is dependent rather on endometrial than myometrial steroidogenic activity.

Interestingly, *HMGCR* is co-expressed and co-localized with *HSD11B1*, coding for 11*β*-hydroxysteroid dehydrogenase type 1 (11*β*HSD). The 11*β*HSD participates in the conversion of cortisone to cortisol^43,44^. Therefore, the observed in the current study up-regulation of *HSD11B1* in the myometrium indicated the potential of the tissue to produce cortisol. Interestingly, it was previously found that cortisol may decrease the release of luteolytic prostaglandin F_2_α (PGF_2_α) from the endometrium^45^. The expression of *cyclooxygenase 2* (*COX2*), *prostaglandin F synthase* (*PGFS*), and *prostaglandin F receptor* (*PTGFR*) were previously demonstrated in the myometrium as well as the active PGF_2_α release by this tissue in vitro^21,46^. The current study has shown that dietary restriction leads to the decreased expression of *COX2* and *PTGFR* in the myometrium. Thus, the action of PGF_2_α in the myometrium of restricted-diet-fed pigs may be disturbed. The peri-implantation period is accompanied by a molecular dialog between conceptuses and the uterus. This phenomenon can be considered as a semi-inflammatory process^47^, and PGF_2_α is recognized as a proinflammatory and immunostimulatory factor^48^. Moreover, PGF_2_α regulates the expression of genes potentially important for embryo-maternal interactions^49^. Disturbances in the potential of PGF_2_α synthesis and its action in the myometrium of restricted-diet-fed pigs expressed as the decreased *COX2* and *PTGFR* expression may affect the PGF_2_α-mediated process occurring during the peri-implantation period, which can be detrimental for pregnancy success.

The proper embryo-maternal interactions require signals triggered by P_4_^50^. Interestingly, the current study documented that the restricted diet resulted in the alterations in the expression of *progesterone membrane components*, i.e.* PGRMC1* (up-regulated) and *PGRMC2* (down-regulated). Furthermore, PGRMC1 mediates P_4_-dependent control of the cell cycle, proliferation, and apoptosis, whereas PGRMC2 was found to inhibit cell migration^51–53^. Notably, the decreased expression of *PGRMC2* was detected also in porcine conceptuses collected from restricted-diet-fed pigs^27^. The alterations of *PGRMCs* expression in response to the dietary restriction may affect the non-genomic action of P_4,_ and impact cell cycle, proliferation, and apoptosis of myometrial cells. Uterine development and morphogenesis during early pregnancy are also regulated by homeobox A family proteins (HOXA)^54^. Previous studies demonstrated that the expression of *HOXA10* and *HOXA13* is increased in the myometrium of early pregnant pigs^46^. Thus, the decreased expression of *HOXA3* and *HOXA10* in the myometrium of restricted diet-fed pigs may affect cell cycle, myocyte proliferative capacity and differentiation, and development of the myometrium required for proper development of the uterus during pregnancy.

Notably, the restricted diet during the peri-conceptional period evokes the alteration in the several genes encoding proteins involved in the regulation of the cell cycle, proliferation, and differentiation. This study evaluated that the expression of genes encoding *anaphase-promoting complex* subunits, i.a. *ANAPC5*, *ANAPC7*, *ANAPC16*, and the expression of *proliferating cell nuclear antigen* (*PCNA*) and *SRC proto-oncogene, non-receptor tyrosine kinase* (*SRC*) was significantly increased, while the expression of *S-phase cyclin A-associated protein in the endoplasmic reticulum* (*SCAPER*) was decreased. The genes encoding ANAPC is classified to the GO CC term “anaphase-promoting complex” (GO:0005680). The ANAPC regulates the proper chromosome segregation during mitosis, mitotic exit, and the maintenance of the G1 phase of the cell cycle^55^. PCNA may be considered as a regulator of DNA replication, while SRC is involved in the regulation of proliferation, differentiation, and cellular viability^56,57^. It was found that the decreased content of SCAPER contributes to the decreased level of cytoplasmic cyclin A resulting in the delay of G1/S phase transition during the cell cycle^58^. Thus, the dietary restriction in females during the peri-conceptional period may determine the myometrial cells' fate during the peri-implantation period.

The current study demonstrated that the restricted diet during the peri-conceptional period also leads to the up-regulation of *ANXA2* (annexin 2) in the myometrium of pigs during the peri-implantation period. The *ANXA2* is classified to many GO terms among GO CC, *i.a.* extracellular exosome (GO:0070062), cytosol (GO:0005829), extracellular matrix (GO:0031012), and membrane raft (GO:0045121), and among GO MF, poly(A) RNA binding (GO:0003723). In general, annexins are a family of proteins involved in many cellular functions, including vesiculation and cell membrane repair, and ANXA2 induces membrane folding and blebbing initiated from membrane structural defects^59^. The previous studies demonstrated that myometrial expression of *ANXA2* was greater in pigs during the peri-implantation period than during luteolysis^46^. In the current study, the abundance of the *ANXA2* mRNA transcript was found to be greater in the myometrium of the restricted-diet-fed pigs. Further bioinformatical analysis revealed that *ANXA2* is co-expressed with genes encoding other annexins (*ANXA1* and *ANXA11*), intercellular adhesion molecules (*ICAM1*, *ICAM2*, and *ICAM4*), and platelet/endothelial cell adhesion molecule 1 (*PECAM1*), which exhibited up-regulated expression in the myometrium of restricted-diet-fed gilts. Interestingly, these genes encode for proteins involved in processes of embryo apposition and adhesion to the uterine wall as well in the induction of angiogenesis^59,60^. Thus, the mild nutritional restriction during the peri-conceptional period may affect the mechanisms determining the myometrial integrity and angiogenesis during the peri-implantation period.

This study assessed the transcriptomic alterations in the myometrium (the current study), endometrium^18^, and conceptuses^27^ collected from restricted-diet-fed pigs. Among DEGs which are common for uterine tissues and conceptuses there is *MCL1 apoptosis regulator, BCL2 family member* (*MCL1*), which expression in restricted diet-fed pigs was increased in the myometrium (the current study) while decreased in the endometrium and embryos^18,27^. The MCL1 is considered an anti-apoptotic protein, and its down-regulation leads to the activation of caspases involved in apoptosis^61^. Thus, the interesting phenomenon of increased gene expression in the myometrium and decreased gene expression in the endometrium and embryos in response to the restricted diet may affect the rate of apoptosis during the peri-implantation period. Furthermore, in the myometrium (the current study), the endometrium^18^, and embryos^27^ of the restricted diet-fed pigs there was observed the up-regulation of *ACP5*, which is involved in the transport of iron from the endometrium to embryos^62^. The *ACP5* is classified to the “extracellular exosome” (GO:0070062) GO CC term. This finding indicates that the restricted diet may potentially affect the uterine and conceptuses' capacity to produce the extracellular vesicles that may participate in the embryo-maternal cross-talk^63^. It is worth highlighting that both *ACP5* and *FTH1*, which is the gene encoding ferritin heavy chain 1, were up-regulated in the myometrium (the current study), the endometrium^18^ and conceptuses^27^ of restricted diet-fed pigs. Interestingly, FTH1 is a major intracellular iron storage protein that may function in the protection from oxidative stress^64^. Thus, the up-regulation of *FTH1* and *ACP5* genes in uterine tissues and conceptuses may lead to the altered distribution of iron in embryo-uterine interspace. Thus, the described alterations in the expression of *ACP5, FTH1*, *GSTA4* and *GPX3,* occurring in the uterine tissues and conceptuses of restricted-diet-fed pigs, may also be considered as mechanisms of intrauterine protection from oxidative stress.

For a better understanding of the mechanisms underlying changes in the transcriptional activity of the myometrium occurring in response to a restricted diet, we have identified TFs casually responsible for changes in the expression of the evaluated DEGs. Indication of TFs which may be involved in the regulation of the transcriptional activity of the evaluated DEGs is promising, but still challenging and show that applied undernutrition protocol may evoke a broad spectrum of transcriptomic alterations. Notably, the peri-conceptional undernutrition caused alterations in the transcriptional activity of 24 of the evaluated TFs. Among these differentially expressed TFs, there is *KLF4*, encoding for Krüppel-like factor 4. KLF4 is involved in the regulation of numerous biological processes including cell development and cell division, apoptosis, and cell programming, and its down-regulation is often coupled with tumor progression^65^. This study showed that nutritional restriction increases the transcriptional activity of *KLF4* in the myometrium. Importantly, the previous studies showed that an up-regulation of *KLF4* induces cell cycle arrest^65^. Having in mind that this study evaluated diet-related alterations in the transcriptional activity of genes coding for proteins involved in the regulation of cell cycle and determining the proliferative potential of the myometrium, i.a.* PGRMC* genes, *HOXA* genes, *ANAPC* genes, *SRC*, *SCAPER*, *PCNA*, and these changes may be connected with the altered expression of *KLF4*. Among differentially expressed TFs there was also *RELA* coding for RELA Proto-Oncogene, NF-KB Subunit. *RELA* is a part of the nuclear factor kappaB (NF-kappaB) family, regulating the expression of i.a. pro-inflammatory mediators. The expression of *RELA* was increased in the myometrium in response to dietary restriction. It is worth highlighting that pregnancy might be recognized as a semi-inflammatory process where the action of cytokines, tumor necrosis factor (TNF), and COX2 is crucial for proper implantation^47^. In this study, we observed diet-related down-regulation in *COX2* expression, and this alteration might be connected with the altered expression of *RELA.* However, the applied undernutrition protocol did not contribute to alterations in the expression of pro-inflammatory cytokines and TNF what suggests that there must appear additional mechanism controlling the transcriptional activity of the myometrium of restricted-diet-fed pigs. One may not exclude that this mechanism relies on epigenetic mechanisms, including DNA methylation, but this assumption needs further investigation.

In conclusion, the observed in the current study alternations in the transcriptomic profile of the myometrium show that the short-lasting 30% decrease of food intake by pregnant females during the unique peri-conceptional period may disturb the proper course of biological processes occurring in the myometrium during the peri-implantation period. Among the observed alterations, increased potential for the synthesis of steroid hormones, decreased potential for prostaglandins synthesis and action, decreased proliferative capacity, and increased adhesion among myocytes were determined. The observed transcriptomic alterations of the myometrium might jeopardize proper implantation and thus, pregnancy success, but this requires further in vivo studies. Moreover, the results documented that the transcriptomes of the myometrium, endometrium, and conceptuses during the peri-implantation period respond differently to female dietary restriction during the peri-conceptional period.

## Methods

### Ethics statement

All experiments were approved by the Animal Ethics Committee, University of Warmia and Mazury in Olsztyn, Olsztyn, Poland—decision No. 54/2015/DTN. The study was carried out in compliance with the ARRIVE guidelines^66^, and all methods were carried out following relevant guidelines and regulations indicated where appropriate.

### Animals, feeding protocol, and collection of myometrial tissue

The post-pubertal pigs (*Sus scrofa Domestica *L., Polish Landrace × Great White Polish, weighing 95 to 110 kg) were naturally bred on the first and second day of estrus of their second estrous cycle. Gilts assigned to the control group (n = 4) were fed a normal diet, i.e. daily 3 kg/gilt of forage since the first sign of the estrus, during natural mating, and until slaughter. Gilts assigned to the experimental group (n = 4) were fed a restricted diet, i.e. daily 2.1 kg/gilt of forage, which is a 30% food restriction, during the peri-conceptional period (the first sign of the estrus, during mating, and until 9th day of pregnancy), and next fed a normal diet until slaughter.

The composition of forage, feeding procedures and housing of animals were described previously, and the effectiveness of the applied protocol of undernutrition was verified by analyzing the biochemical parameters of blood plasma^17,27,67^. The animals did not lose weight. Briefly, there was observed a diet-related decrease in the concentration of total phosphorus, calcium, and total cholesterol, and a tendency to increase in the concentration of triglycerides in restricted-diet fed pigs^67^. Alterations in the concentration of total cholesterol and triglycerides in blood plasma indicate alterations in lipid metabolism, which is a visible sign of altered nutritional status in pigs fed a restricted diet. Moreover, there was indicated that dietary restriction led to the increase in the concentration of aspartate aminotransferase in blood plasma, a marker of liver damage, which is one more sign that applied dietary restriction affected the wellbeing of these females^67^. We did not observe any diet-related effect on conception success nor the morphology of conceptuses.

Gilts from both groups were slaughtered in a local abattoir (Rozdroże, Poland) during the peri-implantation period, specifically on day 16 after the first mating. Respecting the mating protocol and effectiveness of mating, the animals were on day 15 to 16 of pregnancy. Immediately after slaughter uteri were collected and placed into ice-cold phosphate-buffered saline (PBS) containing 100 IU mL^−1^ penicillin (Polfa, Poland) and 100 IU mL^−1^ of streptomycin (Sigma Aldrich, St. Louis, MO, USA), and transported to the laboratory. The day of pregnancy was confirmed by the morphology of ovaries and conceptuses flushed from uterine horns as well as the concentration of P_4_ in peripheral blood^33,68–71^. Next, uterine horns were opened longitudinally, the perimetrium tissue was discarded by careful scraping, and the myometrial cross-sections were collected with scissors and snap-frozen in liquid nitrogen (− 196 °C). Samples were stored at – 80 °C until further analysis.

### RNA isolation

RNA was extracted from 30 mg-weighting myometrial slices using 500 mL of TRI Reagent (Sigma Aldrich, Germany). Samples were initially homogenized on ice using a homogenizer TissueRuptor (Qiagen, Germany). Then, a 150 µL of chloroform (POCH, Poland) was added, samples were mixed thoroughly and incubated for 10 min at 4 °C. Next, samples were centrifuged 12,000×*g*, for 10 min at 4 °C and the supernatant was transferred to a 350 µL of propane-2-ol (Sigma Aldrich, Germany) beforehand chilled at − 20 °C. Next, samples were mixed well by vortexing and incubated for 15 min on ice assuring brief mixing every 3 min. Next, samples were centrifuged 12,000×*g*, for 10 min at 4 °C, and the supernatant was discarded. RNA pellets were washed twice with 1 mL of 75% ethanol (Chempur, Poland) beforehand chilled at − 20 °C. The RNA pellets were suspended in 30 µL of DEPC-treated water (Sigma Aldrich, Germany). The initial analysis of concentration and purity of extracted RNA was determined using Tecan with *i*-control software (Tecan, Switzerland), and the initial test for RNA integration was performed using gel electrophoresis. Samples with a concentration higher than 100 ng/µL, optical density (OD) from 1.8 to 2.0, and integral electrophoretically were used in the next steps of analysis. The samples that fulfilled these criteria were tested using microcapillary gel electrophoresis in 2100 Bioanalyzer (Agilent Technologies, USA) to determine RNA integrity number (RIN). Samples with RIN ≥ 7.5 were used for microarray analysis.

### Microarray data analysis and differentially expressed genes

The microarray analysis was performed using RNA samples isolated from the myometrium collected from restricted-diet-fed pigs (experimental group, n = 4) in comparison to the normal-diet-fed pigs (control group, n = 4) using Porcine (V2) Expression Microarrays 4 × 44 K (Agilent Technologies, USA) according to the Two-Color Microarray-Based Gene Expression Analysis Protocol (Agilent Technologies, USA). Four independent biological replicates per group, i.e. experimental and control group, allowed to obtain an experiment power of 80% and a false discovery rate (FDR) of 0.1^72^. The specific workflow of the experiment was described previously^18,27,46^. Briefly, 400 ng aliquots of RNA were used to synthesize cRNAs samples labeled with CY3 or CY5. Fluorochrome-labeled cRNA was purified on RNeasy Mini Spin columns (Qiagen, USA). After determination of cRNA concentration and labeling efficiency, samples were conducted through the cRNA fragmentation step and mixed with a hybridization buffer. Then, samples were applied on microarrays following the classic class comparison experiment design, using a dye-swap to prevent dye bias^73,74^. After hybridization (for 17 h at 65 °C), microarrays were washed, dried, and scanned using Agilent High-Resolution C Microarray Scanner (Agilent Technologies, USA). Fluorescence intensity was calculated using Agilent Feature Extraction software *v.* 10.5.1 (Agilent Technologies, USA), the outlier spots were filtered, and the background correction and dye normalization (linear and LOWESS) were performed. The obtained data were analyzed using GeneSpring GX 13.0 software (Agilent Technologies, USA). The transcripts with fold change (FC) ≥ 1.5 and *P* ≤ 0.05 (Student’s t-test, default statistical model) were aligned with the *Sus scrofa* transcriptome (txid9823) using the BLAST tool for identification of encoded gene name. When more than one differentially expressed transcript was assigned to one gene name, the FC and *P*-values of these transcripts were averaged and recognized as one differentially expressed gene (DEG). DEGs that were found in both up-regulated and down-regulated gene lists were omitted in further analyses.

### Enriched gene ontology (GO) terms and biological pathways

The full list of DEGs was first divided into two lists: a list of up-regulated DEGs and down-regulated DEGs. Both lists were uploaded separately to Database for Annotation, Visualization and Integrated Discovery (DAVID) *v.* 6.8 (accession June 2021)^75,76^, annotations were limited to *Sus scrofa* and DAVID IDs of uploaded DEGs were determined. DEGs with determined DAVID ID were classified into gene ontology (GO) terms within biological processes (BP), cellular components (CC), and molecular functions (MF) within GO Direct. DAVID GO IDs were defined using the Quick GO database (http://www.ebi.ac.uk/QuickGO). Then, up- and down-regulated DEGs were uploaded together to DAVID for further visualization of KEGG pathways^28–30,77,78^. In the analyses, only GO terms and KEGG pathways with FDR-adjusted *P*-values ≤ 0.05 were considered.

### Interaction network of selected genes

The list of evaluated DEGs was then used to create groups of DEGs encoding proteins involved in the: 1/The endocrine activity, 2/Embryo-maternal interactions, and 3/Mechanisms of proliferation and differentiation. For these groups, the gene interaction networks were created with GeneMANIA prediction server^79^, using *Homo Sapiens* as a reference genome. GO-BP-based weighting was used to determine the interactions within co-expression, co-localization, genetic interaction, and physical interaction categories, and each network was enriched with 10 resultant genes.

### Validation procedure

RNA samples used for microarray analysis were used for cDNA synthesis using QuantiTec Reverse Transcription Kit (Qiagen, USA) following the protocol provided by the manufacturer and rules described by Bustin et al.^80^. For validation procedure, there were selected five up-regulated and five down-regulated genes expressed in the myometrium of restricted-diet-fed pigs. cDNA samples were amplified with POWER SYBR Green PCR Master Mix in AriaMx apparatus with AriaMX Software *v.* 1.3 (Agilent Technologies, USA). When available, primer sequences were obtained from references [*actin beta*, *ACTB*^81^, *RNA, 18S ribosomal*, *RNA18S*^19^, *hydroxysteroid (17β) dehydrogenase 8*, *HSD17B8*^82^, *homeobox A10*, *HOXA10*^83^, *cyclooxygenase 2*, *COX2*^33^], or were designed using BLAST tool provided by NCBI. The reaction parameters were: 1/initial denaturation for 10 min at 95 °C (one cycle), 2/denaturation for 15 s at 95 °C, primer annealing for 1 min, at temperature specific to primers set (Supplementary Table 6), and elongation for 1 min at 72 °C (40 cycles) followed by dissociation step (one cycle). The expression level of amplified genes was calculated using the *∆∆*Ct method. For each tested gene, the absolute value of the ratio of the mean expression of the tested gene in the control group i.e. fed a normal diet *vs.* the mean expression of the tested gene in the experimental group, i.e. fed a restricted diet during the peri-conceptional period were used to calculate FC values (Eq. 1).

1 $${FC}_{RT}=|\frac{\overline{x}{2 }^{-\Delta \Delta {Ct}_{control}}}{\overline{x}{2 }^{-\Delta \Delta {Ct}_{experimental}}}|$$

Calculation of fold change (FC) values for data obtained with Real-Time PCR (FC_RT_).

$\overline{x }$ 2^−∆∆Ct^ control—mean expression of the tested gene in the control group, $\overline{x }$ 2^−∆∆Ct^ experimental—mean expression of the tested gene in in the experimental group.

The FC values obtained after Real-Time PCR (FC_RT_) and FC values obtained after the microarray experiment (FC_M_) were used to calculate multiple regression coefficients (R)^84^.

### Transcription factor enrichment analysis (TFEA)

Transcription factor (TF) enrichment analysis was performed using human TF dataset with rank test Kolmogorov–Smirnov rank test, and the TFEA.CHiP tool (https://bio.tools/TFEA.ChIP)^85^, and a GSEA-like analysis method. There were selected default options: source organism: human, plot type: Enrichment Scores ChiP-Seq source database: ReMap2020 & GeneHancer Double Elite. The output list of TFs was screened for duplicates, which were removed from further analysis. Next, the VENN diagram was created using lists of the TFs and DEGs to evaluate TFs that were differentially expressed (DE) in the myometrium of restricted-diet-fed pigs.

### The comparison of the transcriptomic profiles of the myometrium, endometrium, and embryos of restricted-diet-fed gilts: Venn diagram

The lists of DEGs evaluated in the myometrium (the present study), endometrium^18^, and conceptuses^27^ collected from restricted-diet-fed pigs were used to create a Venn diagram. The DEGs common for the myometrium, endometrium, and embryos of restricted diet-fed gilts were then used to create an interaction network using GeneMania software as described in section “Interaction network of selected genes”.

## Supplementary Information

Supplementary Figure 1.

Supplementary Figure 2.

Supplementary Figure 3.

Supplementary Figure 4.

Supplementary Table 1.

Supplementary Table 2.

Supplementary Table 3.

Supplementary Table 4.

Supplementary Table 5.

Supplementary Table 6.
