# Supplementary material for: Nutritional restriction during the peri-conceptional period alters the myometrial transcriptome during the peri-implantation period
Source: Sci Rep. 2021 Oct 27;11:21187. doi: 10.1038/s41598-021-00533-x (PMC8551329; doi:10.1038/s41598-021-00533-x)
Supplement: Supplementary file 3 — Supplementary Figure 3. [file 41598_2021_533_MOESM3_ESM.pdf]

Nutritional restriction during the peri-conceptional period alters the myometrial transcriptome during the peri-implantation period

Ewa Monika Drzewiecka[1], Wiktoria Kozlowska[1], Agata Zmijewska[1], Anita Franczak[1, *]

[1] Department of Animal Anatomy and Physiology, University of Warmia and Mazury in Olsztyn, Oczapowskiego 1A, 10-719 Olsztyn, Poland; * Corresponding author: anitaf@uwm.edu.pl, tel. +48 89 523-42-18

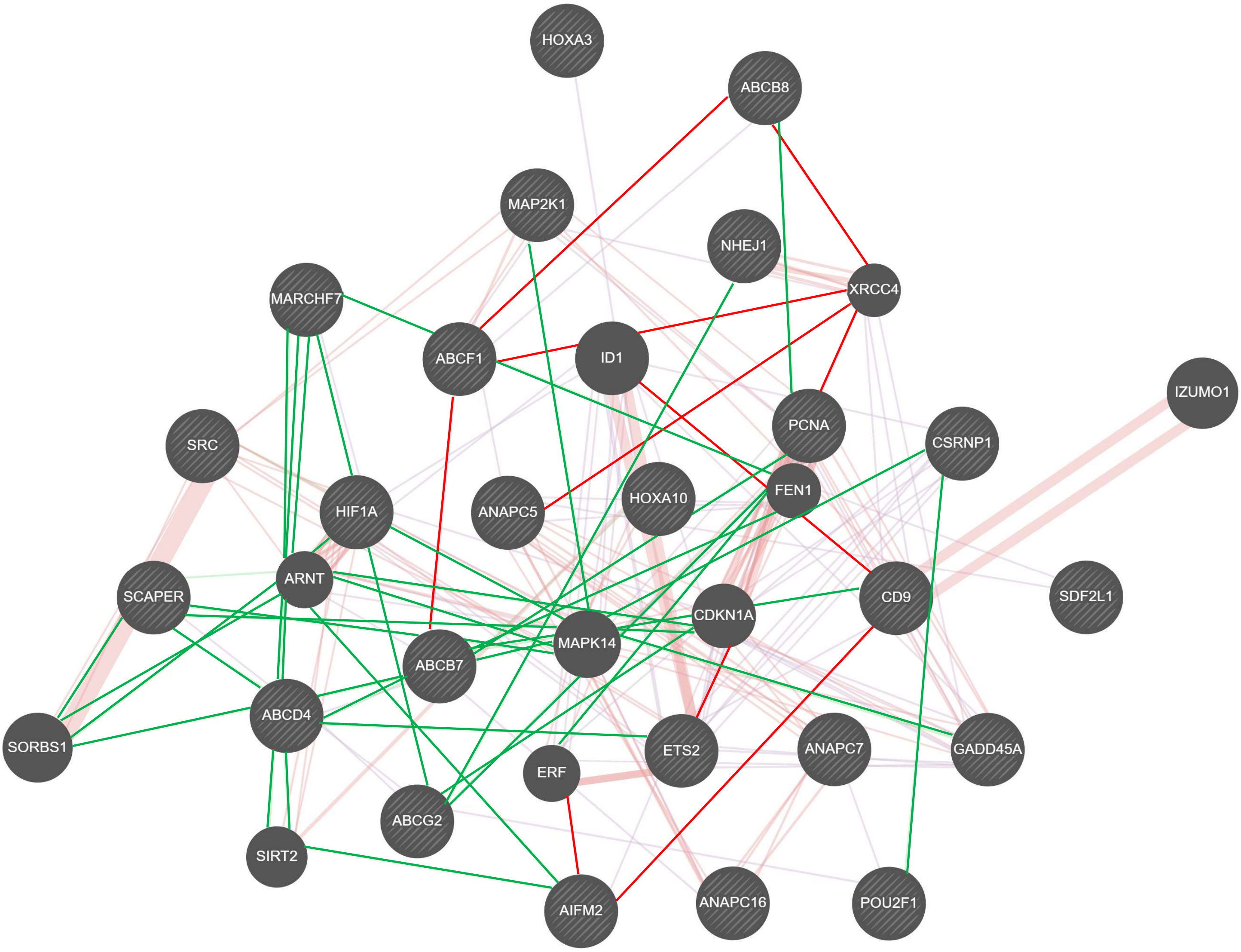

Supplementary figure 3. The gene interactions among differentially expressed genes (DEGs) in the myometrium of pigs during the peri-implantation period fed a restricted diet during the peri-conceptional period, classified to the group of genes involved in the regulation of cell cycle, proliferation and tissue morphogenesis *i.e.* Mechanisms of proliferation and differentiation. DEGs are presented in hatched circles, and resultant genes automatically added in GeneMania prediction server are presented in clear circles. Nodes indicate specific gene interactions: red: co-localization, violet: co-expression, pink: physical interactions, green: genetic interactions. Figure generated in GeneMania Prediction server (https://genemania.org/) with modifications.
