# Supplementary material for: Nutritional restriction during the peri-conceptional period alters the myometrial transcriptome during the peri-implantation period
Source: Sci Rep. 2021 Oct 27;11:21187. doi: 10.1038/s41598-021-00533-x (PMC8551329; doi:10.1038/s41598-021-00533-x)
Supplement: Supplementary file 4 — Supplementary Figure 4. [file 41598_2021_533_MOESM4_ESM.pdf]

Nutritional restriction during the peri-conceptional period alters the myometrial transcriptome during the peri-implantation period

Ewa Monika Drzewiecka[1], Wiktoria Kozlowska[1], Agata Zmijewska[1], Anita Franczak[1, *]

[1] Department of Animal Anatomy and Physiology, University of Warmia and Mazury in Olsztyn, Oczapowskiego 1A, 10-719 Olsztyn, Poland; * Corresponding author: anitaf@uwm.edu.pl, tel. +48 89 523-42-18

Full list of evaluated TFs

CREB3L1, E2F8, HIVEP1, NFE2, ZNF189, ID3, ZFP28, ZEB1, RFX5, ERG, SMARCC2, SMARCC1, KLF11, MIER1, USF2, IRF1, NANOG, TRIM22, CHD1, TAF7, BRCA1, RUNX3, RXRA, NFATC4, ZNF623, NFATC1, HES1, ZNF197, ISL1, SIX5, ZNF274, ATF3, SIRT6, BCLAF1, IKZF1, FOXM1, ZBTB48, EZH2, FOXA2, ARNT, POL2, SETDB1, NFKB2, ARID3A, MAFK, ID1, ZNF41, ZNF35, HMGN3, MEF2A, ZBTB11, HSF2, ESRRA, STAT4, IRF3, E2F1, THAP1, GATAD2A, KDM5A, ATF1, ZBTB18, TCF12, GLIS2, ZNF641, PPARG, MYNN, TP73, ZNF639, MYBL2, NR2C2, STAT5A, EGR1, ZNF350, NFYA, GATA1, AHR, NFE2L2, KLF6, NRF1, NR3C1, MYOD1, BRF1, ARNTL2, YY1, PPARGC1A, WT1, ZNF146, TEAD2, JUND, AFF4, ZNF281, ZNF419, RFX1, TFE3, POLR3G, CREB3, ETV4, STAT3, ZNF792, MBD4, EBF3, ZNF561, HOXA5, SOX17, ELF4, ELF1, ZNF217, EBF1, SALL2, HNF4G, ATF5, E2F4, ZBTB7A, ESR1, ZNF565, SIN3A, FLI1, ZNF134, NFATC3, ZKSCAN1, SMAD4, BCL6, ZNF480, TBP, GLI3, NFIC, MYC, CCNT2, ARID1B, E2F7, GTF3C2, ZFP82, SP4, POU2F2, ZBTB4, FOXO1, CTCF, STAT1, MAZ, GABPA, PBX3, ZNF260, CREB3L4, BATF, CREM, ELK4, ZIK1, NR2F2, PRDM1, TEAD4, CREBL2, ZBTB14, ZNF287, VDR, MLX, IRF4, ZNF548, NR3C2, MYBL1, ZNF45, ZNF140, DBP, FOSL2, KDM5B, HNF1A, RARA, POLR3A, RCOR1, MIXL1, PML, PPARA, GATA3, RORA, ELK1, KLF7, HIC1, MTA3, FOXO4, SPI1, HOXB4, SP1, ZBTB33, GTF2F1, MXI1, TAF1, NR4A1, FOXA1, PDX1, ZNF322, DDIT3, FOS, JUNB, SMARCA4, ZNF211, SAP30, MEOX2, ATF2, TFAP2C, MSX1, TBL1XR1, NR5A2, HDAC8, SIX4, TAL1, NCOR1, SP2,TBX3, HDAC2, ETS1, ZNF121, MYCN, NCOA1, GTF2B, HNF4A, SUZ12, ELF2, HOXA4, BHLHE40, TRIM28, RB1, HSF1, ZEB2, HEY1, MTA1, CEBPA, TFAP2A, IRF2, USF1, POLR2A, NR2F6, MAFB, PHF8, SUPT20H, E2F2, ZSCAN31, CAMTA2, ZNF143, ZNF263, TCF3, BACH1, STAT2, TP53, CTBP2, CBX3, DEAF1, RELB, HNF1B, DRAP1, RBBP5, TCF7L2, NONO, ZNF567, GATA2, HINFP, NFYB, E2F6, ZFHX3, POU5F1, TFEB, YBX3, HDAC1, NFKB1, BCL3, ZKSCAN3, ZNF789, ZNF613, BRF2, BCL11A, WRNIP1, ZNF2, NFKBIA, ZNF770, HOXA6, SREBF1, HOXD9, ZZZ3, ZNF436, EPAS1, UBTF, CDC5L, ZNF250, FOXO3, KLF10, JDP2, ETV6, CEBPB, ZSCAN20, ZNF341, LHX2, JUN, KDM3A, BACH2, AR, ZNF133, ZNF33A, PAX5, MAFF, FOSL1, ZNF600, SRF, ARID1A, SATB1, ZNF383, TP63, HDAC6, RFX2, ZNF224, ZNF304, MYB.

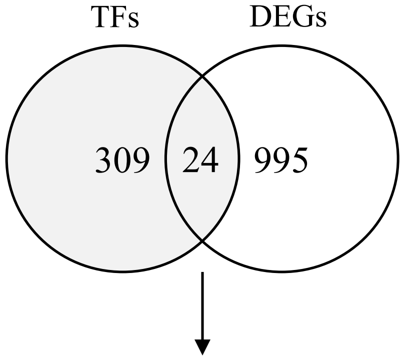

Common TFs and DEGs:

EP300
MAX
SMC3
NELFE
REST
SMARCB1
ETS2
KLF4
BDP1
ARID2
ARNTL
TCF7L1
FOXP2
SMAD2
CHD2
CREB1
ZNF449
RELA
HIF1A
TARDBP
XBP1
RAD21
PBX2
MEF2C

Supplementary figure 4. The list of identified upstream transcription factors (TF) regulating transcriptional activity of differentially expressed genes (DEGs) in the myometrium of restricted-diet-fed gilts. Results obtained with transcription factor enrichment analysis (TEFA) using TFEA.CHiP tool, and further used to prepare VENN diagram with the list of DEGs for further evaluation of common TFs and DEGs.
