# Supplementary material for: Nutritional restriction during the peri-conceptional period alters the myometrial transcriptome during the peri-implantation period
Source: Sci Rep. 2021 Oct 27;11:21187. doi: 10.1038/s41598-021-00533-x (PMC8551329; doi:10.1038/s41598-021-00533-x)
Supplement: Supplementary file 5 — Supplementary Table 1. [file 41598_2021_533_MOESM5_ESM.pdf]

Nutritional restriction during the peri-conceptional period alters the myometrial transcriptome during the peri-implantation period

Ewa Monika Drzewiecka, Wiktoria Kozlowska, Agata Zmijewska, Anita Franczak*

Affiliation: Department of Animal Anatomy and Physiology, University of Warmia and Mazury in Olsztyn, Oczapowskiego 1A, 10-719 Olsztyn, Poland

*Corresponding Author: Anita Franczak, Department of Anatomy and Animal Physiology, Faculty of Biology and Biotechnology, University of Warmia and Mazury
in Olsztyn, Oczapowskiego 1A, 10-719 Olsztyn, Poland; e-mail: anitaf@uwm.edu.pl

Supplementary table 1. The list of genes with altered expression in the myometrium of pigs during the peri-implantation period that were fed a restrictive diet during the peri-conceptional period comparing to the myometrium of pigs during the peri-implantation period that were fed a normal diet during the peri-conceptional period. DEGs not possessing DAVID ID are presented in red. FC (abs) – fold change absolute value.

P-value	FC (abs)	Official gene symbol	Accession No. to reference sequence in GenBank	Direction of regulation
0.01653065	5.71	NMB	NM_001123145	up
0.04747134	4.75	RBP4	XM_021072103	up
0.01679052	4.60	UFBP-2	NM_213845	up
0.04731457	4.31	WNT2	XM_003134756	up
0.01802151	4.14	HTR1B	XM_005659424	up
0.02969932	4.07	UABP-2	NM_213845	up
0.04464868	3.87	ACTA1	XM_005670976	up
0.02330657	3.56	SAA3	XM_013994503	up
0.02747832	3.16	FXYD3	NM_214208	up
0.00449847	3.07	NPY	NM_001256367	up
0.01936780	3.04	EPC1	AK348170	up
0.04365449	3.02	SLC16A1	NM_001128445	up
0.02760882	2.95	SRPX	XM_001927105	up
0.04386596	2.90	GPX3	NM_001115155	up
0.04180541	2.84	SCNN1A	XM_005653156	up
0.00031340	2.73	LOC100627162	AK390636	up
0.01031133	2.68	SRXN1	XM_003134361	up
0.01035481	2.66	CLU	NM_213971	up
0.03500071	2.65	RPN2	XM_021077220	up
0.03279758	2.65	C10H1orf106	XM_021064503.1	up
0.01261727	2.52	RAB37	XM_003131227.6	up
0.01132477	2.51	ABCB7	XM_021080712	up
0.02095707	2.49	PCCB	NM_213901	up
0.01710193	2.48	PSAT1	XM_021065150.1	up
0.03140422	2.48	CAT	NM_214301	up
0.00580595	2.47	AWN	XM_021073582	up
0.04843258	2.44	HUWE1	XM_003135102	up
0.03819681	2.43	PGRMC1	NM_213911	up
0.03987657	2.41	CWF19L1	XM_001929531	up
0.03910799	2.41	PTDSS1	XM_005662955	up
0.03145105	2.39	RNASE1	NM_001167655	up
0.04658715	2.39	NTRK3	XM_021083220.1	up
0.02581591	2.38	XKR8	XM_003127736	up
0.00025094	2.38	ENAH	XM_021064410.1	up
0.02207899	2.35	OAS2	NM_001031796	up
0.02580631	2.33	SNX5	XM_013985097	up
0.03502607	2.33	SLC25A32	XM_021089029	up

0.02064638	2.32	CPNE7	XM_021096150	up
0.03182578	2.32	CYR61	XM_001927740	up
0.00769782	2.32	HMGCR	NM_001122988	up
0.01089640	2.32	OPHN1	XM_021080304	up
0.01606256	2.31	PRKAB1	NM_001243621	up
0.00433924	2.30	PYGO1	XM_005654441	up
0.04667023	2.27	REEP2	XM_021084838	up
0.00529881	2.26	ADSSL1	NM_001097509	up
0.02242403	2.26	DAZAP2	XM_005655570	up
0.03200379	2.26	KLHL28	XM_005659918	up
0.01107241	2.26	PGM1	NM_001246318	up
0.02896533	2.25	GRN	NM_001044578	up
0.03594638	2.25	LUM	NM_001243339	up
0.00899816	2.23	TAB3	XM_021080831	up
0.03250636	2.22	CYP39A1	NM_001101027	up
0.01426525	2.21	PRIM2	NM_001244164	up
0.03210279	2.21	M6PR	XM_001927611	up
0.00284882	2.21	GAS7	XM_013981322	up
0.01141009	2.21	SLC35E4	XM_001929325	up
0.01237574	2.20	KIFC1	XM_021098594	up
0.00967423	2.20	S100A2	XM_001929556	up
0.03105305	2.20	RAB11B	NM_001244875	up
0.03737491	2.19	INSIG1	NM_001244521	up
0.02602377	2.19	KPNA1	NM_001163405	up
0.01489202	2.19	VCAN	NM_001206429	up
0.04432091	2.19	MFSD14A	XM_005663651	up
0.02694740	2.18	CSRNP1	XM_003132123	up
0.02444311	2.18	LOC110262034	XM_021100862.1	up
0.00516422	2.17	LIPA	NM_001123134	up
0.01387307	2.17	ANAPC7	XM_021072834	up
0.01047833	2.16	COL6A2	XM_005657193	up
0.02372934	2.16	HADHA	NM_213962	up
0.01804140	2.14	MICAL2	XM_007102646.2	up
0.00718542	2.13	PPT1	XM_003356331	up
0.04335012	2.12	LOC100156879	XM_005669522	up
0.00209217	2.11	CDV3	XM_021069464.1	up
0.00857424	2.11	C1H6orf120	XM_003121080	up
0.01192046	2.11	NPC1	NM_214322	up
0.01477035	2.11	CD164	XM_001924626	up
0.00606309	2.11	HADHB	NM_001348972	up
0.03469827	2.10	WDR11	XM_001924356	up
0.03393171	2.10	TPBG	XM_021091963	up
0.02721440	2.10	NDUFS2	XM_005663166	up
0.01222647	2.09	KDSR	XM_001925913	up

0.02328734	2.09	RHOB	XM_021085548	up
0.00226380	2.08	EMC9	XM_001928271	up
0.03133396	2.08	ERGIC3	XM_001929422	up
0.01386116	2.08	FAM136A	XM_003125051	up
0.04401817	2.08	CTDP1	XM_005660586	up
0.04979463	2.08	YIPF5	NM_001077225	up
0.00250650	2.08	FAM213A	XM_005671139	up
0.00257966	2.07	ATP2C1	XM_013990075	up
0.03513262	2.07	GJA1	NM_001244212	up
0.00413788	2.07	IFI30	NM_001131046	up
0.02030614	2.07	TFB1M	NM_001128475	up
0.00469850	2.07	TAP1	XM_021098370	up
0.04181223	2.06	TAX1BP3	XM_003131015	up
0.03971191	2.05	MCL1	NM_001348806	up
0.03655705	2.04	TADA3	NM_001206536	up
0.00834365	2.03	HADH	NM_214331	up
0.02295809	2.03	STK16	XM_013984521	up
0.02917219	2.03	TNIP1	XM_005672607	up
0.03597111	2.03	AKAP13	XM_003356732	up
0.02436615	2.03	NEK4	XM_021068929	up
0.01179176	2.02	BICC1	XM_021072797	up
0.03866046	2.02	GADD45A	NM_001044599	up
0.00834995	2.02	MINDY1	XM_001929665	up
0.00673662	2.01	ETS2	XM_001928469	up
0.00135096	2.01	PSMD1	XM_001925902	up
0.03341181	2.01	P2RY6	NM_001244296	up
0.04120283	2.01	CTSC	XM_013979241	up
0.02689539	2.00	FKBP4	NM_001195337	up
0.04849054	2.00	VANGL1	XM_001929540	up
0.01963740	2.00	ACP5	XM_021081258	up
0.03882030	1.99	SDR39U1	XM_001926728	up
0.00414757	1.98	CPT1B	NM_001007191	up
0.01117624	1.98	EEF1B2	NM_001243524	up
0.04457254	1.98	FABP3	NM_001099931	up
0.01851182	1.98	TBL1X	XR_002340726	up
0.01017484	1.97	CERS5	XM_021091642	up
0.00090645	1.97	NCEH1	NM_001243484	up
0.03586003	1.97	SRC	XM_021077969	up
0.00054406	1.97	PSME1	NM_214304	up
0.03441553	1.97	PRKAR1A	XM_005656932	up
0.02305041	1.97	CD109	XM_021092387	up
0.00686897	1.97	CYB5A	NM_001001770	up
0.04750856	1.96	DHCR7	XM_021082642	up
0.03029452	1.96	MRPS12	XR_002344650	up

0.03979189	1.96	BCKDHB	XM_021082900	up
0.00303983	1.96	CD40	NM_214194	up
0.00270345	1.96	NCOA4	NM_001243122	up
0.00532028	1.96	GNPAT	NM_001185140	up
0.03149219	1.96	SLC16A9	XM_001926829	up
0.01633194	1.95	PSAP	XM_005671041	up
0.00179493	1.95	ANAPC5	XM_001929287	up
0.00856601	1.95	MSN	XM_013986219	up
0.01988077	1.95	ELMOD3	XM_013987883	up
0.02187420	1.94	ICAM3	NM_001145379	up
0.02805390	1.94	TCF21	XM_021087668	up
0.00576489	1.94	ANO10	XM_021071717	up
0.01589463	1.94	TMEM19	XM_003126376	up
0.01451262	1.93	DYRK2	XM_021091962	up
0.04172663	1.93	CTSB	NM_001097458	up
0.00962495	1.93	EIF4A1	NM_001100196	up
0.03528217	1.93	MCUR1	XM_003128183	up
0.00025820	1.93	C1QTNF6	NM_001142830	up
0.02904465	1.92	TUBA1B	DQ084490.1	up
0.02620406	1.92	DCPS	NM_213790	up
0.02176351	1.91	TRIOBP	XM_013997529	up
0.01248545	1.91	FDPS	XM_021088556	up
0.01041478	1.91	UST	XM_021086988	up
0.01986186	1.91	HSD17B8	NM_001130730	up
0.04037745	1.91	SLCO3A1	XM_005666296	up
0.04193383	1.91	AGPAT1	NM_001033008	up
0.02893604	1.91	FBN1	NM_001001771	up
0.04879940	1.90	PEX6	XM_013977919	up
0.04165541	1.90	ODF2	XR_002345817	up
0.01385598	1.90	COL14A1	XM_013996512	up
0.01906877	1.90	NHEJ1	XM_021074747	up
0.04370181	1.90	EIF3E	XM_021089027	up
0.04726122	1.90	COMP	XM_003123527	up
0.01785690	1.90	ISG20L2	XM_001924747	up
0.02528951	1.90	ARF1	NM_001160427	up
0.03720047	1.89	LOC100737961	XR_302876	up
0.01145534	1.89	PUM1	XM_021095767	up
0.01209081	1.89	ENPP2	XM_003125510	up
0.00508991	1.89	COQ10B	XM_001928774	up
0.02257368	1.89	RNLS	XM_005671259	up
0.02620078	1.88	SMG5	XM_021089680	up
0.00140885	1.88	METTL9	NM_001243367	up
0.02048609	1.88	GPI	NM_214330	up
0.01926465	1.88	AKT3	XM_021063994	up

0.03710631	1.88	SRPRB	XM_021069470	up
0.00982678	1.87	DCTD	XM_001925083	up
0.00763962	1.87	ATP2A2	XM_019950165	up
0.01195077	1.87	TLE3	XM_021100713	up
0.03277982	1.87	RAN	XM_021072701	up
0.02463406	1.86	LYPLA1	XM_021089369	up
0.02333426	1.86	MAGEF1	XM_003483283	up
0.00267382	1.86	KPNA2	XM_021066215	up
0.02340231	1.86	TCN2	NM_001244436	up
0.00910552	1.86	BTBD7	XM_013989219	up
0.04570853	1.86	SERPINE 3	XM_013980493.2	up
0.01484225	1.85	PLIN2	NM_214200	up
0.03306316	1.85	PRKAG1	NM_001001642	up
0.02100446	1.85	GBA	NM_001005730	up
0.02368300	1.85	CCDC80	XM_003132680	up
0.01641287	1.85	PIGL	XM_003132024	up
0.01304284	1.85	AMD1	XM_003121345	up
0.04027778	1.85	TMEM263	XM_021091567	up
0.01131539	1.84	RAE1	XM_00567303	up
0.00870691	1.84	PPIB	XM_021093880	up
0.01626504	1.84	RNF111	XM_013992927	up
0.01006618	1.84	SMG7	XM_021063791	up
0.02242010	1.84	CLIC1	XM_021098461	up
0.04788644	1.84	WHAMM	XM_001927315	up
0.03827439	1.84	LGALS3	NM_001097501	up
0.01188844	1.84	ARHGAP10	XM_021100661	up
0.01356621	1.83	OAT	NM_001185141	up
0.02664877	1.83	CTSF	XM_003122495	up
0.03518147	1.83	RFTN1	XM_021071357	up
0.03437862	1.83	LDB3	XM_005657425	up
0.01008770	1.83	TRIM21	NM_001163649	up
0.03456139	1.83	NCK2	NM_001137631	up
0.01913896	1.83	RAD21	XM_005662889	up
0.02709256	1.83	DDOST	NM_214188	up
0.03972125	1.83	HNRNPAB	XM_021076627	up
0.03043788	1.82	ST3GAL4	NM_213757	up
0.01175448	1.82	GBE1	XM_021070783	up
0.00376716	1.82	TYSND1	XM_001927343	up
0.04980139	1.82	PLS3	XM_001925936	up
0.04675045	1.81	GLI2	NM_001315712	up
0.03156008	1.81	GTF3C4	XM_001929441	up
0.01786260	1.81	TCFL5	XM_021078230	up
0.02484773	1.81	PPP1R3C	XM_005671280	up
0.02586299	1.81	ISG15	NM_001128469	up

0.04057466	1.81	ALDH2	NM_001044611	up
0.03452934	1.81	ADRA2B	NM_001037148	up
0.02199850	1.81	CDC37	XM_021076770	up
0.03709177	1.81	VIM	XM_005668106	up
0.03460920	1.81	JPT2	NM_001048070	up
0.00234060	1.81	NPC2	NM_214206	up
0.00742447	1.81	SQLE	NM_001101026	up
0.01595622	1.81	OTUB1	NM_001162403	up
0.00496533	1.81	TK1	XM_021066553	up
0.02203297	1.80	C1orf109	XM_021095972	up
0.03305223	1.80	NSD1	XM_003123667	up
0.03113134	1.80	CCDC6	XM_001929282	up
0.00940116	1.80	LOC100510930	XM_021074050	up
0.02441526	1.80	CLN5	XM_005668472	up
0.00192187	1.80	CDC42SE1	NM_001190193	up
0.00816004	1.80	DAAM1	XM_021102686	up
0.01189818	1.80	PSD3	XM_005672670	up
0.03028673	1.79	UGDH	XM_003356899	up
0.00308420	1.79	STX12	NM_001243442	up
0.02639604	1.79	JAK1	NM_214114	up
0.01359157	1.79	SAMM50	XM_021091403	up
0.03405986	1.79	CHD2	XM_013978269	up
0.00880786	1.78	MTX1	NM_001039750	up
0.01206771	1.78	SARAF	XM_021076342	up
0.01775697	1.78	TMEM106C	XM_003355635	up
0.04509360	1.78	IDH2	NM_001164007	up
0.01020626	1.78	SGMS1	NM_001097438	up
0.01236351	1.78	HDDC3	NM_001243409	up
0.00499900	1.78	POLR3H	XM_001929133	up
0.02051060	1.78	LGMN	XM_005656451	up
0.02786343	1.78	PLAU	NM_213945	up
0.02452672	1.77	SYNC	XM_003356291	up
0.01609808	1.77	RPS6KA3	XM_021080343	up
0.00126596	1.77	WDR37	NM_001244454	up
0.04054305	1.77	ACOX1	NM_001101028	up
0.01280098	1.77	GHITM	NM_001244383	up
0.00747500	1.77	ZFAND3	XM_021099853	up
0.02885839	1.77	DNPEP	NM_001185186	up
0.00507156	1.77	PREP	NM_001004050	up
0.02335373	1.77	RPIA	NM_001097495	up
0.01832507	1.77	CYB561	XM_005668676	up
0.01139719	1.77	ERRFI1	XM_021095275	up
0.01475353	1.77	ZCCHC2	XM_021099630	up
0.03362202	1.76	CSNK2B	NM_001145377	up

0.01083356	1.76	SLC25A5	XM_001927440	up
0.02639429	1.76	COL15A1	XM_021066594	up
0.01607062	1.76	DRG1	XM_001927253	up
0.04646462	1.76	TUBB2B	XM_021100120	up
0.04560610	1.76	PEPD	XM_005653273	up
0.00623696	1.76	CRYBG1	XM_003121329	up
0.02480195	1.76	SLC25A23	NM_001246246	up
0.01881417	1.76	NPW	NM_213786	up
0.00700798	1.75	PCNA	NM_001291925	up
0.00393988	1.75	SVBP	XM_021096913	up
0.02633662	1.75	UMPS	XM_021071253	up
0.02080271	1.75	GTF2E1	XM_021070303	up
0.02031387	1.75	SEPHS2	NM_001093735	up
0.02485971	1.75	EP400	XM_005670513	up
0.03198883	1.75	CD83	XM_021100200	up
0.01106594	1.74	HSPB6	XM_003127059	up
0.00448877	1.74	PPA1	XM_001925080	up
0.04887796	1.74	LOC102157709	XR_298258	up
0.02488157	1.74	FITM2	FJ393219	up
0.02891668	1.74	PMVK	NM_001168416	up
0.00646051	1.74	SMAD2	NM_001256148	up
0.02107790	1.74	RBM4B	NM_001123188	up
0.04623720	1.74	LCMT2	XM_003353380	up
0.02323399	1.74	UBE2E2	XM_021068634	up
0.03881225	1.74	ARPC2	NM_001190239	up
0.01926685	1.74	ATXN1	XM_021099779	up
0.02014543	1.74	ANXA11	XM_005671147	up
0.03190640	1.74	CHPF	XM_003359649	up
0.04600482	1.74	CLDN5	NM_001161636	up
0.01957860	1.74	IFRD1	NM_001007519	up
0.02319356	1.74	RTN4	XM_005662544	up
0.03045945	1.74	CKAP4	NM_001244631	up
0.00630047	1.73	GOLGA7	XM_003134222	up
0.04268224	1.73	CTSD	NM_001037721	up
0.01476645	1.73	FAM210B	NM_001113701	up
0.03016792	1.73	SEC61A2	XM_003130808	up
0.04012483	1.73	SNRPC	XM_005656224	up
0.01315159	1.73	TPM3	XM_021088729	up
0.02346708	1.73	CREB1	NM_001099929	up
0.04094294	1.73	MX1	NM_214061	up
0.00059989	1.73	PPP2R1B	XM_003357285	up
0.01907399	1.73	ARF4	NM_001048072	up
0.04542422	1.73	SRSF1	NM_001038007	up
0.03455247	1.73	C1QTNF3	NM_001145386	up

0.02961277	1.73	SEC31A	XM_021101857	up
0.01424847	1.73	NABP2	XM_005655605	up
0.01356402	1.73	GFM1	XM_003358643	up
0.02436442	1.73	SGPP1	XM_003353501	up
0.00480128	1.73	CASTOR1	XM_013983042	up
0.00043394	1.73	BIRC6	XM_021087656	up
0.03472393	1.72	APP	NM_214372	up
0.01357304	1.72	GNA11	NM_001044538	up
0.03668818	1.72	AZIN1	XM_021088631	up
0.03094588	1.72	CHST11	XM_021092627	up
0.02752811	1.72	KIF21A	XM_013997927	up
0.03029173	1.72	DAP3	XM_021089709	up
0.01191456	1.72	KBTBD7	XM_003130995	up
0.00006606	1.72	REPS1	XM_005659169	up
0.02697332	1.71	TEK	XM_021062658	up
0.04836630	1.71	KLHL22	XM_021072689	up
0.01021002	1.71	CTSH	NM_213929	up
0.01673924	1.71	DENND1A	XM_021074650	up
0.03350339	1.71	CDK4	NM_001123097	up
0.01303543	1.71	NTF3	NM_001123152	up
0.01297364	1.71	VPS13D	XM_021095374	up
0.04223376	1.71	ARPC1B	XR_002342134	up
0.04188979	1.71	GNAS	NM_214312	up
0.01082956	1.71	TANGO2	XM_021072785	up
0.02106026	1.71	GLUL	NM_213909	up
0.01998952	1.71	MED15	XM_021073412	up
0.01696742	1.71	BNIP3L	XM_001927592	up
0.01560020	1.71	TUB	XM_021062268	up
0.04265831	1.70	ATP5B	XM_001929410	up
0.03131721	1.70	XBP1	NM_001142836	up
0.03378540	1.70	AMFR	XM_021097092	up
0.04235399	1.70	SRRM3	XM_021086294	up
0.00307012	1.70	LOC100624785	XM_021100120	up
0.03764123	1.70	SLA-5	NM_001114056	up
0.03110832	1.70	SMPD2	XM_001925290	up
0.04775655	1.70	TMED3	NM_001204363	up
0.02581248	1.70	PI4KA	XM_013983179	up
0.01417769	1.70	SNX1	XM_021093910	up
0.04919684	1.70	EEF1A1	NM_001097418	up
0.01763579	1.70	GEMIN5	XM_005672598	up
0.03046426	1.70	ARHGEF25	XM_003126313	up
0.00803184	1.70	WSB2	XM_021073844	up
0.02246941	1.70	FMOD	XM_003130105	up
0.02165496	1.70	ARPC1A	NM_001134355	up

0.03774614	1.70	CERS4	NM_001167631	up
0.03249074	1.69	NSDHL	NM_001167636	up
0.00560755	1.69	ATP2B4	XM_021063194	up
0.00435310	1.69	PBX1	XM_003355078	up
0.01785459	1.69	SNU13	NM_001190227	up
0.00336926	1.69	GTF2I	XM_005661993	up
0.04223165	1.69	GNAZ	XM_021072293	up
0.00325501	1.69	TMX1	XM_001928249	up
0.03771942	1.69	CTNNB1	XM_013981492	up
0.03763621	1.69	CTSV	NM_213892	up
0.01808403	1.69	ATP5A1	NM_001185142	up
0.01021496	1.69	NCOA5	XM_001929147	up
0.00766838	1.69	VLDLR	NM_001199890	up
0.03001778	1.69	SGCE	NM_001144124	up
0.04438934	1.69	NUDT12	XM_021084591	up
0.00413356	1.69	ATG5	NM_001037152	up
0.00745105	1.69	IARS2	XM_005656783	up
0.04403960	1.69	TM9SF2	XM_021065854	up
0.00922158	1.69	VDAC3	NM_214364	up
0.04669615	1.68	KCNB1	NM_214218	up
0.01145053	1.68	AKR7A2	NM_001243822	up
0.04388061	1.68	FYN	NM_001080206	up
0.03103602	1.68	RELA	NM_001114281	up
0.00437234	1.68	PHF10	XM_021085631	up
0.01029913	1.68	HSPA5	XM_001927795	up
0.03786106	1.68	S100A16	NM_001190207	up
0.03302010	1.68	BAG3	XM_021073458	up
0.01204274	1.68	RFLNA	XM_001926661	up
0.03766259	1.68	LOC100154081	XM_003480559	up
0.02464873	1.68	DHX32	XM_005671562	up
0.01385477	1.68	DIABLO	NM_001190216	up
0.00918220	1.68	C1S	NM_001005349	up
0.03614532	1.68	ZDHHC7	XM_003126823	up
0.01930239	1.68	ICAM-1	NM_213816	up
0.00292883	1.68	METTL6	NM_001206446	up
0.01477148	1.68	GYS1	NM_001195508	up
0.01466383	1.68	DAP	XM_003134178	up
0.00536980	1.68	SIPA1L2	XM_021073822	up
0.02719717	1.68	TCP11L1	XM_021083207	up
0.02224154	1.68	CAPZB	XM_005656027	up
0.03546047	1.68	ZNF142	XM_021076477	up
0.03198006	1.68	LOC100154462	XM_005654547	up
0.00583877	1.68	SMYD4	XM_021067574	up
0.01156118	1.67	ANGPTL2	NM_001109946	up

0.01630792	1.67	ITPA	XM_005672791	up
0.01772383	1.67	MICU1	NM_001243605	up
0.02149572	1.67	PTPRA	NM_001244485	up
0.01640075	1.67	CPSF3	XM_003354926	up
0.00531754	1.67	GLUD1	NM_001244501	up
0.02128896	1.67	BMP1	XM_021072336	up
0.04861534	1.67	SLC41A3	XM_021098938	up
0.00179929	1.67	CEND1	NM_213866	up
0.04956488	1.67	EIF2B1	XM_005670583	up
0.01111301	1.67	DHRS4	NM_214019	up
0.04705015	1.67	MRPL35	XM_003124936	up
0.04374753	1.67	BAX	XM_003127290	up
0.00668665	1.67	BCL7B	XR_002342318	up
0.00587293	1.67	CYCS	NM_001129970	up
0.00976654	1.67	PMM1	NM_001184895	up
0.03482761	1.67	ORMDL1	XM_001926349	up
0.03393159	1.67	CLIP3	XM_021097158	up
0.01253464	1.67	IPO5	NM_001244301	up
0.03520128	1.66	LOC106509348	XR_002345291	up
0.01840568	1.66	MAP2K1	NM_001143716	up
0.00959501	1.66	IDH1	XM_005672137	up
0.00432467	1.66	TMED10	XM_001926711	up
0.01915130	1.66	SSR2	XM_001928470	up
0.01627720	1.66	APEX2	XM_003135109	up
0.00543537	1.66	BZW1	XM_021074514	up
0.02683628	1.66	SLC6A7	XM_003124097	up
0.00591446	1.66	KIF3B	XM_021077423	up
0.04480267	1.66	PI4KB	XM_013997156	up
0.04070693	1.66	NFS1	NM_001136510	up
0.03416586	1.65	HSPCB	XM_005666063	up
0.03862722	1.65	LOC100623720	XM_021071094	up
0.02876986	1.65	LTBP2	XM_019427152	up
0.02199343	1.65	COPS4	XM_021100266	up
0.03172819	1.65	TPM1	XM_005659531	up
0.00171795	1.65	APTX	NM_213734	up
0.01771571	1.65	VDAC2	NM_214369	up
0.00664064	1.65	EIF1B	NM_001001635	up
0.01461974	1.65	THTPA	XM_001928723	up
0.00201514	1.65	HIF1A	NM_001123124	up
0.02096085	1.65	TSPAN15	XM_021072331	up
0.00830037	1.65	RBMX	XM_021079411	up
0.02284424	1.65	LYSMD1	NM_001243501	up
0.00621666	1.65	RPN1	NM_214333	up
0.01497626	1.64	PRUNE	XM_005663485	up

0.01905938	1.64	TUBB	NM_001044612	up
0.03877768	1.64	CDK5	XM_013990728	up
0.04882228	1.64	INTS9	XM_001928998	up
0.00984867	1.64	PSMC1	XM_021098047	up
0.01436110	1.64	DLGAP4	XM_021077873	up
0.03487508	1.64	CFI	XM_013989347	up
0.04139481	1.64	PKIG	XM_005672949	up
0.01914590	1.64	LOC100049695	NM_001098598	up
0.01198372	1.64	PROM1	XM_013978534	up
0.00517681	1.64	GATC	XM_001928504	up
0.04762867	1.64	GDF6	XM_021089078	up
0.02913529	1.64	CD302	NM_001110425	up
0.00683666	1.64	BLOC1S6	NM_001098592	up
0.00358971	1.64	MSMO1	NM_213752	up
0.04582734	1.64	IGFBP7	NM_001163801	up
0.04345732	1.64	CS	XM_021091143	up
0.00760557	1.63	USP10	XM_021093724	up
0.02984862	1.63	GCK	XM_003134883	up
0.00194939	1.63	IGBP1	XM_001927370	up
0.00660448	1.63	CPNE3	XM_021089150	up
0.04015167	1.63	PXK	XM_003132276	up
0.04669501	1.63	HSPA1L	NM_001123128	up
0.02277672	1.63	CMTM6	XM_021071540	up
0.00530762	1.63	SSFA2	XM_021076136	up
0.00680487	1.63	EIF2S1	XM_001928339	up
0.03761942	1.63	RPS19	AK346931.1	up
0.00929240	1.63	STN1	NM_001243685	up
0.01388640	1.63	PDPK1	XM_021086751	up
0.02492722	1.63	PRDX6	NM_214408	up
0.00889364	1.63	LGALS1	NM_001001867	up
0.00970963	1.63	C7H15orf40	XM_021088503	up
0.01630308	1.62	ACVR1	XM_021076448	up
0.02996956	1.62	GBP1	NM_001128473	up
0.03333515	1.62	FHL3	XM_013999158	up
0.03381052	1.62	CRYZ	XM_021093604	up
0.00442977	1.62	LDHB	XM_013988347	up
0.00282128	1.62	BID	NM_001030535	up
0.02916238	1.62	PDXK	NM_213943	up
0.04576809	1.62	NDUFAF4	XM_001925477	up
0.01495861	1.62	CDS2	NM_001166673	up
0.01747736	1.62	MYL6	XM_005655587	up
0.02179586	1.62	SYAP1	XM_005673457	up
0.03990838	1.62	PTTG1	XM_005672567	up
0.02251321	1.62	SLC25A3	NM_001164512	up

0.04273387	1.62	LOC100512544	XM_003128317	up
0.00651454	1.62	IKBKB	XM_021077171	up
0.02116880	1.62	CD59	NM_214170	up
0.01874930	1.62	ZBTB22	XM_001927786	up
0.01710328	1.62	ARV1	NM_001243293	up
0.04587625	1.62	CARM1	XM_021083821	up
0.03241915	1.62	FLOT1	NM_001128483	up
0.02505676	1.62	ANXA2	NM_001005726	up
0.00278243	1.62	NISCH	XM_005669637	up
0.02906793	1.62	BDKRB2	XM_005666427	up
0.00841536	1.61	ABCB8	XM_003360079	up
0.04233546	1.61	ATF4	XM_021090887	up
0.02784953	1.61	PPP2CA	NM_214366	up
0.00671236	1.61	LOC100623373	XM_005658604	up
0.01723367	1.61	SDHB	NM_001104953	up
0.01241149	1.61	YWHAH	XM_021073458	up
0.01908201	1.61	BIRC5	NM_214141	up
0.01856431	1.61	ABLIM1	XM_005671471	up
0.00506295	1.61	UBE2J1	NM_001077219	up
0.04418927	1.61	NELFCD	NM_001113698	up
0.00599637	1.61	OTULIN	XM_003133841	up
0.03313640	1.61	NELFE	XM_005665804	up
0.03434200	1.61	LOC100512568	XM_003134071	up
0.00335397	1.61	LRRC8C	XM_013997403	up
0.01606549	1.61	TCP11	XM_021098619	up
0.01144386	1.61	RPL10	NM_001044543	up
0.04346659	1.61	DLAT	NM_213994	up
0.01854926	1.61	TRIM13	XM_021065576	up
0.00506970	1.61	VDAC1P5	NM_214364	up
0.00368892	1.61	STMN1	XM_005665112	up
0.01317798	1.61	LOC110257186	XM_021076352	up
0.04503501	1.61	SELENON	XM_021095600.1	up
0.02630963	1.61	RFTN2	XM_021075725	up
0.02964386	1.61	PKM	XM_021099106	up
0.03014388	1.60	PTPRS	XM_021084028	up
0.04732491	1.60	PEA15	XM_001928960	up
0.03840095	1.60	SLC35B2	XM_003128416	up
0.00301304	1.60	ABCF1	NM_001123069	up
0.04428043	1.60	TSPAN3	NM_001244196	up
0.00295689	1.60	COQ10A	XM_003126267	up
0.02461095	1.60	LRRC8D	XM_005663697	up
0.02784655	1.60	MFSD3	XM_021090517	up
0.02515126	1.60	TMEM54	XM_005665194	up
0.01603790	1.60	ARHGEF12	XM_021062982	up

0.02790098	1.60	PNPLA2	NM_001098605	up
0.01749353	1.60	N4BP2L1	XM_021065183	up
0.00033028	1.60	MDH2	NM_001244153	up
0.01823026	1.60	SELENOF	NM_001085443	up
0.01813452	1.60	ARNTL	NM_001097425	up
0.04939355	1.60	PAPOLA	XM_001929075	up
0.04695357	1.60	GLMP	XM_001927751	up
0.02447667	1.59	FAM92A	XM_013996613	up
0.00165579	1.59	ATP6V1A	NM_001004042	up
0.01923274	1.59	ASCC1	NM_001244084	up
0.02814136	1.59	FZR1	NM_001012296	up
0.03020626	1.59	RAB27B	XM_001925811	up
0.04015468	1.59	FUNDC2	NM_213743	up
0.02262708	1.59	C-FLIP	NM_001001628	up
0.02219082	1.59	MRPL18	XM_003121109	up
0.01084868	1.59	RXRB	NM_001190246	up
0.01025437	1.59	SNX18	XM_021076956	up
0.04475223	1.59	RPP30	XM_001924771	up
0.00727636	1.59	RHOA	XM_021068521	up
0.04118036	1.59	KIAA1324	XM_021090086	up
0.00402845	1.59	NUB1	XM_013990737	up
0.00432483	1.59	EIF6	XM_021077274	up
0.00052192	1.59	C1H9orf16	XM_001927625	up
0.03825677	1.59	SLC35B4	XM_013985613	up
0.01175153	1.59	LOC100037956	NM_001097453	up
0.03614540	1.59	GSTZ1	NM_001243638	up
0.00723371	1.59	FBLN5	XM_021099623	up
0.00190389	1.59	ATP5F1	XM_003355244	up
0.00673446	1.58	SKIP	XM_005657014	up
0.01533397	1.58	LOC100153598	XM_021068950	up
0.02890574	1.58	SUPT3H	XM_021099856	up
0.04382197	1.58	PHKA1	XM_021080691	up
0.02263105	1.58	FAT1	NM_001190170	up
0.01004325	1.58	PSMD10	XM_001926907	up
0.02232006	1.58	RPLP0	NM_001098598	up
0.03605666	1.58	ABCG2	NM_214010	up
0.03088962	1.58	MED23	XM_021087970	up
0.03754856	1.58	DGCR8	NM_001206919	up
0.01853843	1.58	SRSF2	XM_021066262	up
0.02496287	1.58	FUCA1	NM_001315628	up
0.00056526	1.58	APEX1	XM_021098038	up
0.00097472	1.58	KLHL29	XM_021087786	up
0.00955790	1.57	TRPM7	XM_013993003	up
0.01748903	1.57	TIGD2	XM_021101762	up

0.02990839	1.57	PECAM1	XM_013980837	up
0.00923126	1.57	SAR1B	NM_001008689	up
0.01817289	1.57	FTHL17	XM_001924691	up
0.04037288	1.57	C2H19orf25	XM_003353978	up
0.00823130	1.57	CLDND1	NM_001243498	up
0.00889516	1.57	MTFP1	XM_001929262	up
0.02522422	1.57	LOC100154105	XM_001924950	up
0.02457697	1.57	PSMB5	XM_001924445	up
0.02832799	1.57	SPATA6L	XM_021064005	up
0.04946373	1.57	ASCC3	XM_021090356	up
0.03100269	1.57	DNASE1L1	NM_001038632	up
0.01604403	1.57	KCNJ8	XM_003126434	up
0.01105954	1.57	UFC1	XM_003125654	up
0.01047175	1.57	FAM45A	XM_005671503	up
0.00968716	1.57	ATE1	XM_021073564	up
0.02084702	1.56	TRAF4	XM_021067485	up
0.01297704	1.56	SLC30A6	NM_001137623	up
0.02382580	1.56	MINPP1	XM_001927672	up
0.02395946	1.56	ITGA5	XM_001925252	up
0.02711489	1.56	SLC1A5	XM_003127238	up
0.00352335	1.56	ME1	XM_001924333	up
0.01762949	1.56	MRPL24	XM_005663257	up
0.01490432	1.56	WSCD2	XM_021072411	up
0.00932959	1.56	EIF4A3	NM_001100193	up
0.00515821	1.56	CDK5RAP2	XM_021068104	up
0.00725185	1.56	TCF7L1	XM_021087302	up
0.00159163	1.56	WTAP	XM_005659114	up
0.01032050	1.56	HILPDA	NM_001348757	up
0.01219733	1.56	MESP2	XM_001926032	up
0.02452144	1.56	ANAPC16	XM_013983316	up
0.01720274	1.56	PDCL	XM_001927696	up
0.03082686	1.56	RPL8	XM_005655297	up
0.04312719	1.55	REEP3	XM_001925659	up
0.04737593	1.55	PIK3R4	XM_021071332	up
0.04690996	1.55	HSD11B1	XM_021102234	up
0.01680774	1.55	VPS33A	NM_001098604	up
0.04618819	1.55	P4HA2	XM_003354272	up
0.01367745	1.55	PARP6	XM_021099109	up
0.00953781	1.55	EFTUD2	XM_003131332	up
0.00402466	1.55	EDEM1	XM_021069285	up
0.00349981	1.55	NUDT18	XM_001927480	up
0.00610033	1.55	PRDM10	XM_013979567	up
0.03202146	1.55	GNAQ	NM_001038623	up
0.04102613	1.55	CLIP2	XM_021086339	up

0.01325292	1.55	XPO7	XM_013990257	up
0.00489644	1.55	ABHD2	XM_001925989	up
0.01142366	1.55	SNRPD3	NM_001243426	up
0.00607438	1.55	FOXP1	XM_021070838	up
0.02452426	1.55	CHMP7	XM_021074113	up
0.00601292	1.55	NSMCE4A	XM_001925705	up
0.00878565	1.54	IFNGR1	NM_001177907	up
0.02253077	1.54	SYNE1	XM_021084410	up
0.03813220	1.54	CD9	NM_214006	up
0.01625883	1.54	PLEKHO2	XM_013992891	up
0.00246317	1.54	LCTHIO	NM_213966	up
0.02440871	1.54	SLC35A5	XM_013982457	up
0.04365034	1.54	PRKD1	XM_021099186	up
0.00869096	1.54	SH3D19	XM_005666815	up
0.02623926	1.54	HEXA	XM_021097913	up
0.03390108	1.54	SMARCB1	XM_001929438	up
0.04623537	1.54	AVL9	XM_021079002	up
0.00850619	1.54	VPS72	XM_001929654	up
0.04838653	1.54	AIFM2	XM_005657401	up
0.04220573	1.54	SDF2L1	XM_001929542	up
0.02170989	1.54	EMC3	XM_003358517	up
0.01437517	1.54	ACLY	NM_001257276	up
0.01323004	1.54	TMEM59	NM_001044558	up
0.00460097	1.54	RPS4X	NM_001204283	up
0.01721721	1.54	CUTC	XM_021072322	up
0.02144109	1.54	C1H9orf40	XM_003121960	up
0.01408260	1.54	PPP3CB	XM_005671106	up
0.02789139	1.54	KLF4	NM_001031782	up
0.02835629	1.54	TSPYL1	XM_003121374	up
0.01215945	1.53	MKL2	XM_021086566	up
0.04104296	1.53	RDH10	XM_021089230	up
0.00610425	1.53	DDT	NM_001243557	up
0.03598676	1.53	IGFBP6	NM_001100190	up
0.01053499	1.53	TMEM63B	XM_021098785	up
0.01544832	1.53	ALPK3	XM_021098900	up
0.00070068	1.53	MAPRE1	NM_001044613	up
0.01570147	1.53	VTI1A	XM_013983738	up
0.00773231	1.53	CLSTN1	XM_021095293	up
0.03591858	1.53	AXIN2	XM_021066733	up
0.00721840	1.53	NFKBIL1	XM_001928795	up
0.03383744	1.53	BTG3	NM_001097517	up
0.04601324	1.53	LOC102157934	XR_309220	up
0.00619763	1.53	SPTLC2	XM_021099554	up
0.02836828	1.53	MYO6	NM_214021	up

0.00141233	1.53	LRRC28	XM_001925096	up
0.02394619	1.53	NCBP1	XM_021066300	up
0.03187364	1.53	LRPAP1	NM_001113436	up
0.04348531	1.53	FTH1	XM_005660803	up
0.01927988	1.53	PTI	NM_001190215	up
0.04630212	1.53	KDM5C	NM_001097433	up
0.02141086	1.53	PIK3CB	XM_021069525	up
0.01042690	1.53	PPIH	XM_003128099	up
0.01134527	1.53	GMPR2	XM_005666235	up
0.02879872	1.53	UROS	NM_001244366	up
0.03450666	1.53	DYNC1H1	XM_021081584	up
0.00796222	1.53	CRY1	XM_003126079	up
0.00699992	1.53	ARL6IP5	NM_001048073	up
0.02424234	1.52	SNX29	XM_021088055	up
0.00140087	1.52	ALG5	NM_001244320	up
0.03324251	1.52	PRDX3	NM_001244531	up
0.03071524	1.52	LOC100152299	XM_013997120	up
0.00966961	1.52	LOC100737714	XM_003481479	up
0.00559355	1.52	PSTPIP1	NM_001244186	up
0.04078817	1.52	RAB5C	NM_001244254	up
0.04239862	1.52	MMP2	NM_214192	up
0.04033751	1.52	PRUNE1	XM_005663485	up
0.03667842	1.52	LAMB1	XM_003130269	up
0.02588668	1.52	CDK5RAP1	XM_001928856	up
0.02897597	1.52	DNAJC17	XM_001929383	up
0.01583312	1.52	SRP54	XM_001927799.	up
0.02979093	1.52	BSG	NM_001123086	up
0.01868906	1.52	JAGN1	XM_021069312	up
0.03840381	1.51	ARF5	NM_001145221	up
0.01741509	1.51	MIC-2	NM_001114274	up
0.01739240	1.51	DHX58	NM_001199132	up
0.04894278	1.51	COPZ1	XM_001927549	up
0.01333787	1.51	LOC100152229	XM_003483541	up
0.03623117	1.51	ABCD4	XM_001929196	up
0.04064675	1.51	ETFA	NM_001244403	up
0.00345148	1.51	VPS8	XM_021069974	up
0.01064350	1.51	RIT1	NM_001077230	up
0.03790043	1.51	RRBP1	XM_021078036	up
0.03004176	1.51	PPP1R10	NM_001123165	up
0.03783520	1.51	CFLAR	NM_001001628	up
0.03221491	1.51	NIFK	NM_001101827	up
0.00311600	1.51	MOBKL3	NM_001032379	up
0.02058213	1.51	PDE10A	XM_021071805	up
0.02332999	1.51	SOD2	XM_005659113	up

0.04980572	1.51	GOLPH3L	XM_013997188	up
0.04359378	1.51	GOLGA3	XM_021073867	up
0.01493620	1.51	CAPN2	NM_001100188	up
0.00872848	1.51	FTL	NM_001244131	up
0.04680139	1.50	TXNDC5	XM_021100160	up
0.03178544	1.50	RBKS	XM_021085801	up
0.01405361	1.50	SPCS1	NM_001114288	up
0.01439533	1.50	GSTA4	NM_001243379	up
0.00936389	1.50	COPS5	XM_013996694	up
0.02813433	1.50	KCTD20	XM_005665922	up
0.04496378	1.50	RAI2	XM_021080586	up
0.00848236	4.90	PELO	XM_013984761	down
0.01282775	4.80	FOXP2	XM_021078435	down
0.00351351	4.55	SLC4A7	XM_021071476	down
0.02605828	4.50	IRF2BP2	XM_021074288	down
0.00014711	4.31	FZD1	XM_021102361	down
0.00164190	4.24	PHACTR2	XM_021087164	down
0.00472723	3.78	LOC100621006	XM_021101377	down
0.01119047	3.62	LOC106509575	XR_001307225.2	down
0.00337159	3.59	HNRNPLL	XM_019942803	down
0.02503123	3.57	REST	XM_005666690	down
0.01500155	3.56	HOOK3	XM_021077850	down
0.00461170	3.52	DDX39B	XR_002345591	down
0.01241197	3.49	MEF2C	XM_021082279	down
0.02681823	3.49	ZFHX4	XM_021089177	down
0.00503120	3.34	ZBTB44	XM_003130079	down
0.00003437	3.33	PIFO	XM_005663561	down
0.03423424	3.17	C5H12orf75	XM_021091585	down
0.01993566	3.12	MTPN	XM_021078487	down
0.00234548	3.09	CAP2	XM_021100214	down
0.01394958	2.96	AM20A	XM_005656933	down
0.01822371	2.95	C8H4orf19	XM_021101098	down
0.01326804	2.90	B3GLCT	XM_021065554	down
0.02530786	2.88	PTPN11	XM_005657333	down
0.02056326	2.85	DLG3	XM_013990919	down
0.02508871	2.82	PAFAH1B2	XM_021062879	down
0.00768675	2.78	LOC110256222	XR_002337506.1	down
0.00149810	2.76	AP4S1	XM_021098036	down
0.00814379	2.76	BCHE	XM_003358664	down
0.04895402	2.74	NTRK2	AK239612	down
0.03450663	2.72	NF1	XM_021067460	down
0.00594612	2.72	RNPC3	XM_005663632	down
0.00251369	2.72	MGEA5	XM_021072730	down
0.01692518	2.68	CABYR	NM_001348766	down

0.01682588	2.68	SYNM	XM_021098348	down
0.00104882	2.67	HNRNPA3	XM_021075138	down
0.02672016	2.66	C1H15orf41	XM_021098122	down
0.01479684	2.66	RYR2	XM_021072683	down
0.01429200	2.63	RBM14	XM_007171027	down
0.01194662	2.63	MAX	XM_021099386	down
0.01302050	2.59	LOC102163816	XR_002341443.1	down
0.00235580	2.59	TBC1D32	XM_021088942	down
0.00406446	2.55	LOC110259223	XR_002341394	down
0.01151793	2.53	LARP7	NM_001243815	down
0.00269307	2.52	CDO1	XR_002339836	down
0.00043704	2.51	AGBL3	XM_021079175	down
0.01360440	2.49	TROVE2	XR_002336171	down
0.01488131	2.49	CPSF6	XM_021090905	down
0.01612615	2.49	STAU2	XM_021089229	down
0.00437533	2.49	ACACB	EU853705	down
0.00590791	2.48	SEMA5A	XM_021076851	down
0.03077573	2.47	TSTD3	XM_013992718	down
0.01848252	2.43	IPO7	XM_021062248	down
0.01604589	2.42	CMAH	NM_001113015	down
0.02124481	2.42	TP53BP1	XM_006778026	down
0.03684359	2.41	AFF3	XM_021088196	down
0.02256746	2.40	PI15	XM_001926809	down
0.01332412	2.38	TARDBP	XR_002344897	down
0.03909987	2.37	LOC110260440	XR_002343742	down
0.00602291	2.36	ND6	AK399664	down
0.00151865	2.34	LOC102164547	XM_013998421	down
0.00109412	2.31	LYRM7	XM_013995089	down
0.00060322	2.31	STN1	XM_021072025	down
0.00662163	2.31	CCDC88A	XM_005662552	down
0.00108201	2.31	LOC110259328	XR_002341605	down
0.02548527	2.30	INTS7	XM_021063859	down
0.02713849	2.30	MAP2K5	XM_021100489	down
0.02211844	2.30	LOC102161650	XR_002346790	down
0.00042050	2.28	PNISR	NM_001113439	down
0.03364280	2.27	LSM5	XM_005673274	down
0.00498885	2.26	LOC100513233	XM_003131766	down
0.00327284	2.26	PDE4B	XM_021093174	down
0.04675448	2.26	ZDHHC20	XR_002336601	down
0.00577433	2.25	ACSM5	XM_021086523	down
0.00493103	2.23	LOC102164761	XR_002341205	down
0.00790962	2.23	SEMA6A	XM_005661593	down
0.02781423	2.22	LOC102162727	XR_301706	down
0.03791814	2.22	TCP11L2	XM_021091579	down

0.02821750	2.22	TATDN3	XM_021063851	down
0.03096062	2.21	ARL4A	XM_005667651	down
0.00068780	2.21	MBLAC2	XM_003123780	down
0.04810385	2.21	LOC110259298	XR_002341572	down
0.00985277	2.20	MED6	AK232455	down
0.03761204	2.18	RUFY2	XM_021073521	down
0.04580714	2.17	ARID2	XM_021092987	down
0.03309530	2.16	KCNE2	XM_021070947	down
0.00245748	2.15	THRB	XM_021071436	down
0.03208654	2.15	LOC106507939	XM_021065187	down
0.01554164	2.15	FPGT	AK230541	down
0.00324925	2.15	ARL14EP	XM_021083232	down
0.00216387	2.14	LOC110255653	XR_002336256	down
0.00524707	2.14	RHOU	XM_021072737	down
0.00068775	2.13	DEPTOR	XM_021090555	down
0.04719374	2.13	FNBP4	XM_021083063	down
0.03391394	2.13	LOC102165633	XR_001302653	down
0.02552580	2.13	YAP1	XM_021062706	down
0.03634808	2.12	GHR	XM_021076567	down
0.00172082	2.12	TMEM106B	XM_003130176	down
0.02292715	2.11	CHD9	XM_021094174	down
0.03080753	2.11	CNTLN	XM_001925321	down
0.00976740	2.11	DGKH	XM_021065624	down
0.04425075	2.11	C1D	XM_021087429	down
0.00010986	2.11	BAZ2B	XM_021074758	down
0.00160237	2.10	EP300	XM_001929213	down
0.04241690	2.09	CCDC91	NM_001244526	down
0.04716872	2.08	LOC100152572	AK232762	down
0.02886905	2.08	LOC110259371	XM_021084173	down
0.00300635	2.08	LOC110257362	XR_002339872	down
0.01064505	2.07	VGLL3	XM_005657128	down
0.00400737	2.06	RNF187	XM_021085058	down
0.01536636	2.06	AGO3	XM_021093291	down
0.02731966	2.06	CENPC	XM_003482404	down
0.00721005	2.05	SRSF10	XM_021093377	down
0.01901652	2.05	SCAPER	XM_021099012	down
0.00386628	2.05	LRP8	XM_021093237	down
0.00314071	2.04	INPP5K	XM_005657013	down
0.03858876	2.03	IFN-DELTA-9	NM_001164848	down
0.04614132	2.03	PBX2	XM_012436748	down
0.00287088	2.03	SIAH1	XM_021094233	down
0.00387064	2.02	LOC100515492	XR_116026	down
0.00451924	2.02	MOCS2	XM_021076548	down
0.01496694	2.02	ARHGAP5	XM_021099184	down

0.00347782	2.02	GTF2H3	AK352195	down
0.03424905	2.01	PTGFR	XM_005665359	down
0.03431298	2.01	ZNF449	XM_021080812	down
0.00230038	2.01	PYROXD1	XM_021092254	down
0.02023808	2.01	GYG2	XM_021079760	down
0.00827722	2.01	ATRX	XM_021080080	down
0.01327704	2.01	IGFBP-5	DQ320122.1	down
0.02022258	2.00	NR2F1	XM_003354214	down
0.02990106	2.00	EEA1	XM_005664267	down
0.04021874	2.00	AFF1	XM_005667021	down
0.01623188	2.00	ZCCHC6	XM_021064632	down
0.00246131	2.00	TARS2	XM_003355197	down
0.02124517	1.99	ARID4B	XM_021074399	down
0.00230372	1.99	UBXN7	XM_021070161	down
0.02682713	1.99	HOXA3	XM_021079038	down
0.00318368	1.98	KPNA5	XM_005654392	down
0.00413782	1.98	MYO15A	XM_021067996	down
0.01877076	1.98	CEP290	XM_021092777	down
0.01277156	1.98	PLAGL1	XM_021084039	down
0.02264313	1.98	KIAA1958	XM_003122070	down
0.00275056	1.97	TREM-1	KC688694	down
0.02612984	1.97	CALD1	XM_021079174	down
0.00637645	1.97	TTC14	XM_001927185	down
0.00904243	1.97	SNTB2	XM_021093874	down
0.00483497	1.95	CASP10	NM_001161640	down
0.00529719	1.95	PRKRIP1	XR_002342313.1	down
0.02019454	1.94	ZNF583	XM_003356077	down
0.00680092	1.94	RGN	XM_005673574.3	down
0.00604353	1.94	HIBCH	XM_021076265.1	down
0.00220173	1.94	CNTRL	XM_005660404.3	down
0.00301855	1.94	ZKSCAN7	XM_013990090.2	down
0.00127504	1.94	RYR3	XM_021099907.1	down
0.02081114	1.94	SPTLC1	XM_003483378.4	down
0.00447232	1.94	FGD6	XM_021092744.1	down
0.00569645	1.94	SLC2A11	XM_001929403.5	down
0.00668781	1.93	VAT1L	XM_003126850.6	down
0.00231053	1.93	VAPB	XM_021077209.1	down
0.00110834	1.93	TBC1D19	XM_013978566.2	down
0.00996174	1.93	B3GNT6	XM_021062552.1	down
0.00728145	1.92	CCDC18	XM_021090202.1	down
0.00760935	1.92	APOB	GU328987.1	down
0.03113919	1.92	DDX46	NM_001246268.1	down
0.00918953	1.91	SLC25A37	AK347227	down
0.00861735	1.90	OTUD4	XM_021100662.1	down

0.01757755	1.90	LOC100736666	XM_003482642	down
0.00568274	1.90	KANK1	XM_021064444.1	down
0.00170153	1.90	SYT14	XM_021063881.1	down
0.01210129	1.90	IFT140	XM_021086840.1	down
0.00730735	1.90	LOC100156775	XM_001925390	down
0.01642868	1.89	ND4	AK230497	down
0.00354236	1.88	KLF17	NM_001164010	down
0.00683590	1.88	MUT	NM_214405	down
0.00283451	1.88	C12H17orf62	XM_005656864	down
0.00600628	1.88	IFNAR2	NM_001204775	down
0.00446349	1.86	UPB1	XM_001929254	down
0.01468219	1.85	LOC100737724	XM_003483956	down
0.04311753	1.84	RPP40	XM_005653448.3	down
0.00008191	1.84	SFRS18	NM_001113439	down
0.00899176	1.84	POU2F1	NM_214264	down
0.00598830	1.83	SYTL3	AK348792	down
0.03230588	1.82	MORC3	AK230727	down
0.01613127	1.82	ZNF398	XM_003130281	down
0.01257454	1.81	SLC37A4	XM_005667399	down
0.02193053	1.80	SFRP5	NM_001243406	down
0.00418393	1.80	SMEK1	AK349358	down
0.01069471	1.80	FBXL6	XM_013996410.2	down
0.02198445	1.79	HOXA10	NM_001257354	down
0.00351326	1.79	AKAP9	XM_021102196	down
0.04439670	1.78	COX2	AK239715	down
0.00777848	1.77	LOC100624255	EW109540	down
0.01804493	1.77	POC5	XM_005661495	down
0.02087992	1.77	PIKFYVE	XM_005672138	down
0.04652216	1.77	LOC100519100	XM_003130320	down
0.01162408	1.76	ORC4	XM_005671605	down
0.01094393	1.76	GCC2	XM_003124853	down
0.01331719	1.76	ESF1	XM_001926068	down
0.00349016	1.76	SLC39A14	AK233452	down
0.01732961	1.76	ARID4A	NM_001244179	down
0.03761207	1.76	LOC100523347	XM_003121767	down
0.00435062	1.76	LIMS1	NM_001243858	down
0.01301004	1.76	MTF2	XM_005663668	down
0.01424564	1.76	SLC29A1	XM_005666066	down
0.01660000	1.75	SRSF11	NM_001044587	down
0.02713241	1.75	SRP19	EV877673	down
0.02293980	1.75	BDP1	XM_005672529	down
0.00280862	1.74	CNPY3	NM_001110428	down
0.01664833	1.74	PAXBP1	XM_003132753	down
0.03042495	1.73	GLO1	XM_001927957	down

0.03571654	1.73	RSRC2	XM_005670608	down
0.02344636	1.73	LOC595121	XM_005667648	down
0.00713121	1.72	ZNF385D	XM_003132072	down
0.01060090	1.72	ODF2L	XM_005663732	down
0.00932037	1.71	NEMF	XM_001924630	down
0.02860038	1.71	ND2	AK392713	down
0.02954480	1.70	ITGB1	NM_213968	down
0.03033269	1.70	TXNIP	NM_001044614	down
0.00231359	1.70	NFIB	XM_005660096	down
0.01307612	1.69	MAMDC2	AJ939033	down
0.00491063	1.69	DYNC1LI2	NM_001243620	down
0.04339942	1.69	CARP	NM_213922	down
0.00221030	1.68	SMEK2	NM_001244675	down
0.00784001	1.68	ALG10	NM_001244234	down
0.01113066	1.68	NFX1	AK238006	down
0.00649662	1.68	ADH4	NM_001315670.1	down
0.00372000	1.68	EML4	XM_005662588	down
0.00968324	1.67	MYO1E	AK236366	down
0.01875886	1.67	SMC3	XM_021944067	down
0.00027977	1.66	GTSF1	XM_005663861.3	down
0.00670942	1.66	PRPF40A	NM_001244573	down
0.02131991	1.66	TATDN1	NM_001244525	down
0.01825118	1.65	ZC3H15	XM_001926616	down
0.01290267	1.65	CPT1C	AK240307	down
0.01664216	1.65	NT5C2	XM_001929278	down
0.00931592	1.65	ERCC1	AK235974	down
0.02186345	1.64	MUC13	NM_001105293	down
0.02680880	1.64	RDX	NM_001009576	down
0.01540453	1.64	RBM25	XM_005666337	down
0.01404647	1.64	NBN	AK238840	down
0.00970031	1.64	SGMS1	AK232830	down
0.04189932	1.64	ZMYM4	AK348051	down
0.02646238	1.64	LOC100517869	XM_003484265	down
0.03612572	1.64	MARCH7	AK234676	down
0.00854055	1.63	LOC100155029	XM_001925131	down
0.03010587	1.63	MCM8	XM_005672680	down
0.03813059	1.63	NKTR	XM_005669412	down
0.01677343	1.63	ALG2	NM_001101029	down
0.03536935	1.63	RSC1A1	NM_213793	down
0.02647033	1.62	ECHDC1	AK239240	down
0.02352476	1.62	INSR	XM_021083943.1	down
0.01564992	1.62	CHD6	XM_003359996	down
0.01633149	1.62	PPIG	XM_005671916	down
0.04784935	1.62	DLL4	NM_001244418	down

0.03927726	1.62	IFN-DELTA-6	NM_001166312	down
0.00570764	1.61	CLIC5	NM_001198923	down
0.02657768	1.61	USP47	XM_021083316.1	down
0.04431658	1.61	LSAMP	XM_021068415.1	down
0.00943078	1.61	CD5	AK236857	down
0.04106136	1.61	PHLDB2	XM_005674193	down
0.01558257	1.61	WWP1	AK236329	down
0.01998025	1.60	BRD3	XM_003484312	down
0.02362402	1.60	C16H5orf42	XM_005672420	down
0.04599936	1.60	TRIP11	XM_001926825	down
0.02567118	1.60	TFAM	AK230853	down
0.03448790	1.60	THNSL1	XM_003357757	down
0.04329097	1.60	CD47	NM_213982	down
0.04200531	1.59	MICU3	XM_003359862	down
0.00115722	1.59	LRP6	XM_005664069	down
0.01705287	1.59	STOML3	XM_005668357	down
0.00797063	1.59	SNX16	XM_001926702	down
0.04576451	1.59	KLHL2	XM_003128989	down
0.01182613	1.58	CSPP1	XM_001926004	down
0.01124030	1.58	USPL1	XM_003482872	down
0.00195469	1.58	ZNF582	XM_003356090	down
0.00957740	1.57	CD86	XM_005654041	down
0.01908919	1.57	PCDH7	NM_001244484	down
0.00231190	1.57	IGFBP1	AB119126.1	down
0.00370088	1.57	DPY19L4	XM_001926300	down
0.00619432	1.57	PRPF39	XM_005659927	down
0.00787627	1.57	PNN	AK231056	down
0.00099244	1.56	FRA10AC1	NM_001243654	down
0.01913493	1.56	ZNF512	XM_005653047	down
0.02543709	1.56	ITGB3	NM_214002	down
0.01877575	1.56	ZBED5	XM_005661128	down
0.00832199	1.55	RYBP	XM_005669728	down
0.03268962	1.55	SLIRP	NM_001097472	down
0.01675746	1.55	COPS6	AK239715	down
0.02613898	1.55	LOC100515003	XM_003121947	down
0.01883921	1.54	TLK1	XM_001927890	down
0.00274739	1.54	EIF3A	XM_001928342	down
0.00011899	1.54	SLK	XM_001929430	down
0.02853332	1.54	PGRMC2	NM_001097521	down
0.01314070	1.54	NLN	NM_214359	down
0.04355487	1.54	RRM2B	XM_001925001	down
0.01798823	1.53	ZFP36L1	XM_005656350	down
0.02409717	1.53	AFTPH	XM_003481200	down
0.01845499	1.53	USP46	XM_021101210.1	down

0.03667368	1.53	CAPZA2	NM_001097455	down
0.03279248	1.53	SSB	XM_005671925	down
0.03575392	1.53	COL1A2	AK347299	down
0.02346055	1.52	MLLT3	XM_003121886	down
0.01907399	1.52	HAO2	XM_005655407	down
0.03160843	1.52	OGT	NM_001039748	down
0.02983392	1.51	ZNF318	AK345653	down
0.04049782	1.51	LOC733612	AK392314	down
0.00843292	1.51	SEC62	NM_001105305	down
0.03044625	1.51	SCYL2	XM_021092724.1	down
0.02784128	1.51	MICAL3	AK234676	down
0.00861206	1.51	ITGA1	XM_021076769.1	down
0.01420366	1.51	MPHOSPH10	XM_001927513	down
0.01793761	1.51	TOP2B	NM_001258386	down
0.01511350	1.50	TLN1	XM_003353567	down
0.00888534	1.50	GON4L	XM_005658357	down
