# Supplementary material for: Nutritional restriction during the peri-conceptional period alters the myometrial transcriptome during the peri-implantation period
Source: Sci Rep. 2021 Oct 27;11:21187. doi: 10.1038/s41598-021-00533-x (PMC8551329; doi:10.1038/s41598-021-00533-x)
Supplement: Supplementary file 6 — Supplementary Table 2. [file 41598_2021_533_MOESM6_ESM.pdf]

Nutritional restriction during the peri-conceptional period alters the myometrial transcriptome during the peri-implantation period

Ewa Monika Drzewiecka, Wiktoria Kozlowska, Agata Zmijewska, Anita Franczak*

Affiliation: Department of Animal Anatomy and Physiology, University of Warmia and Mazury in Olsztyn, Oczapowskiego 1A, 10-719 Olsztyn, Poland

*Corresponding Author: Anita Franczak, Department of Anatomy and Animal Physiology, Faculty of Biology and Biotechnology, University of Warmia and Mazury in Olsztyn, Oczapowskiego 1A, 10-719 Olsztyn, Poland; e-mail: anitaf@uwm.edu.pl

Supplementary table 2. Gene ontologies of genes with altered expression in the myometrium of pigs fed a restrictive diet compared to the myometrium of pigs during the peri-implantation period that were fed a restrictive diet during the peri-conceptional period comparing to the myometrium of pigs during the peri-implantation period that were fed a normal diet during the peri-conceptional period. GO – gene ontology, FDR – false discovery rate.

**UP-REGULATION**									
**GOTERM_BP_DIRECT**									
**GO**	**Term**	**Genes**	**Count**	**P-Value**	**Fold Enrichment**	**Bonferroni**	**Benjamini**	**FDR**	**Fisher Exact**
GO:0055114	oxidation-reduction process	*CYB5A, KDM5C, MSMO1, HMGCR, IFI30, SOD2, FAM213A, PRDX6, HSD17B8, DHRS4, CRYZ, GLUD1, LDHB, ALDH2, FTH1, CAT, ME1, CYB561*	18	5.83E-05	3.1	0.10	0.11	0.11	0.05
**GOTERM_CC_DIRECT**									
**GO**	**Term**	**Genes**	**Count**	**P-Value**	**Fold Enrichment**	**Bonferroni**	**Benjamini**	**FDR**	**Fisher Exact**

GO:0070062	extracellular exosome	*APP, SYAP1, ICAM3, GHITM, LIPA, CLU, COMP, LGALS3, GJA1, AKR7A2, LGALS1, PPP2R1B, PLAU, STMN1, CAPN2, ARL6IP5, CPNE3, SNRPD3, GLUL, ANXA11, SDHB, DCTD, MINPP1, RBP4, PPA1, CDC37, SCNN1A, CSNK2B, PSME1, SRSF2, VDAC2, FKBP4, ATP6V1A, GNAZ, RAB5C, TMED10, CFI, LTBP2, SLC1A5, FBLN5, PRDX3, ATP5B, KIF3B, MYO6, PSAP, ST3GAL4, IGFBP7, TSPAN3, IGFBP6, PROM1, HSPA5, IDH1, FUCA1, MSN, RAB27B, EIF2S1, PRDX6, COPS4, ARPC2, FXYD3, PTPRA, REEP2, GNAS, PI4KA, FAT1, TAB3, FBN1, MESP2, GPI, ARF4, CD40, SLC25A3, ARF1, COL14A1, ARPC1B, MAGEF1, ARPC1A, PRKAG1, ETFA, CHD2, RPS4X, TUBA1B, FLOT1, ACP5, CTSH, CTSC, YWHAH, CTSB, ARHGEF12, ANXA2, TUBB, ATP5F1, CKAP4, RHOA, RHOB, HADHB, ACTA1, UGDH, PKM, NPC1, NPC2, COL6A2, CAT, UFC1, ANGPTL2, TAX1BP3, SLC25A5, ARF5, DNASE1L1, OTUB1, STX12, PDXK, GRN, PCNA, SRC, GBA, ATP5A1, SRSF1, CRYZ, PPP2CA, PGRMC1, SAMM50, GNA11, KIAA1324, CD59, BID, CYB5A, LUM, CMTM6, LAMB1, FAM213A, RAB11B, TMEM59, PSAT1, BAX, CTNNB1, VIM, RBMX, RAN*	139	4.06E-19	2.14	0.00	0.00	0.00	0.00
GO:0005764	lysosome	*CD164, SRC, HEXA, GLMP, CTSV, IFI30, PRDX6, CLN5, TMEM59, GJA1, NPC1, NPC2, KIAA1324, PSAP, CAPN2, ACP5, CTSH, CTSD, CTSC, CTSB*	20	2.45E-07	4.18	0.00	0.00	0.00	0.00

GO:0005829	cytosol	*ARF4, ARF1, AMD1, CLU, GJA1, AIFM2, STMN1, CAPN2, ME1, CTSH, CPNE3, SNRPD3, KPNA2, PGM1, KPNA1, ANXA2, NFKBIL1, RHOA, NISCH, ARHGAP10, UGDH, CDC37, PEX6, GEMIN5, BIRC5, SYNC, ATP6V1A, PDXK, SRC, HIF1A, RELA, CRYZ, PPP2CA, NFS1, SNX1, BAG3, LCMT2, BID, SNX5, MCL1, TUB, IGBP1, IDH1, MX1, GLMP, GCK, COPS4, CDK5, EIF6, CDK4, CARM1, GNAS, BAX, CTNNB1, VIM*	55	9.42E-06	1.87	0.00	0.00	0.00	0.00
GO:0043209	myelin sheath	*ATP6V1A, GPI, SLC25A3, HSPA5, ATP5A1, MSN, SOD2, ATP5F1, PRDX3, ATP5B, TUBA1B, PKM, VDAC2, DLAT, SLC25A5, GLUL, LOC100156879*	17	1.69E-05	3.61	0.01	0.00	0.00	0.00
GO:0005737	cytoplasm	*APP, EIF4A3, PREP, BZW1, ETS2, IPO5, EFTUD2, RPS6KA3, GJA1, PTTG1, FTH1, DHX58, STMN1, STK16, CAPN2, PAPOLA, PLS3, KPNA2, TRIM21, RNF111, KPNA1, GTF2I, IFRD1, TPM1, EEF1A1, ACLY, DAAM1, PRKAR1A, PPA1, CDC37, CSNK2B, PSME1, PPIH, ATF4, FTL, LOC100037956, PXK, ANAPC16, UBE2J1, LDHB, DHX32, ATXN1, MYO6, WNT2, SMAD2, FDPS, PMM1, NEK4, MSN, HTR1B, FAM136A, EIF2S1, PRDX6, AZIN1, PBX1, COPS5, CDK4, NABP2, PI4KA, PDCL, TAB3, PI4KB, INTS9, PKIG, ZCCHC2, CUTC, GPI, BTG3, DYRK2, SMG7, SMG5, GLI2, THTPA, IKBKB, GYS1, CAPZB, DGCR8, CLIC1, YWHAH, LYPLA1, ARHGEF12, RHOA, ENAH, ACTA1, TUBB2B, PEX6, UFC1, ADSSL1, BIRC5, TAX1BP3, UMPS, ARF5, RBKS, OAT, PCNA, GMPR2, SRSF1, PRUNE1, RELA, ARNTL, PPP2CA, DNPEP, PSMB5, KCTD20, GNA11, RAE1, DCPS, ABCF1, ATE1, NTRK3, MX1, XPO7, CFLAR, LOC100049695, LOC100510930,*	125	1.94E-05	1.42	0.01	0.00	0.00	0.00

		KLF4, FAM213A, GSTZ1, FABP3, TNIP1, PSAT1, OTULIN, CCDC6, CTNNB1, RAN							
GO:0031012	extracellular matrix	*ANXA2, COL14A1, MMP2, TUBB, LTBP2, CLU, COMP, LGALS3, HADHA, VCAN, LGALS1, PKM, IGFBP7, VIM*	14	6.96E-05	3.80	0.03	0.00	0.00	0.00
GO:0005777	peroxisome	*GNPAT, ACOX1, PEX6, IDH1, PMVK, CAT, TYSND1, VIM, NUDT12, DHRS4*	10	7.01E-05	5.43	0.03	0.00	0.00	0.00
GO:0005884	actin filament	*ACTA1, CAPZB, TPM3, SRC, TPM1, PLS3, FYN, TEK*	8	1.63E-04	6.52	0.07	0.01	0.01	0.00
GO:0045121	membrane raft	*CLIP3, STX12, APP, GJA1, NPC1, ANXA2, PPP2R1B, CAPN2, FLOT1, PECAM1, FYN, TEK*	12	4.30E-04	3.62	0.17	0.02	0.02	0.00
GO:0005759	mitochondrial matrix	*CS, GLUD1, NFS1, ALDH2, MDH2, PCCB, DLAT, HADH, SOD2, TFB1M, HSD17B8, MCL1*	12	4.79E-04	3.58	0.18	0.02	0.02	0.00
GO:0005925	focal adhesion	*ARF1, HSPA5, ARPC1B, MSN, RPL8, RHOA, RHOB, ENAH, RPS4X, GJA1, ARPC2, PLAU, CAT, FAT1, PI4KA, CAPN2, FLOT1, CTNNB1, CPNE3, CD59, VIM, ITGA5*	22	5.44E-04	2.32	0.21	0.02	0.02	0.00
GO:0005789	endoplasmic reticulum membrane	*CYB5A, TMED10, SDF2L1, SAR1B, RPN1, MX1, MSMO1, HMGCR, DDOST, RTN4, PGRMC1, FXYD3, TMX1, MFSD3, REEP2, TRIM13, ARL6IP5, BAX, FKBP4, CDS2*	20	1.24E-03	2.28	0.41	0.04	0.04	0.00

GO:0005741	mitochondrial outer membrane	*HADHB, BNIP3L, VDAC1P5, MTX1, AIFM2, VDAC2, BAX, CPT1B, MCL1*	9	2.08E-03	3.86	0.59	0.07	0.07	0.00
GO:0005743	mitochondrial inner membrane	*SLC25A3, MTFP1, ABCB7, SRC, ATP5A1, SOD2, SDHB, HADHB, LGALS3, HADHA, GLUD1, SAMM50, VDAC2, HADH, SLC25A5*	15	3.12E-03	2.46	0.74	0.09	0.09	0.00
GO:0042470	melanosome	*HSPA5, ANXA2, RPN1, FLOT1, RAB27B, CTSD, CTSB*	7	4.66E-03	4.39	0.86	0.13	0.13	0.00
GO:0042645	mitochondrial nucleoid	*HADHB, HADHA, ATP5B, VDAC2, SLC25A5, TFB1M*	6	5.02E-03	5.24	0.88	0.13	0.13	0.00
GO:0016020	membrane	*RPL10, CFI, EIF4A3, HEXA, PRKAG1, PREP, ICAM3, RPL8, MED15, BZW1, GLI2, EFTUD2, RPS4X, GYS1, PSTPIP1, PGRMC1, KIF3B, CAPZB, GNA11, STMN1, KDSR, KPNA2, CTSC, JAK1, GTF2I, TYSND1, ANXA11, EIF2S1, PRDX6, RHOA, VCAN, NCEH1, OAS2, REEP3, GNAQ, PSMC1, GNAS, DRG1, CD9, GEMIN5, PLIN2, TMEM19, RBMX, RAN*	44	5.62E-03	1.52	0.91	0.14	0.13	0.00
GO:0005881	cytoplasmic microtubule	*CLIP2, TUBA1B, REEP2, BIRC5, SRPRB, MAPRE1*	6	5.87E-03	5.06	0.92	0.14	0.13	0.00
GO:0005739	mitochondrion	*ATP6V1A, GFM1, OAT, MTFP1, DIABLO, FITM2, ABCB8, GHITM, CLU, MRPL35, PRDX3, GJA1, AKR7A2, SPTLC2, PSAP, ME1, CDK5RAP1, SLC25A23, GLUL, DCPS, CTSB, CYB5A, MMP2, IDH1, UROS, IDH2, GATC,*	43	6.80E-03	1.51	0.94	0.15	0.15	0.00

		APEX2, FAM213A, CPT1B, MRPL24, GCK, RHOA, RAB11B, DHRS4, GSTZ1, HADHB, PKM, COPS5, ACOX1, APEX1, CAT, CRY1							
GO:0016607	nuclear speck	*EFTUD2, WTAP, APEX1, EIF4A3, SRSF1, SRSF2, PPIH, HIF1A, GLI2*	9	1.04E-02	2.97	0.99	0.22	0.21	0.00
GO:0008180	COP9 signalosome	*COPS4, COPS5, HSPA5, ATP5A1, FLOT1*	5	1.31E-02	5.31	1.00	0.27	0.26	0.00
GO:0005615	extracellular space	*UABP-2, APP, GPI, COL14A1, SAA3, CFI, GBA, LTBP2, VLDLR, CTSV, CLU, FBLN5, COMP, LGALS3, LGALS1, AIFM2, PLAU, C1QTNF6, NPY, PSAP, ENPP2, CTSH, IGFBP7, CD59, IGFBP6, PROM1, WNT2, CTSD, CTSC, CTSB, NCOA5, ANXA2, LUM, MMP2, LAMB1, PRDX6, RBP4, VCAN, BMP1, COL6A2, CD109, FMOD, RBMX, FBN1*	44	1.93E-02	1.41	1.00	0.37	0.36	0.01
GO:0005719	nuclear euchromatin	*CREB1, CTNNB1, KLF4, RBMX*	4	2.13E-02	6.52	1.00	0.39	0.38	0.00
GO:0030176	integral component of endoplasmic reticulum membrane	*SLC35B2, SLC35B4, SPCS1, SGMS1, HSPA5, SARAF, FITM2*	7	2.21E-02	3.17	1.00	0.39	0.38	0.01
GO:0030175	filopodium	*ACTA1, CDK5, FAT1, MSN, CD302*	5	3.25E-02	4.07	1.00	0.55	0.53	0.01

GO:0005654	nucleoplasm	*GPI, SMARCB1, DYRK2, PPP1R10, EIF4A3, SYAP1, ZDHHC7, CHD2, ETS2, IPO5, PAPOLA, DGCR8, SNRPD3, HADH, KPNA2, KPNA1, GTF2I, SH3D19, NFKBIL1, ANXA11, ATP5F1, SDHB, UGDH, CREB1, ACOX1, NDUFS2, FKBP4, PDXK, PCNA, SRSF1, ISG20L2, NFS1, FZR1, DNPEP, MYO6, DCPS, ABCF1, FDPS, STN1, KLF4, APTX, HNRNPAB, COPS4, COPS5, TNIP1, CARM1, PSMC1, NABP2, TAB3, RAN*	50	3.35E-02	1.33	1.00	0.55	0.53	0.02
GO:0005680	anaphase-promoting complex	*FZR1, ANAPC16, ANAPC7, ANAPC5*	4	3.50E-02	5.43	1.00	0.55	0.53	0.01
GO:0030027	lamellipodium	*ACTA1, ABLIM1, CAPZB, CDK5, FAT1, FLOT1, CTNNB1, RHOA*	8	3.74E-02	2.54	1.00	0.57	0.55	0.01
GO:0031966	mitochondrial membrane	*GLUD1, NDUFAF4, SLC25A32, CLU, ABCG2*	5	4.00E-02	3.82	1.00	0.57	0.55	0.01
GO:0005783	endoplasmic reticulum	*LRPAP1, CERS4, SRPX, BNIP3L, TPBG, SSR2, CLU, SGPP1, CKAP4, EEF1B2, HADHB, GJA1, NSDHL, NPC1, NPC2, P4HA2, TMX1, LRRC8C, NCK2, KDSR, ITGA5, PROM1, DNASE1L1*	23	4.09E-02	1.55	1.00	0.57	0.55	0.02
GO:0030018	Z disc	*PPP3CB, CAPZB, BAG3, CTNNB1, LDB3, SYNC*	6	4.18E-02	3.12	1.00	0.57	0.55	0.01
GO:0005643	nuclear pore	*PIK3R4, XPO7, KPNA2, RAE1, KPNA1*	5	4.41E-02	3.70	1.00	0.58	0.57	0.01
GO:0008290	F-actin capping protein complex	*LOC100037956, CAPZB*	3	4.95E-02	8.15	1.00	0.58	0.57	0.00
GO:0008250	oligosaccharyltransferase complex	*RPN2, RPN1, DDOST*	3	4.95E-02	8.15	1.00	0.58	0.57	0.00
GO:0031588	nucleotide-activated protein kinase complex	*PRKAR1A, PRKAG1, PRKAB1*	3	4.95E-02	8.15	1.00	0.58	0.57	0.00

GO:0005885	Arp2/3 protein complex	*ARPC2, ARPC1B, ARPC1A*	3	4.95E-02	8.15	1.00	0.58	0.57	0.00
GO:0005778	peroxisomal membrane	*GNPAT, ACOX1, CAT, HMGCR*	4	5.86E-02	4.44	1.00	0.67	0.65	0.01
GO:0005697	telomerase holoenzyme complex	*SNRPD3, SMG7, SMG5*	3	6.03E-02	7.33	1.00	0.67	0.65	0.01
GO:0005811	lipid particle	*NSDHL, RAB5C, ANXA2, AIFM2, CKAP4*	5	6.80E-02	3.22	1.00	0.74	0.72	0.02
GO:0005874	microtubule	*IGBP1, TUBB2B, KIF3B, TUBB, STMN1, TCP11L1, MAPRE1, LOC100510930*	8	7.13E-02	2.20	1.00	0.76	0.73	0.03
GO:0032587	ruffle membrane	*ARF4, SRC, ITGA5, RHOA*	4	8.01E-02	3.91	1.00	0.83	0.80	0.02
GO:0030017	sarcomere	*ARF1, CAPZB, MMP2*	3	8.38E-02	6.11	1.00	0.83	0.80	0.01
GO:0042383	sarcolemma	*ANXA2, KCNB1, COL6A2, FLOT1, SYNC*	5	8.49E-02	2.98	1.00	0.83	0.80	0.03
GO:0005770	late endosome	*TMEM59, SRC, KIAA1324, PIK3R4, M6PR, RHOB*	6	8.73E-02	2.53	1.00	0.83	0.80	0.03
GO:0001725	stress fiber	*ACTA1, ABLIM1, DAAM1, TEK*	4	8.79E-02	3.76	1.00	0.83	0.80	0.02
GO:0031965	nuclear membrane	*FZR1, WTAP, CDK4, GNAQ, MYO6, MX1, CLIC1, IPO5*	8	9.33E-02	2.06	1.00	0.84	0.82	0.04
GO:0044297	cell body	*GNAZ, ACTA1, TUBB, GLUL*	4	9.60E-02	3.62	1.00	0.84	0.82	0.02

GO:0016328	lateral plasma membrane	*VANGL1, GJA1, KCNB1, CTNNB1*	4	9.60E-02	3.62	1.00	0.84	0.82	0.02
GO:0005768	endosome	*APP, CD164, NPC1, ARPC2, FLOT1, FYN, SNX5, RHOA*	8	9.74E-02	2.04	1.00	0.84	0.82	0.04
**GOTERM_MF_DIRECT**									
**GO**	**Term**	**Genes**	**Count**	**P-Value**	**Fold Enrichment**	**Bonferroni**	**Benjamini**	**FDR**	**Fisher Exact**
GO:0005525	GTP binding	*GNAZ, ARF4, ARF1, GFM1, RAB5C, SAR1B, SRP54, EFTUD2, TUBA1B, GNA11, GBP1, TUBB, MX1, RAB27B, LOC100510930, RHOA, RAB11B, RHOB, EEF1A1, TUBB2B, RIT1, RAB37, GNAQ, GNAS, ADSSL1, DRG1, FKBP4, RAN, ARF5*	29	1.06E-05	2.53	0.01	0.01	0.01	0.00
GO:0003924	GTPase activity	*GNAZ, GFM1, RAB5C, TUBB, MX1, SRP54, RAB27B, LOC100510930, RHOA, RAB11B, EEF1A1, EFTUD2, TUBA1B, TUBB2B, GNAQ, GNA11, GNAS, GBP1, RAN*	19	3.53E-05	3.12	0.02	0.01	0.01	0.00
GO:0044822	poly(A) RNA binding	*GRN, ARF1, GFM1, COL14A1, PPP1R10, RPP30, ATP5A1, RPN1, SRP54, ISG20L2, RBM4B, SNU13, TFB1M, RPL8, CHD2, BZW1, IPO5, EFTUD2, RPS4X, LGALS3, DHX32, LGALS1, KIAA1324, LOC100152229, CPNE3, SNRPD3, KPNA2, NELFE, FDPS, NCOA5, ANXA2, ANXA11, EIF2S1, HNRNPAB, CKAP4, BICC1, HADHB, PKM, APEX1, PSMC1, SRSF2, GEMIN5, SLC25A5, FKBP4, MAPRE1, EIF1B, RAN*	47	2.79E-04	1.73	0.15	0.05	0.05	0.00
GO:0003857	3-hydroxyacyl-CoA dehydrogenase activity	*HADHB, HADHA, HADH, HSD17B8*	4	1.26E-03	16.19	0.51	0.18	0.18	0.00

GO:0051015	actin filament binding	*CAPZB, TPM3, ARPC1B, TPM1, ARPC1A, MYO6, PLS3*	7	2.74E-03	4.86	0.79	0.31	0.31	0.00
GO:0019003	GDP binding	*RAB5C, SRP54, RAB27B, RAN, RHOA, RAB11B, RHOB*	7	4.20E-03	4.47	0.91	0.39	0.39	0.00
GO:0003725	double-stranded RNA binding	*TUBA1B, OAS2, DHX58, DGCR8, MSN, VIM, APTX*	7	5.45E-03	4.25	0.95	0.44	0.44	0.00
GO:0031683	G-protein beta/gamma-subunit complex binding	*GNAZ, GNAQ, GNA11, GNAS*	4	1.45E-02	7.47	1.00	0.91	0.91	0.00
GO:0005200	structural constituent of cytoskeleton	*TUBA1B, TUBB2B, ARPC2, ARPC1B, TUBB, VIM, LOC100510930*	7	1.46E-02	3.47	1.00	0.91	0.91	0.00
**DOWN-REGULATION**									
**GOTERM_CC_DIRECT**									
**GO**	**Term**	**Genes**	**Count**	**P-Value**	**Fold Enrichment**	**Bonferroni**	**Benjamini**	**FDR**	**Fisher Exact**
GO: 0005654	nucleoplasm	*RSC1A1, TOP2B, SMC3, IPO7, ORC4, PNN, TATDN1, ZMYM4, PLAGL1, HOXA3, UBXN7, KPNA5, ARID2, SRSF11, RBM14, HNRNPA3, STN1, POU2F1, MICAL3, PRPF40A, THNSL1, AFTPH, ARL4A, RYBP, DDX39B, RRM2B, PPIG, BDP1, KIAA1958*	29	1.85E-04	2.13	0.04	0.04	0.04	0.00
GO:0005829	cytosol	*YAP1, TOP2B, MOCS2, GCC2, IPO7, ZFP36L1, EEA1, NLN, CCDC88A, PIKFYVE, AFTPH, SNX16, ECHDC1, MUC13, INPP5K, KPNA5, PAXBP1, CDO1, OGT, SRSF10*	20	9.59E-03	1.88	0.86	0.71	0.71	0.00

GO	Term	Genes	Count	P-Value	Fold Enrichment	Bonferroni	Benjamini	FDR	Fisher Exact
GO:0017053	transcriptional repressor complex	*LOC100621006, REST, C1D, ARID4A*	4	1.04E-02	8.72	0.88	0.71	0.71	0.00
GO:0031410	cytoplasmic vesicle	*EEA1, ITGB1, CCDC88A, PIKFYVE, LRP6*	5	1.62E-02	5.12	0.96	0.83	0.83	0.00
GO:0070469	respiratory chain	*ND6, COX2, ND2*	3	2.26E-02	12.66	0.99	0.93	0.93	0.00
**GOTERM_MF_DIRECT**									
**GO**	**Term**	**Genes**	**Count**	**P-Value**	**Fold Enrichment**	**Bonferroni**	**Benjamini**	**FDR**	**Fisher Exact**
GO:0000166	nucleotide binding	*ORC4, RBM25, HNRNPA3, RBM14, SSB, CPSF6, LARP7, SLIRP, TARDBP, HNRNPLL, SRSF10, SRSF11*	12	1.55E-03	3.13	0.30	0.36	0.36	0.00
GO:0000981	RNA polymerase II transcription factor activity, sequence-specific DNA binding	*LOC100621006, MEF2C, REST, NFIB, ZMYM4, PLAGL1, KIAA1958, FOXP2*	8	6.08E-03	3.67	0.76	0.71	0.71	0.00
GO:0046872	metal ion binding	*ITGB1, ZNF582, ZBTB44, NT5C2, MUT, ACACB, CMAH, ZFP36L1, KLF17, EEA1, LOC100621006, NLN, REST, RRM2B, ZKSCAN7, PLAGL1, RUFY2, PDE4B, ZNF512, ARID2, PELO, ZC3H15, ZNF398*	23	1.05E-02	1.76	0.91	0.81	0.81	0.01
GO:0003682	chromatin binding	*LOC100621006, BRD3, YAP1, TOP2B, MEF2C, REST, ATRX, PBX2, TFAM, SMC3*	10	1.55E-02	2.58	0.97	0.90	0.90	0.01
GO:0003779	actin binding	*CAPZA2, RDX, KLHL2, CAP2*	4	1.98E-02	6.86	0.99	0.92	0.92	0.00
