# Supplementary material for: Nutritional restriction during the peri-conceptional period alters the myometrial transcriptome during the peri-implantation period
Source: Sci Rep. 2021 Oct 27;11:21187. doi: 10.1038/s41598-021-00533-x (PMC8551329; doi:10.1038/s41598-021-00533-x)
Supplement: Supplementary file 7 — Supplementary Table 3. [file 41598_2021_533_MOESM7_ESM.pdf]

Nutritional restriction during the peri-conceptional period alters the myometrial transcriptome during the peri-implantation period

Ewa Monika Drzewiecka, Wiktoria Kozlowska, Agata Zmijewska, Anita Franczak*

Affiliation: Department of Animal Anatomy and Physiology, University of Warmia and Mazury in Olsztyn, Oczapowskiego 1A, 10-719 Olsztyn, Poland

*Corresponding Author: Anita Franczak, Department of Anatomy and Animal Physiology, Faculty of Biology and Biotechnology, University of Warmia and Mazury in Olsztyn, Oczapowskiego 1A, 10-719 Olsztyn, Poland; e-mail: anitaf@uwm.edu.pl

Supplementary table 3. Biological pathways, in the myometrium of pigs during the peri-implantation period that were fed a restrictive diet during the peri-conceptional period comparing to the myometrium of pigs during the peri-implantation period that were fed a normal diet during the peri-conceptional period, in which genes with altered expression (up-regulated and down-regulated ones) are involved - analysis performed using DAVID tool in accordance to the bioinformatical database KEGG (Kanehisa, M. & Goto, S. KEGG: Kyoto Encyclopedia of Genes and Genomes. *Nucleic Acids Research* 28, 27–30 (2000); Kanehisa, M. Toward understanding the origin and evolution of cellular organisms. *Protein Sci.* 28, 1947–1951 (2019); Kanehisa, M., Furumichi, M., Sato, Y., Ishiguro-Watanabe, M. & Tanabe, M. KEGG: Integrating viruses and cellular organisms. *Nucleic Acids Res.* 49, D545–D551 (2021).) FDR – false discovery rate.

.

Term	Count	%	P-Value	Genes	List Total	Pop Hits	Pop Total	Fold Enrichment	Bonferroni	Benjamini	FDR
ssc01200: Carbon metabolism	20	2.14	1.41E-05	GPI, MDH2, IDH1, IDH2, MUT, SDHB, GCK, RPIA, CS, GLUD1, HADHA, PKM, PSAT1, CAT, PCCB, RGN, ME1, DLAT, HAO2, HIBCH	434	103	7001	3.13	0.004	0.002	0.002
ssc01130: Biosynthesis of antibiotics	29	3.11	1.49E-05	GPI, OAT, MSMO1, HMGCR, LDHB, NSDHL, ALDH2, DLAT, HADH, HAO2, PGM1, FDPS, MDH2, IDH1, IDH2, BCKDHB, SDHB, GCK, RPIA, CS, HADHB, HADHA, ACLY, SQLE, PKM, PSAT1, CAT, PCCB, RGN	434	191	7001	2.45	0.004	0.002	0.002

ssc01100: Metabolic pathways	102	10.93	1.17E-04	CHPF, MSMO1, ACSM5, NSDHL, FPGT, HADH, SEPHS2, GLUL, ALG5, ALG2, ALG10, SDHB, UPB1, DCTD, MINPP1, ACLY, ND2, ND4, DGKH, ATP6V1A, ND6, RPN2, RPN1, ACACB, HSD11B1, LDHB, ATP5B, RDH10, PMVK, ST3GAL4, COX2, INPP5K, KDSR, HAO2, FDPS, PMM1, IDH1, UROS, IDH2, PRDX6, CS, GLUD1, SQLE, P4HA2, PI4KA, PI4KB, CDS2, GPI, MOCS2, GBE1, HEXA, AMD1, THTPA, SMPD2, SPTLC1, SPTLC2, ALDH2, ME1, DLAT, HIBCH, PGM1, CERS4, BCKDHB, ATP5F1, MUT, DDOST, HADHB, HADHA, UGDH, PKM, RRM2B, B3GNT6, ACOX1, PCCB, RGN, ADSSL1, NDUFS2, PIGL, UMPS, PAFAH1B2, PRIM2, PDXK, OAT, SGMS1, GBA, ATP5A1, HMGCR, AGPAT1, NT5C2, HSD17B8, NFS1, MDH2, GATC, GCK, DHRS4, RPIA, GSTZ1, PSAT1, POLR3H, CDO1, LOC100156879, PNPLA2	434	1161	7001	1.42	0.032	0.011	0.010
ssc04922: Glucagon signaling pathway	17	1.82	2.06E-04	PRKAG1, PHKA1, CPT1C, PRKAB1, ACACB, CPT1B, GCK, GYS1, LDHB, PPP3CB, CREB1, PKM, GNAQ, AKT3, GNAS, EP300, ATF4	434	95	7001	2.89	0.055	0.014	0.013
ssc04145: Phagosome	21	2.25	4.67E-04	ATP6V1A, ITGB1, STX12, DYNC1H1, RAB5C, SLA-5, ITGB3, TUBB, M6PR, LOC100624785, TAP1, CTSV, LOC100510930, EEA1, SEC61A2, COMP, PIKFYVE, DYNC1LI2, TUBA1B, TUBB2B, ITGA5	434	143	7001	2.37	0.120	0.022	0.021
ssc04071: Sphingolipid signaling pathway	18	1.93	4.73E-04	CERS4, MAP2K1, SGMS1, PDPK1, RELA, SGPP1, RHOA, PPP2CA, SMPD2, SPTLC1, SPTLC2, PPP2R1B, GNAQ, AKT3, BAX, FYN, BID, CTSD	434	112	7001	2.59	0.122	0.022	0.021
ssc04141: Protein	21	2.25	0.0018	XBP1, HSPA5, RPN2, HSPA1L, SAR1B, EDEM1, SSR2, AMFR, RPN1, RRBP1,	434	159	7001	2.13	0.390	0.070	0.067

processing in endoplasmic reticulum				EIF2S1, DDOST, CKAP4, UBE2J1, SEC61A2, CAPN2, BAX, SEC62, ATF4, SEC31A, TXNDC5							
ssc04142: Lysosome	17	1.82	0.002142	CD164, GBA, HEXA, M6PR, CTSV, LIPA, CLN5, NPC1, NPC2, PSAP, ACP5, CTSH, AP4S1, CTSD, CTSC, LGMN, CTSB	434	117	7001	2.34	0.444	0.073	0.070
ssc03060: Protein export	7	0.75	0.003492	SEC61A2, SRP19, SPCS1, HSPA5, SRP54, SRPRB, SEC62	434	25	7001	4.52	0.617	0.087	0.083
ssc00640: Propanoate metabolism	7	0.75	0.003492	HADHA, LDHB, PCCB, ECHDC1, MUT, ACACB, HIBCH	434	25	7001	4.52	0.617	0.087	0.083
ssc00630: Glyoxylate and dicarboxylate metabolism	7	0.75	0.003492	CS, MDH2, CAT, PCCB, GLUL, HAO2, MUT	434	25	7001	4.52	0.617	0.087	0.083
ssc05216: Thyroid cancer	7	0.75	0.004307	RXRB, TCF7L1, MAP2K1, TPM3, NCOA4, CCDC6, CTNNB1	434	26	7001	4.34	0.694	0.098	0.094
ssc05166: HTLV-I infection	27	2.89	0.005427	CD40, PCNA, SLA-5, RELA, ETS2, IKBKB, VDAC1P5, PPP3CB, PTTG1, AKT3, EP300, WNT2, JAK1, SMAD2, FDPS, XBP1, ANAPC7, CREB1, CDK4, VDAC2, CTNNB1, BAX, ANAPC5, SLC25A5, TLN1, RAN, ATF4	434	248	7001	1.76	0.775	0.112	0.107
ssc03040: Spliceosome	17	1.82	0.005738	RBM25, HNRNPA3, DDX46, HSPA1L, EIF4A3, SRSF1, SNU13, PRPF40A, LSM5, EFTUD2, DDX39B, SRSF2, PPIH, SNRPD3, SNRPC, RBMX, SRSF10	434	129	7001	2.13	0.793	0.112	0.107
ssc00620: Pyruvate metabolism	8	0.86	0.006724	LDHB, PKM, ALDH2, MDH2, GLO1, ME1, DLAT, ACACB	434	37	7001	3.49	0.843	0.123	0.117
ssc04931: Insulin resistance	15	1.61	0.008842	PDPK1, INSR, PRKAG1, PTPN11, PRKAB1, ACACB, CPT1B, RELA, IKBKB, RPS6KA3, GYS1, CREB1, PPP1R3C, AKT3, OGT	434	112	7001	2.16	0.912	0.145	0.138
ssc00020: Citrate cycle (TCA cycle)	7	0.75	0.009009	CS, ACLY, MDH2, IDH1, IDH2, DLAT, SDHB	434	30	7001	3.76	0.916	0.145	0.138

ssc04920: Adipocytokine signaling pathway	11	1.18	0.011403	IKBKB, RXRB, NPY, AKT3, PRKAG1, PTPN11, CPT1C, PRKAB1, ACACB, CPT1B, RELA	434	71	7001	2.50	0.957	0.174	0.165
ssc00600: Sphingolipid metabolism	8	0.86	0.017265	SMPD2, CERS4, SPTLC1, SGMS1, SPTLC2, GBA, KDSR, SGPP1	434	44	7001	2.93	0.992	0.242	0.230
ssc04022: cGMP-PKG signaling pathway	18	1.93	0.017658	MEF2C, MAP2K1, KCNJ8, INSR, ATP2B4, ATP2A2, ADRA2B, RHOA, VDAC1P5, PPP3CB, CREB1, GNAQ, GNA11, AKT3, VDAC2, SLC25A5, ATF4, GTF2I	434	158	7001	1.84	0.992	0.242	0.230
ssc00280: Valine, leucine and isoleucine degradation	8	0.86	0.02687	HADHB, HADHA, ALDH2, BCKDHB, PCCB, HADH, MUT, HIBCH	434	48	7001	2.69	0.999	0.331	0.315
ssc05164: Influenza A	18	1.93	0.028658	IFNAR2, FDPS, MAP2K1, HSPA1L, IFNGR1, MX1, EIF2S1, RELA, IKBKB, DDX39B, OAS2, AKT3, CYCS, EP300, KPNA2, RAE1, JAK1, KPNA1	434	167	7001	1.74	1.000	0.331	0.315
ssc03015: mRNA surveillance pathway	11	1.18	0.028992	PPP2CA, PNN, CPSF6, PPP2R1B, DDX39B, CPSF3, EIF4A3, PAPOLA, SMG7, PELO, SMG5	434	82	7001	2.16	1.000	0.331	0.315
ssc04146: Peroxisome	11	1.18	0.028992	GNPAT, ACOX1, PEX6, IDH1, IDH2, PMVK, CAT, SOD2, HAO2, NUDT12, DHRS4	434	82	7001	2.16	1.000	0.331	0.315
ssc00520: Amino sugar and nucleotide sugar metabolism	8	0.86	0.032819	UGDH, GPI, PMM1, FPGT, HEXA, CMAH, GCK, PGM1	434	50	7001	2.58	1.000	0.360	0.343
ssc00071: Fatty acid degradation	7	0.75	0.034681	HADHB, HADHA, ALDH2, ACOX1, HADH, CPT1C, CPT1B	434	40	7001	2.82	1.000	0.365	0.348
ssc04540: Gap junction	11	1.18	0.044194	TUBA1B, GJA1, TUBB2B, MAP2K1, SRC, GNAQ, TUBB, GNA11, GNAS, LOC100624785, LOC100510930	434	88	7001	2.02	1.000	0.434	0.414

ssc05205: Proteoglycans in cancer	19	2.04	0.045904	ITGB1, MAP2K1, ARHGEF12, PDPK1, LUM, SRC, ITGB3, MMP2, RDX, MSN, PTPN11, CTSV, HIF1A, RHOA, PLAU, AKT3, CTNNB1, ITGA5, WNT2	434	190	7001	1.61	1.000	0.434	0.414
ssc05161: Hepatitis B	16	1.71	0.04598	MAP2K1, PCNA, SRC, RELA, IKBKB, VDAC1P5, CREB1, CASP10, CDK4, AKT3, CYCS, BIRC5, EP300, BAX, JAK1, ATF4	434	151	7001	1.71	1.000	0.434	0.414
ssc04612: Antigen processing and presentation	9	0.96	0.049488	CREB1, HSPA1L, SLA-5, PSME1, TAP1, CTSV, IFI30, LGMN, CTSB	434	66	7001	2.20	1.000	0.440	0.419
ssc04110: Cell cycle	13	1.39	0.051219	SMAD2, PCNA, ANAPC7, GADD45A, SMC3, ORC4, FZR1, PTTG1, CDK4, RAD21, EP300, ANAPC5, YWHAH	434	115	7001	1.82	1.000	0.440	0.419
ssc00010: Glycolysis / Gluconeogenesis	8	0.86	0.05135	GPI, LDHB, MINPP1, PKM, ALDH2, DLAT, GCK, PGM1	434	55	7001	2.35	1.000	0.440	0.419
ssc04152: AMPK signaling pathway	13	1.39	0.057064	PDPK1, INSR, PRKAG1, HMGCR, CPT1C, PRKAB1, CPT1B, RAB11B, PPP2CA, GYS1, CREB1, PPP2R1B, AKT3	434	117	7001	1.79	1.000	0.462	0.440
ssc05210: Colorectal cancer	9	0.96	0.057312	SMAD2, TCF7L1, MAP2K1, AKT3, BIRC5, CYCS, CTNNB1, BAX, RHOA	434	68	7001	2.14	1.000	0.462	0.440
ssc04910: Insulin signaling pathway	14	1.50	0.060794	MAP2K1, PDPK1, INSR, PRKAG1, PHKA1, PRKAB1, ACACB, GCK, IKBKB, GYS1, PPP1R3C, PRKAR1A, AKT3, FLOT1	434	131	7001	1.72	1.000	0.468	0.446
ssc04210: Apoptosis	9	0.96	0.061502	IKBKB, CASP10, AKT3, CAPN2, CYCS, BAX, CFLAR, BID, RELA	434	69	7001	2.10	1.000	0.468	0.446
ssc05016: Huntington's disease	19	2.04	0.066254	ATP5A1, SOD2, ATP5F1, SDHB, LOC100621006, ATP5B, REST, VDAC1P5, CREB1, GNAQ, CYCS, NDUFS2, COX2, TFAM, EP300, VDAC2, BAX, SLC25A5, LOC100156879	434	199	7001	1.54	1.000	0.483	0.460

ssc04932: Non-alcoholic fatty liver disease (NAFLD)	16	1.71	0.069337	XBP1, INSR, PRKAG1, PRKAB1, EIF2S1, SDHB, RELA, IKBKB, AKT3, CYCS, NDUFS2, COX2, BAX, BID, LOC100156879, ATF4	434	160	7001	1.61	1.000	0.483	0.460
ssc04520: Adherens junction	9	0.96	0.070443	SMAD2, TCF7L1, SRC, INSR, CSNK2B, EP300, CTNNB1, FYN, RHOA	434	71	7001	2.04	1.000	0.483	0.460
ssc05152: Tuberculosis	17	1.82	0.071344	ARHGEF12, RAB5C, IFNGR1, SRC, RELA, RHOA, EEA1, PPP3CB, CREB1, CASP10, AKT3, CYCS, EP300, BAX, BID, CTSD, JAK1	434	174	7001	1.58	1.000	0.483	0.460
ssc04710: Circadian rhythm	5	0.54	0.073306	CREB1, CRY1, PRKAG1, PRKAB1, ARNTL	434	26	7001	3.10	1.000	0.483	0.460
ssc05200: Pathways in cancer	32	3.43	0.074101	ITGB1, MAX, HIF1A, RELA, GLI2, IKBKB, RXRB, GNA11, AKT3, BDKRB2, EP300, BID, WNT2, JAK1, SMAD2, TCF7L1, MAP2K1, ARHGEF12, TPM3, MMP2, NCOA4, LAMB1, RHOA, TRAF4, CDK4, GNAQ, CCDC6, GNAS, BIRC5, CTNNB1, BAX, CYCS	434	384	7001	1.34	1.000	0.483	0.460
ssc05215: Prostate cancer	10	1.07	0.078319	IKBKB, TCF7L1, MAP2K1, CREB1, PDPK1, AKT3, EP300, CTNNB1, RELA, ATF4	434	85	7001	1.90	1.000	0.485	0.462
ssc05222: Small cell lung cancer	10	1.07	0.078319	IKBKB, RXRB, ITGB1, TRAF4, CDK4, MAX, AKT3, CYCS, LAMB1, RELA	434	85	7001	1.90	1.000	0.485	0.462
ssc05012: Parkinson's disease	15	1.61	0.079723	ND6, ATP5A1, ATP5F1, SDHB, UBE2J1, ATP5B, VDAC1P5, CYCS, NDUFS2, COX2, VDAC2, SLC25A5, ND2, LOC100156879, ND4	434	150	7001	1.61	1.000	0.485	0.462
ssc05412: Arrhythmogenic right ventricular cardiomyopathy (ARVC)	8	0.86	0.086603	ITGB1, RYR2, GJA1, TCF7L1, ITGB3, ITGA1, CTNNB1, ITGA5	434	62	7001	2.08	1.000	0.506	0.482

ssc03013: RNA transport	15	1.61	0.086713	EIF4A1, RPP30, EIF4A3, RPP40, EIF2S1, EEF1A1, PNN, DDX39B, GEMIN5, EIF3E, EIF2B1, EIF1B, RAE1, EIF3A, RAN	434	152	7001	1.59	1.000	0.506	0.482
ssc04144: Endocytosis	21	2.25	0.089829	SMAD2, LOC100037956, ARF1, RAB5C, HSPA1L, SLA-5, SRC, ARPC1B, ARPC1A, WWP1, RHOA, RAB11B, EEA1, SNX1, ARPC2, CAPZB, PSD3, CAPZA2, SNX5, ARF5	434	235	7001	1.44	1.000	0.513	0.488
ssc05100: Bacterial invasion of epithelial cells	9	0.96	0.096099	ITGB1, ARHGAP10, ARPC2, SRC, ARPC1B, ARPC1A, CTNNB1, ITGA5, RHOA	434	76	7001	1.91	1.000	0.532	0.507
ssc05168: Herpes simplex infection	17	1.82	0.09708	IFNAR2, SLA-5, IFNGR1, SRSF1, TAP1, PTPN11, EIF2S1, RELA, ARNTL, IKBKB, OAS2, CSNK2B, SRSF2, CYCS, EP300, JAK1, GTF2I	434	182	7001	1.51	1.000	0.532	0.507
