# Supplementary material for: Nutritional restriction during the peri-conceptional period alters the myometrial transcriptome during the peri-implantation period
Source: Sci Rep. 2021 Oct 27;11:21187. doi: 10.1038/s41598-021-00533-x (PMC8551329; doi:10.1038/s41598-021-00533-x)
Supplement: Supplementary file 8 — Supplementary Table 4. [file 41598_2021_533_MOESM8_ESM.pdf]

Nutritional restriction during the peri-conceptional period alters the myometrial transcriptome during the peri-implantation period

Ewa Monika Drzewiecka, Wiktoria Kozlowska, Agata Zmijewska, Anita Franczak*

Affiliation: Department of Animal Anatomy and Physiology, University of Warmia and Mazury in Olsztyn, Oczapowskiego 1A, 10-719 Olsztyn, Poland

*Corresponding Author: Anita Franczak, Department of Anatomy and Animal Physiology, Faculty of Biology and Biotechnology, University of Warmia and Mazury in Olsztyn, Oczapowskiego 1A, 10-719 Olsztyn, Poland; e-mail: anitaf@uwm.edu.pl

Supplementary table 4. Transcription factor enrichment analysis (TFEA) for genes with altered expression in the myometrium of pigs fed a restrictive diet compared to the myometrium of pigs during the peri-implantation period that were fed a restrictive diet during the peri-conceptional period comparing to the myometrium of pigs during the peri-implantation period that were fed a normal diet during the peri-conceptional period. TF – transcription factor, OR - odds ratio.

Accession	Cell	Treatment	TF	p.value	OR	log2.OR	adj.p.value	log10.adj.pVal	distance
wgEncodeEH000552	HUVEC	none	POLR2A	2.47E-82	4.67	2.22	2.85E-79	78.55	78.63
wgEncodeEH000606	ProgFib	none	POLR2A	2.76E-81	4.58	2.19	1.59E-78	77.80	77.88
wgEncodeEH002079	A549	none	POLR2A	8.21E-79	4.75	2.25	3.16E-76	75.50	75.59
wgEncodeEH000613	HeLa-S3	none	POLR2A	1.46E-78	4.68	2.23	4.22E-76	75.37	75.46
wgEncodeEH001136	Glioblasts	none	POLR2A	1.05E-76	4.63	2.21	2.41E-74	73.62	73.71
wgEncodeEH001493	A549	EtOH_0.02pct	POLR2A	1.42E-74	5.41	2.43	2.73E-72	71.56	71.70
wgEncodeEH001494	A549	DEX_100nM	POLR2A	1.24E-73	5.38	2.43	2.05E-71	70.69	70.82
wgEncodeEH001474	HeLa-S3	none	POLR2A	2.17E-72	4.62	2.21	3.13E-70	69.50	69.60
wgEncodeEH002301	SK-N-SH	none	TAF1	9.28E-71	3.80	1.93	1.19E-68	67.92	67.98
wgEncodeEH000714	GM15510	none	POLR2A	2.13E-70	4.13	2.05	2.46E-68	67.61	67.68
GSE105857	HepG2	none	NONO	2.45E-69	4.04	2.01	2.57E-67	66.59	66.66
wgEncodeEH000632	HEK293	none	POLR2A	2.81E-68	3.62	1.86	2.70E-66	65.57	65.62
wgEncodeEH001572	ECC-1	DMSO_0.02pct	POLR2A	1.06E-67	4.31	2.11	9.44E-66	65.02	65.11
GSM2758963	HUVEC	hypoxia	SIN3A	1.42E-67	4.09	2.03	1.17E-65	64.93	65.01
wgEncodeEH001627	HCT-116	none	POLR2A	3.28E-67	5.05	2.34	2.52E-65	64.60	64.72
wgEncodeEH001768	MCF10A-Er-Src	4OHTAM_1uM_36hr	POLR2A	4.90E-67	4.02	2.01	3.53E-65	64.45	64.52
wgEncodeEH001771	MCF10A-Er-Src	EtOH_0.01pct	POLR2A	6.56E-67	4.13	2.05	4.45E-65	64.35	64.43
wgEncodeEH002809	IMR90	none	POLR2A	9.48E-67	3.66	1.87	6.08E-65	64.22	64.27
wgEncodeEH002270	SK-N-SH	none	POLR2A	1.17E-66	4.35	2.12	7.09E-65	64.15	64.24

wgEncodeEH001674	U87	none	POLR2A	2.47E-66	4.01	2.00	1.43E-64	63.85	63.92
wgEncodeEH000597	HeLa-S3	none	POLR2A	3.84E-65	3.63	1.86	2.11E-63	62.68	62.73
wgEncodeEH002297	HUVEC	none	POLR2A	4.46E-65	3.75	1.91	2.34E-63	62.63	62.69
wgEncodeEH000733	GM18526	none	POLR2A	6.64E-65	3.71	1.89	3.33E-63	62.48	62.54
wgEncodeEH000739	GM18951	none	POLR2A	1.21E-64	4.00	2.00	5.80E-63	62.24	62.31
wgEncodeEH001582	K562	none	TAF1	1.42E-64	3.57	1.84	6.55E-63	62.18	62.24
GSE60584	HeLa	none	NELFE	1.90E-64	3.63	1.86	8.43E-63	62.07	62.13
wgEncodeEH000741	GM19099	none	POLR2A	6.72E-64	3.93	1.97	2.87E-62	61.54	61.61
wgEncodeEH001500	H1-hESC	none	TAF1	8.23E-64	4.25	2.09	3.39E-62	61.47	61.55
wgEncodeEH000731	GM18505	none	POLR2A	1.40E-63	3.82	1.93	5.56E-62	61.25	61.32
wgEncodeEH003435	MCF-7	serum_stimulated_media	POL2	1.84E-63	3.92	1.97	6.85E-62	61.16	61.23
wgEncodeEH000616	K562	none	POLR2A	1.84E-63	3.73	1.90	6.85E-62	61.16	61.23
wgEncodeEH000758	HepG2	forskolin	POLR2A	2.20E-63	3.86	1.95	7.95E-62	61.10	61.17
wgEncodeEH002298	HUVEC	none	POLR2A	1.69E-62	4.49	2.17	5.92E-61	60.23	60.33
wgEncodeEH001766	PBDE	none	POLR2A	6.33E-62	3.36	1.75	2.15E-60	59.67	59.72
wgEncodeEH002092	K562	none	PHF8	7.34E-62	3.84	1.94	2.42E-60	59.62	59.68
wgEncodeEH000662	K562	IFNg6h	POLR2A	7.79E-62	3.54	1.82	2.50E-60	59.60	59.66
wgEncodeEH000729	GM12892	none	POLR2A	1.29E-61	3.79	1.92	4.03E-60	59.39	59.46
wgEncodeEH001551	HepG2	none	TAF1	1.42E-61	3.59	1.85	4.32E-60	59.36	59.42
wgEncodeEH001499	H1-hESC	none	POLR2A	1.54E-61	3.64	1.86	4.44E-60	59.35	59.41

wgEncodeEH000618	NB4	none	POLR2A	1.51E-61	3.52	1.82	4.44E-60	59.35	59.41
wgEncodeEH003438	MCF-7	serum_starved_media	POLR2A	4.28E-61	3.72	1.90	1.21E-59	58.92	58.98
GSM1538429	LNCaP	none	CREB1	1.68E-60	5.19	2.38	4.39E-59	58.36	58.51
GSM1919983	PDAC	none	KLF6	1.52E-60	4.35	2.12	4.08E-59	58.39	58.48
wgEncodeEH000743	GM19193	none	POLR2A	1.47E-60	3.69	1.88	4.05E-59	58.39	58.46
GSE94782.2	Kelly	none	MYCN	3.36E-60	5.65	2.50	8.63E-59	58.06	58.25
GSM1526876	HMEC-TERT	none	E2F1	3.72E-60	3.85	1.94	9.29E-59	58.03	58.10
wgEncodeEH002278	HepG2	none	POLR2A	3.78E-60	3.50	1.81	9.29E-59	58.03	58.09
wgEncodeEH001792	HepG2	none	POLR2A	4.47E-60	3.48	1.80	1.08E-58	57.97	58.02
wgEncodeEH001761	Raji	none	POLR2A	6.91E-60	3.49	1.80	1.63E-58	57.79	57.84
wgEncodeEH001505	HeLa-S3	none	TAF1	1.71E-59	3.36	1.75	3.94E-58	57.40	57.45
GSE95962	liver	none	NR2F2	2.93E-59	5.70	2.51	6.62E-58	57.18	57.37
GSM1208738	LoVo	none	ETS2	4.46E-59	4.50	2.17	9.91E-58	57.00	57.11
wgEncodeEH001550	HepG2	none	POLR2A	2.33E-58	3.76	1.91	5.07E-57	56.29	56.36
wgEncodeEH001511	GM12892	none	POLR2A	2.79E-58	3.41	1.77	5.97E-57	56.22	56.28
wgEncodeEH000708	GM12878	none	POLR2A	3.40E-58	3.49	1.80	7.13E-57	56.15	56.20
GSM2758962	HUVEC	none	SIN3A	4.27E-58	3.37	1.75	8.80E-57	56.06	56.11
wgEncodeEH001130	MCF-7	none	POLR2A	1.05E-57	3.29	1.72	2.13E-56	55.67	55.72
GSE101203	A549	none	PHF8	3.40E-57	3.11	1.64	6.77E-56	55.17	55.21
wgEncodeEH002830	HeLa-S3	none	MAX	4.46E-57	4.07	2.02	8.72E-56	55.06	55.14
wgEncodeEH001463	GM12878	none	POLR2A	4.56E-57	3.69	1.88	8.77E-56	55.06	55.12
wgEncodeEH001633	K562	none	POLR2A	1.00E-56	3.58	1.84	1.89E-55	54.72	54.78

GSM1538431	LNCaP	none	CREB1	1.15E-56	4.16	2.06	2.14E-55	54.67	54.76
GSM1574276	PDAC	none	IRF1	1.55E-56	4.82	2.27	2.85E-55	54.55	54.68
GSM1208670	LoVo	none	RFX1	1.75E-56	3.46	1.79	3.15E-55	54.50	54.56
GSE76496.26	HEK293	none	ZNF35	3.60E-56	7.01	2.81	6.38E-55	54.19	54.53
GSM1405082	HeLa	none	AFF4	3.98E-56	3.22	1.69	6.96E-55	54.16	54.20
GSE91830	A549	none	SIN3A	1.82E-55	5.84	2.55	3.09E-54	53.51	53.73
wgEncodeEH000555	K562	none	POLR2A	1.26E-55	3.32	1.73	2.17E-54	53.66	53.71
wgEncodeEH000592	GM12878	none	POLR2A	2.62E-55	3.08	1.62	4.38E-54	53.36	53.40
wgEncodeEH002027	HeLa-S3	none	CHD2	9.07E-55	3.46	1.79	1.50E-53	52.83	52.88
wgEncodeEH001512	GM12892	none	POLR2A	9.84E-55	3.07	1.62	1.60E-53	52.80	52.84
wgEncodeEH000660	K562	IFNa30	POLR2A	1.07E-54	3.10	1.63	1.72E-53	52.76	52.81
wgEncodeEH000710	GM12891	none	POLR2A	1.60E-54	3.26	1.70	2.52E-53	52.60	52.65
wgEncodeEH000704	K562	IFNg30	POLR2A	1.63E-54	3.20	1.68	2.54E-53	52.59	52.64
wgEncodeEH001790	HeLa-S3	none	TBP	1.81E-54	3.18	1.67	2.75E-53	52.56	52.61
wgEncodeEH000702	HUVEC	none	POLR2A	1.78E-54	2.95	1.56	2.73E-53	52.56	52.60
GSE69566.1	HepG2	none	ARID1A	3.67E-54	3.35	1.74	5.50E-53	52.26	52.31
GSM2026051	Kasumi	none	FLI1	2.04E-53	8.02	3.00	2.90E-52	51.54	52.01
GSM1919982	PDAC	none	KLF4	1.42E-53	5.08	2.34	2.07E-52	51.68	51.84
GSM1208730	LoVo	none	E2F7	1.23E-53	3.61	1.85	1.81E-52	51.74	51.81
wgEncodeEH000651	HCT-116	none	POLR2A	1.97E-53	3.04	1.60	2.84E-52	51.55	51.59
wgEncodeEH001848	H1-hESC	none	TBP	3.95E-53	3.33	1.74	5.49E-52	51.26	51.31
wgEncodeEH001478	GM12878	none	TAF1	3.84E-53	3.05	1.61	5.40E-52	51.27	51.31
wgEncodeEH001514	H1-hESC	none	POLR2A	1.51E-52	3.12	1.64	2.07E-51	50.68	50.73

wgEncodeEH000626	GM12878	none	POLR2A	1.71E-52	2.90	1.54	2.32E-51	50.64	50.67
GSE90631.3	HS-ES-2M	none	SMARCC1	6.79E-52	3.91	1.97	9.12E-51	50.04	50.12
GSE90631.4	VA-ES-BJ	none	SMARCC1	8.36E-52	3.73	1.90	1.11E-50	49.95	50.03
wgEncodeEH001517	GM12878	none	POLR2A	1.02E-51	3.01	1.59	1.34E-50	49.87	49.91
GSE105587	GM12878	none	IKZF1	3.78E-51	4.67	2.22	4.90E-50	49.31	49.45
wgEncodeEH000661	K562	IFNa6h	POLR2A	3.98E-51	2.94	1.56	5.11E-50	49.29	49.33
wgEncodeEH000693	MCF-7	none	E2F1	4.87E-51	3.17	1.67	6.18E-50	49.21	49.26
GSM1462476	T47D	1%O2+1mMDMOG 16h	ARNT	6.46E-51	4.21	2.07	8.10E-50	49.09	49.20
GSM1208659	LoVo	none	NFE2L2	9.64E-51	3.12	1.64	1.20E-49	48.92	48.97
GSE91789	MCF-7	none	SIN3A	1.04E-50	3.34	1.74	1.28E-49	48.89	48.95
GSE91749	HEK293	none	ZEB2	2.31E-50	3.74	1.90	2.80E-49	48.55	48.63
wgEncodeEH001522	GM12891	none	POLR2A	4.16E-50	2.91	1.54	5.00E-49	48.30	48.34
wgEncodeEH001838	HeLa-S3	none	POLR2A	6.90E-50	2.84	1.51	8.20E-49	48.09	48.12
wgEncodeEH000563	H1-hESC	none	POLR2A	7.57E-50	2.87	1.52	8.91E-49	48.05	48.09
wgEncodeEH001793	HepG2	none	TBP	8.75E-50	2.88	1.53	1.02E-48	47.99	48.03
wgEncodeEH000705	GM10847	none	POLR2A	2.23E-49	2.80	1.49	2.58E-48	47.59	47.62
wgEncodeEH001513	GM12892	none	TAF1	3.52E-49	2.78	1.47	4.02E-48	47.40	47.43
GSE91416	HEK293	none	ZNF600	4.08E-49	3.96	1.98	4.62E-48	47.34	47.43
GSE91940	GM12878	none	TRIM22	1.40E-48	4.11	2.04	1.56E-47	46.81	46.91
wgEncodeEH001524	GM12891	none	TAF1	1.19E-48	2.80	1.49	1.34E-47	46.87	46.91
wgEncodeEH002085	K562	none	KDM5B	2.05E-48	2.90	1.54	2.26E-47	46.65	46.68
wgEncodeEH000554	HepG2	none	POLR2A	3.56E-48	2.80	1.49	3.88E-47	46.41	46.45

wgEncodeEH002281	SK-N-MC	none	POLR2A	3.89E-48	3.10	1.63	4.19E-47	46.38	46.43
GSM1462475	T47D	1%O2+1mMDMOG 16h	HIF1A	7.17E-48	5.72	2.52	7.52E-47	46.12	46.36
wgEncodeEH002323	A549	DEX_100nM	CREB1	5.37E-48	3.01	1.59	5.74E-47	46.24	46.28
GSE59703.4	hMADS-3	none	KLF11	5.73E-48	3.01	1.59	6.07E-47	46.22	46.26
GSE92093	GM12878	none	TRIM22	1.44E-47	7.18	2.84	1.47E-46	45.83	46.25
wgEncodeEH001581	K562	none	POLR2A	1.08E-47	3.02	1.60	1.12E-46	45.95	45.99
GSE91851	HEK293	none	GLIS2	1.34E-47	3.58	1.84	1.38E-46	45.86	45.93
GSE76496.27	HEK293	none	ZBTB48	4.41E-46	12.55	3.65	4.27E-45	44.37	45.85
wgEncodeEH002330	GM12878	none	RUNX3	2.35E-47	4.18	2.06	2.37E-46	45.62	45.74
GSE94577.3	LTAD	none	AR	6.17E-47	2.90	1.54	6.19E-46	45.21	45.25
wgEncodeEH001605	K562	none	MAX	1.22E-46	4.06	2.02	1.22E-45	44.92	45.02
wgEncodeEH002093	K562	none	RBBP5	1.87E-46	2.77	1.47	1.85E-45	44.73	44.77
GSM1208805	LoVo	none	TP73	2.59E-46	2.82	1.50	2.54E-45	44.60	44.63
wgEncodeEH002029	HepG2	none	MXI1	5.96E-46	3.02	1.59	5.74E-45	44.24	44.29
wgEncodeEH001617	GM12878	none	ELF1	6.66E-46	3.22	1.69	6.35E-45	44.20	44.25
wgEncodeEH001661	HepG2	none	YY1	7.43E-46	2.94	1.56	7.03E-45	44.15	44.20
GSE105837	K562	none	HDAC1	1.18E-45	3.07	1.62	1.11E-44	43.95	44.00
GSE91553	HEK293	none	ZNF341	3.35E-45	3.27	1.71	3.12E-44	43.51	43.57
GSE69566.3	HepG2	none	ARID2	7.46E-45	2.89	1.53	6.89E-44	43.16	43.20
wgEncodeEH001867	K562	IFNg30	MYC	8.61E-45	3.25	1.70	7.88E-44	43.10	43.16
GSE91495	K562	none	CREB3L1	1.05E-44	4.27	2.10	9.42E-44	43.03	43.15
wgEncodeEH001825	K562	none	TBP	9.42E-45	2.83	1.50	8.56E-44	43.07	43.11
wgEncodeEH002816	MCF10A-Er-Src	EtOH_0.01pct	MYC	1.20E-44	3.23	1.69	1.07E-43	42.97	43.03

GSE91480	HEK293	none	PRDM1	1.97E-44	3.03	1.60	1.74E-43	42.76	42.81
GSE96365	HepG2	none	DRAP1	1.95E-44	2.96	1.56	1.73E-43	42.76	42.81
wgEncodeEH002835	MCF10A-Er-Src	4OHTAM_1uM_36hr	E2F4	2.48E-44	2.82	1.49	2.16E-43	42.66	42.70
GSE90631.2	TTC1240	none	SMARCC1	3.35E-44	2.83	1.50	2.90E-43	42.54	42.58
wgEncodeEH002275	A549	EtOH_0.02pct	TAF1	7.38E-44	2.61	1.38	6.36E-43	42.20	42.23
wgEncodeEH001615	SK-N-SH_RA	none	YY1	1.01E-43	2.75	1.46	8.63E-43	42.06	42.10
wgEncodeEH001133	MCF-7	vehicle	MYC	2.83E-43	3.13	1.65	2.40E-42	41.62	41.67
GSE79491	Kasumi	none	NR4A1	3.14E-43	2.84	1.50	2.64E-42	41.58	41.62
wgEncodeEH002271	SK-N-SH	none	SIN3A	4.75E-43	2.69	1.43	3.98E-42	41.40	41.44
wgEncodeEH002308	GM12878	none	PML	8.22E-43	2.68	1.42	6.83E-42	41.17	41.20
wgEncodeEH001774	HUVEC	none	FOS	1.27E-42	2.89	1.53	1.05E-41	40.98	41.02
wgEncodeEH001623	K562	none	YY1	2.64E-42	2.87	1.52	2.16E-41	40.67	40.71
GSE92164	K562	none	SIN3A	3.23E-42	2.69	1.43	2.62E-41	40.58	40.62
wgEncodeEH001471	HepG2	none	SIN3A	3.55E-42	2.68	1.42	2.84E-41	40.55	40.58
wgEncodeEH001798	GM12878	none	TBP	3.51E-42	2.59	1.37	2.84E-41	40.55	40.58
GSE91469	K562	none	ATF1	4.56E-42	3.31	1.73	3.63E-41	40.44	40.51
GSE95909	K562	none	CREM	5.16E-42	3.42	1.78	4.08E-41	40.39	40.46
wgEncodeEH001864	K562	none	CCNT2	9.11E-42	2.76	1.46	7.15E-41	40.15	40.18
GSM1208606	LoVo	none	E2F2	1.35E-41	2.78	1.48	1.05E-40	39.98	40.02
GSM1208802	LoVo	none	TEAD2	1.38E-41	2.67	1.42	1.07E-40	39.97	40.01
GSE92132	HEK293	none	ZBTB11	3.49E-41	2.71	1.44	2.68E-40	39.57	39.61
wgEncodeEH001831	GM12878	none	CHD2	4.01E-41	2.63	1.39	3.07E-40	39.51	39.55
GSM2026052	Kasumi	none	ERG	6.82E-41	5.26	2.40	5.17E-40	39.29	39.52

GSE96124	liver	none	NR2F2	7.25E-41	3.07	1.62	5.47E-40	39.26	39.32
wgEncodeEH000688	HeLa-S3	none	E2F1	7.92E-41	2.52	1.33	5.93E-40	39.23	39.26
wgEncodeEH001523	GM12891	none	POLR2A	9.38E-41	2.56	1.35	6.98E-40	39.16	39.19
wgEncodeEH001475	GM12878	none	POU2F2	1.23E-40	2.70	1.43	9.08E-40	39.04	39.08
wgEncodeEH001863	K562	none	HMGN3	2.57E-40	2.58	1.37	1.89E-39	38.72	38.76
wgEncodeEH002869	K562	none	MAX	7.10E-40	3.05	1.61	5.15E-39	38.29	38.34
wgEncodeEH002862	K562	none	MAZ	7.54E-40	3.18	1.67	5.44E-39	38.26	38.33
wgEncodeEH001584	K562	none	YY1	6.95E-40	2.56	1.35	5.08E-39	38.29	38.33
wgEncodeEH001657	GM12878	none	YY1	9.86E-40	3.09	1.63	7.07E-39	38.15	38.21
wgEncodeEH002800	K562	none	MYC	1.09E-39	2.82	1.49	7.75E-39	38.11	38.15
wgEncodeEH001609	GM12892	none	YY1	1.11E-39	2.59	1.38	7.85E-39	38.11	38.14
wgEncodeEH001815	HeLa-S3	none	CEBPB	1.19E-39	3.13	1.64	8.36E-39	38.08	38.14
GSE91997	HeLa-S3	none	NFE2L2	2.31E-39	2.61	1.38	1.62E-38	37.79	37.83
GSE96461	HepG2	none	CREM	2.48E-39	2.61	1.38	1.71E-38	37.77	37.80
GSM1505756	HUES64	none	SMAD4	2.45E-39	2.52	1.33	1.71E-38	37.77	37.80
GSE96413	GM12878	none	CREM	3.06E-39	2.73	1.45	2.10E-38	37.68	37.72
wgEncodeEH001460	PFSK-1	none	FOXP2	6.16E-39	2.63	1.39	4.21E-38	37.38	37.41
wgEncodeEH003436	MCF-7	serum_stimulated_media	MYC	6.73E-39	2.67	1.42	4.57E-38	37.34	37.38
GSE76496.30	HEK293	none	ZBTB14	8.61E-39	2.77	1.47	5.81E-38	37.24	37.28
GSE105813	K562	none	ZBTB11	9.40E-39	2.92	1.55	6.31E-38	37.20	37.25
wgEncodeEH001858	GM12878	none	POLR2A	1.14E-38	2.45	1.29	7.58E-38	37.12	37.15
wgEncodeEH001818	HeLa-S3	none	RFX5	1.22E-38	2.54	1.35	8.09E-38	37.09	37.12

GSE106040	HEK293	none	ZBTB48	1.58E-38	2.66	1.41	1.04E-37	36.98	37.02
wgEncodeEH002854	H1-hESC	none	SIN3A	2.23E-38	2.75	1.46	1.46E-37	36.84	36.88
GSM717978	LY2	none	NCOA1	2.35E-38	3.07	1.62	1.53E-37	36.81	36.87
wgEncodeEH001866	K562	IFNg6h	IRF1	2.79E-38	2.50	1.32	1.81E-37	36.74	36.77
GSM1831600	SMMC-7721	Transfection by PBX3 Lentivirus	PBX3	3.06E-38	2.79	1.48	1.97E-37	36.70	36.75
wgEncodeEH002087	H1-hESC	none	RBBP5	4.16E-38	2.56	1.36	2.67E-37	36.57	36.61
wgEncodeEH002807	IMR90	none	CEBPB	4.60E-38	2.95	1.56	2.91E-37	36.54	36.59
GSE59703.3	hMADS-3	none	KLF11	4.56E-38	2.50	1.32	2.91E-37	36.54	36.57
GSE76496.16	HEK293	none	ZNF770	6.08E-38	3.44	1.78	3.82E-37	36.42	36.50
GSM1505757	HUES64	none	SMAD4	5.59E-38	2.60	1.38	3.52E-37	36.45	36.49
wgEncodeEH000676	K562	none	E2F6	6.33E-38	2.55	1.35	3.95E-37	36.40	36.44
GSE92176	K562	none	NCOA1	8.10E-38	3.29	1.72	5.00E-37	36.30	36.37
wgEncodeEH000670	K562	IFNg6h	MYC	7.46E-38	2.58	1.36	4.63E-37	36.33	36.37
GSM1208751	LoVo	none	GLI3	8.39E-38	2.50	1.32	5.15E-37	36.29	36.32
wgEncodeEH001619	K562	none	ELF1	9.03E-38	2.88	1.53	5.51E-37	36.26	36.31
wgEncodeEH002855	HeLa-S3	none	MAZ	1.02E-37	2.48	1.31	6.19E-37	36.21	36.24
wgEncodeEH001807	NB4	none	MYC	1.76E-37	2.64	1.40	1.07E-36	35.97	36.01
GSE91411	HEK293	none	ZNF2	2.67E-37	2.66	1.41	1.60E-36	35.80	35.83
wgEncodeEH000719	HUVEC	none	JUN	3.06E-37	2.50	1.32	1.83E-36	35.74	35.77
GSE91406	K562	none	ZEB2	4.19E-37	4.36	2.12	2.47E-36	35.61	35.77
wgEncodeEH001573	GM12891	none	YY1	3.30E-37	2.48	1.31	1.96E-36	35.71	35.74
wgEncodeEH001826	HeLa-S3	none	MXI1	3.46E-37	2.43	1.28	2.05E-36	35.69	35.72

wgEncodeEH002282	A549	EtOH_0.02pct	YY1	5.70E-37	2.41	1.27	3.34E-36	35.48	35.50
wgEncodeEH002305	HepG2	none	CEBPB	6.30E-37	2.40	1.26	3.67E-36	35.44	35.46
GSE96473	HepG2	none	GATAD2A	8.31E-37	2.99	1.58	4.82E-36	35.32	35.37
wgEncodeEH001607	K562	none	SIN3A	1.54E-36	2.41	1.27	8.87E-36	35.05	35.08
GSM1239499	LoVo	none	ELF2	1.59E-36	2.38	1.25	9.12E-36	35.04	35.07
wgEncodeEH001641	HepG2	none	ELF1	1.84E-36	2.55	1.35	1.05E-35	34.98	35.01
GSM1208713	LoVo	none	ATF5	2.27E-36	2.38	1.25	1.29E-35	34.89	34.92
GSE96151	HepG2	none	ZNF792	3.19E-36	2.45	1.29	1.80E-35	34.74	34.77
wgEncodeEH000735	GM12891	TNFa	NFKB1	3.40E-36	2.56	1.35	1.91E-35	34.72	34.75
GSE91805	K562	none	ESRRA	6.00E-36	4.03	2.01	3.36E-35	34.47	34.61
wgEncodeEH001610	H1-hESC	none	TAF7	8.30E-36	2.37	1.25	4.63E-35	34.33	34.36
GSE64233.1	SGBS	TNF	RELA	9.86E-36	3.58	1.84	5.47E-35	34.26	34.36
GSE76496.7	HEK293	none	ZNF341	1.81E-35	2.91	1.54	9.98E-35	34.00	34.05
wgEncodeEH000686	HeLa-S3	none	TFAP2C	1.87E-35	2.48	1.31	1.03E-34	33.99	34.02
wgEncodeEH001598	K562	none	E2F6	2.83E-35	2.66	1.41	1.55E-34	33.81	33.85
wgEncodeEH000629	HCT-116	none	TCF7L2	3.49E-35	2.46	1.30	1.90E-34	33.72	33.75
GSM1208704	LoVo	none	ZNF250	3.58E-35	2.45	1.30	1.94E-34	33.71	33.74
GSE76496.29	HEK293	none	PRDM1	3.78E-35	2.53	1.34	2.04E-34	33.69	33.73
GSM1208754	LoVo	none	HIVEP1	3.96E-35	2.36	1.24	2.12E-34	33.67	33.70
GSM1208731	LoVo	none	E2F8	4.90E-35	2.38	1.25	2.62E-34	33.58	33.61
GSE105662	MCF7	none	ZNF217	6.54E-35	2.49	1.32	3.48E-34	33.46	33.49
GSE96401	HepG2	none	SMAD4	8.34E-35	2.77	1.47	4.41E-34	33.36	33.40
wgEncodeEH002320	K562	none	PML	9.47E-35	2.38	1.25	4.99E-34	33.30	33.33
wgEncodeEH001561	HepG2	none	SP1	1.00E-34	2.46	1.30	5.26E-34	33.28	33.31

GSE91753	K562	none	MIER1	1.59E-34	2.86	1.52	8.28E-34	33.08	33.13
GSE90631.1	G401	none	SMARCC1	1.55E-34	2.45	1.30	8.09E-34	33.09	33.12
GSE95945	HepG2	none	CEBPA	5.61E-34	2.66	1.41	2.90E-33	32.54	32.58
GSE92083	K562	none	ARID1B	6.48E-34	2.58	1.37	3.34E-33	32.48	32.52
wgEncodeEH002328	GM12878	none	EGR1	8.92E-34	2.39	1.26	4.58E-33	32.34	32.37
GSE91455	HEK293	none	ZNF639	1.27E-33	2.31	1.21	6.51E-33	32.19	32.21
wgEncodeEH001567	H1-hESC	none	YY1	1.37E-33	2.38	1.25	6.97E-33	32.16	32.19
wgEncodeEH000685	HeLa-S3	none	TFAP2A	1.82E-33	2.33	1.22	9.23E-33	32.03	32.06
wgEncodeEH001671	HCT-116	none	YY1	1.85E-33	2.33	1.22	9.31E-33	32.03	32.06
wgEncodeEH002263	HepG2	none	REST	2.73E-33	2.31	1.21	1.37E-32	31.86	31.89
wgEncodeEH002852	GM12878	none	MAZ	3.89E-33	2.30	1.20	1.94E-32	31.71	31.74
wgEncodeEH000648	HeLa-S3	none	MYC	4.08E-33	2.28	1.19	2.03E-32	31.69	31.72
wgEncodeEH002831	IMR90	none	CTCF	4.44E-33	2.87	1.52	2.20E-32	31.66	31.71
wgEncodeEH001646	K562	none	EGR1	4.99E-33	2.74	1.45	2.46E-32	31.61	31.66
GSE91709	K562	none	IRF2	5.77E-33	2.31	1.21	2.83E-32	31.55	31.58
wgEncodeEH002094	K562	none	SAP30	6.14E-33	2.28	1.19	3.00E-32	31.52	31.55
GSE92129	K562	none	ZKSCAN1	1.00E-32	2.64	1.40	4.87E-32	31.31	31.36
GSE96359	HepG2	none	BCL6	1.15E-32	2.54	1.34	5.59E-32	31.25	31.29
GSE91730	K562	none	IKZF1	2.21E-32	5.33	2.41	1.05E-31	30.98	31.28
GSE105638	K562	none	RB1	1.18E-32	2.43	1.28	5.70E-32	31.24	31.28
GSE96333	HepG2	none	ETV4	1.28E-32	2.32	1.21	6.14E-32	31.21	31.24
wgEncodeEH002832	IMR90	none	MAFK	1.68E-32	2.30	1.20	8.03E-32	31.10	31.12
GSE92194	HEK293	none	WT1	1.98E-32	2.32	1.22	9.43E-32	31.03	31.05
GSE96254	HepG2	none	TFE3	2.25E-32	2.36	1.24	1.06E-31	30.97	31.00

wgEncodeEH002839	MCF10A-Er-Src	4OHTAM_1uM_36hr	FOS	2.40E-32	2.67	1.42	1.13E-31	30.95	30.99
wgEncodeEH002813	HeLa-S3	none	TCF7L2	2.41E-32	2.30	1.20	1.13E-31	30.95	30.97
wgEncodeEH001521	GM12891	none	SPI1	2.62E-32	2.54	1.34	1.22E-31	30.91	30.95
wgEncodeEH002815	MCF10A-Er-Src	EtOH_0.01pct	FOS	7.30E-32	2.61	1.38	3.40E-31	30.47	30.51
wgEncodeEH002164	K562	none	JUND	9.02E-32	2.66	1.41	4.18E-31	30.38	30.42
wgEncodeEH001816	HeLa-S3	none	GTF2F1	1.04E-31	2.24	1.16	4.80E-31	30.32	30.34
wgEncodeEH001781	HEK293-T-REx	none	ZNF263	1.34E-31	2.43	1.28	6.17E-31	30.21	30.24
wgEncodeEH001496	GM12878	none	SP1	2.47E-31	2.33	1.22	1.13E-30	29.95	29.98
GSM1218850	myotube	none	MYOD1	2.77E-31	2.56	1.36	1.27E-30	29.90	29.94
wgEncodeEH002838	MCF10A-Er-Src	4OHTAM_1uM_4hr	FOS	3.17E-31	2.68	1.42	1.44E-30	29.84	29.89
wgEncodeEH002850	K562	none	UBTF	3.94E-31	2.24	1.17	1.78E-30	29.75	29.77
wgEncodeEH001765	PBDE	none	GATA1	6.08E-31	2.24	1.16	2.74E-30	29.56	29.59
wgEncodeEH000983	HCPEpiC	none	CTCF	7.80E-31	3.01	1.59	3.50E-30	29.46	29.52
wgEncodeEH002844	HeLa-S3	none	RCOR1	1.03E-30	2.24	1.16	4.63E-30	29.33	29.36
GSE92035	HEK293	none	ZSCAN20	1.05E-30	2.21	1.14	4.66E-30	29.33	29.36
wgEncodeEH000978	AG09319	none	CTCF	1.17E-30	2.81	1.49	5.19E-30	29.28	29.34
GSE92067	HEK293	none	HIC1	1.24E-30	2.26	1.17	5.47E-30	29.26	29.29
GSE96121	HepG2	none	CREB1	1.41E-30	2.21	1.14	6.20E-30	29.21	29.23
wgEncodeEH002312	A549	EtOH_0.02pct	TCF12	1.46E-30	2.22	1.15	6.42E-30	29.19	29.22
wgEncodeEH001806	NB4	none	MAX	2.25E-30	2.55	1.35	9.82E-30	29.01	29.05
wgEncodeEH001547	SK-N-SH	none	REST	2.82E-30	2.25	1.17	1.23E-29	28.91	28.94

wgEncodeEH000053	K562	none	POLR2A	2.96E-30	2.20	1.13	1.28E-29	28.89	28.92
wgEncodeEH000405	HRE	none	CTCF	3.99E-30	2.61	1.39	1.72E-29	28.76	28.81
wgEncodeEH002306	GM12878	none	ATF2	4.18E-30	2.23	1.16	1.80E-29	28.74	28.77
wgEncodeEH002837	MCF10A-Er-Src	4OHTAM_1uM_12hr	FOS	4.80E-30	2.70	1.43	2.06E-29	28.69	28.74
GSE91470	K562	none	HES1	5.39E-30	2.53	1.34	2.30E-29	28.64	28.68
wgEncodeEH001480	GM12878	none	EBF1	5.69E-30	2.35	1.23	2.42E-29	28.62	28.65
wgEncodeEH001555	A549	DEX_100nM	NR3C1	7.00E-30	2.19	1.13	2.97E-29	28.53	28.55
wgEncodeEH001482	K562	none	SPI1	7.38E-30	2.29	1.20	3.12E-29	28.51	28.54
GSE91745	GM12878	none	TARDBP	7.74E-30	2.20	1.14	3.26E-29	28.49	28.51
wgEncodeEH001620	K562	none	ZBTB7A	8.43E-30	2.28	1.19	3.54E-29	28.45	28.48
wgEncodeEH000986	HPAF	none	CTCF	1.01E-29	2.86	1.52	4.22E-29	28.37	28.44
GSE91408	K562	none	ID3	1.08E-29	2.33	1.22	4.49E-29	28.35	28.38
wgEncodeEH002529	GM12878	none	FOXM1	1.47E-29	2.22	1.15	6.11E-29	28.21	28.24
GSM1208605	LoVo	none	DBP	1.56E-29	2.19	1.13	6.46E-29	28.19	28.22
wgEncodeEH001489	GM12878	none	PAX5	1.59E-29	2.30	1.20	6.55E-29	28.18	28.21
wgEncodeEH002840	MCF10A-Er-Src	4OHTAM_1uM_4hr	MYC	1.73E-29	2.25	1.17	7.06E-29	28.15	28.18
wgEncodeEH001546	GM12892	none	PAX5	1.72E-29	2.17	1.12	7.06E-29	28.15	28.18
wgEncodeEH001548	HepG2	none	GABPA	1.78E-29	2.18	1.12	7.27E-29	28.14	28.16
wgEncodeEH000979	AG10803	none	CTCF	1.92E-29	2.66	1.41	7.80E-29	28.11	28.16
wgEncodeEH001769	MCF10A-Er-Src	4OHTAM_1uM_36hr	STAT3	2.85E-29	2.32	1.21	1.16E-28	27.94	27.97
GSE64233.2	SGBS	none	RELA	2.97E-29	2.20	1.13	1.19E-28	27.92	27.95

wgEncodeEH001580	K562	none	ETS1	2.96E-29	2.17	1.12	1.19E-28	27.92	27.95
wgEncodeEH001894	HCM	none	CTCF	3.38E-29	2.74	1.46	1.34E-28	27.87	27.93
wgEncodeEH000410	HUVEC	none	CTCF	3.30E-29	2.54	1.34	1.32E-28	27.88	27.92
wgEncodeEH002058	NHDF-neo	none	CTCF	3.44E-29	2.63	1.39	1.36E-28	27.87	27.91
GSM1961563	TH1	none	AFF4	3.37E-29	2.16	1.11	1.34E-28	27.87	27.90
wgEncodeEH000976	AG04450	none	CTCF	3.75E-29	2.64	1.40	1.48E-28	27.83	27.88
wgEncodeEH000985	HMF	none	CTCF	4.08E-29	2.73	1.45	1.61E-28	27.79	27.85
wgEncodeEH000419	HMEC	none	CTCF	4.49E-29	2.82	1.49	1.75E-28	27.76	27.82
wgEncodeEH001495	GM12878	none	PAX5	4.31E-29	2.20	1.14	1.69E-28	27.77	27.80
wgEncodeEH001588	T-47D	DMSO_0.02pct	FOXA1	5.91E-29	2.28	1.19	2.30E-28	27.64	27.67
wgEncodeEH000982	HCFaa	none	CTCF	6.16E-29	2.53	1.34	2.39E-28	27.62	27.66
wgEncodeEH001509	A549	DEX_100nM	USF1	6.65E-29	2.15	1.11	2.57E-28	27.59	27.61
wgEncodeEH001630	HepG2	none	FOXA1	8.47E-29	2.31	1.21	3.27E-28	27.49	27.52
wgEncodeEH001616	SK-N-SH_RA	none	EP300	1.01E-28	2.21	1.14	3.88E-28	27.41	27.44
wgEncodeEH002056	HVMF	none	CTCF	1.20E-28	2.60	1.38	4.59E-28	27.34	27.39
wgEncodeEH002814	K562	none	RCOR1	1.34E-28	2.34	1.23	5.11E-28	27.29	27.32
wgEncodeEH001470	HepG2	none	JUND	1.37E-28	2.21	1.15	5.22E-28	27.28	27.31
wgEncodeEH000980	AoAF	none	CTCF	1.49E-28	2.60	1.38	5.67E-28	27.25	27.29
wgEncodeEH001485	GM12878	none	TCF12	1.52E-28	2.20	1.14	5.75E-28	27.24	27.27
wgEncodeEH000110	HSMM	none	CTCF	1.65E-28	2.64	1.40	6.22E-28	27.21	27.26

wgEncodeEH000075	HMEC	none	CTCF	1.68E-28	2.45	1.29	6.32E-28	27.20	27.24
wgEncodeEH000736	GM15510	TNFa	NFKB1	1.76E-28	2.14	1.10	6.60E-28	27.18	27.20
wgEncodeEH000987	HPF	none	CTCF	1.97E-28	2.58	1.37	7.33E-28	27.14	27.18
wgEncodeEH002026	GM12878	none	MXI1	1.96E-28	2.15	1.10	7.33E-28	27.14	27.16
wgEncodeEH002265	PANC-1	none	POLR2A	2.06E-28	2.14	1.10	7.64E-28	27.12	27.14
wgEncodeEH001789	HeLa-S3	none	RAD21	4.27E-28	2.42	1.28	1.58E-27	26.80	26.84
GSM1816455	THP-1	none	ARNTL	4.50E-28	2.16	1.11	1.66E-27	26.78	26.80
wgEncodeEH002333	K562	none	TEAD4	6.63E-28	2.24	1.16	2.44E-27	26.61	26.64
GSM1218849	myoblast	none	MYOD1	6.88E-28	2.35	1.24	2.52E-27	26.60	26.63
wgEncodeEH000406	NHEK	none	CTCF	7.14E-28	2.48	1.31	2.61E-27	26.58	26.63
wgEncodeEH000975	AG04449	none	CTCF	7.52E-28	2.68	1.42	2.73E-27	26.56	26.62
wgEncodeEH001594	HepG2	none	FOXA1	7.17E-28	2.31	1.21	2.61E-27	26.58	26.62
wgEncodeEH001012	HeLa-S3	none	CTCF	7.80E-28	2.59	1.37	2.82E-27	26.55	26.60
wgEncodeEH001612	HepG2	none	EP300	7.83E-28	2.16	1.11	2.82E-27	26.55	26.57
wgEncodeEH000984	HEEpiC	none	CTCF	9.67E-28	2.55	1.35	3.48E-27	26.46	26.50
wgEncodeEH001893	HAc	none	CTCF	1.10E-27	2.54	1.35	3.96E-27	26.40	26.45
wgEncodeEH002810	IMR90	none	RAD21	1.16E-27	2.37	1.24	4.14E-27	26.38	26.42
wgEncodeEH002818	A549	none	CEBPB	1.15E-27	2.20	1.14	4.12E-27	26.38	26.41
wgEncodeEH000403	BJ	none	CTCF	1.52E-27	2.55	1.35	5.41E-27	26.27	26.31
wgEncodeEH000745	HeLa-S3	none	JUND	1.54E-27	2.20	1.14	5.44E-27	26.26	26.29

wgEncodeEH001650	HepG2	none	FOXA2	1.64E-27	2.23	1.16	5.78E-27	26.24	26.27
wgEncodeEH000977	AG09309	none	CTCF	2.70E-27	2.49	1.31	9.49E-27	26.02	26.07
wgEncodeEH002059	RPTEC	none	CTCF	2.82E-27	2.67	1.42	9.90E-27	26.00	26.06
wgEncodeEH000989	HA-sp	none	CTCF	2.86E-27	2.53	1.34	1.00E-26	26.00	26.04
GSE94782.1	NGP	none	MYCN	3.43E-26	8.36	3.06	1.11E-25	24.95	26.02
GSE72266.1	Th1	none	MYB	2.98E-27	2.12	1.08	1.04E-26	25.98	26.01
wgEncodeEH001026	NH-A	none	CTCF	4.18E-27	2.37	1.24	1.45E-26	25.84	25.87
GSE91614	K562	none	PBX2	4.41E-27	2.16	1.11	1.53E-26	25.82	25.84
wgEncodeEH000063	NHEK	none	CTCF	5.34E-27	2.51	1.33	1.85E-26	25.73	25.78
GSE95940	HepG2	none	PPARG	6.74E-27	2.15	1.11	2.31E-26	25.64	25.66
GSM1410763	LNCaP	DHT 10 nM 24H	CTBP2	6.72E-27	2.13	1.09	2.31E-26	25.64	25.66
wgEncodeEH001902	WI-38	none	CTCF	7.10E-27	2.28	1.19	2.43E-26	25.61	25.65
wgEncodeEH001839	HeLa-S3	none	SMC3	7.22E-27	2.38	1.25	2.47E-26	25.61	25.65
wgEncodeEH001476	GM12878	none	SPI1	7.70E-27	2.27	1.18	2.61E-26	25.58	25.61
wgEncodeEH002310	A549	EtOH_0.02pct	FOSL2	7.69E-27	2.14	1.10	2.61E-26	25.58	25.61
wgEncodeEH000120	NHLF	none	CTCF	9.71E-27	2.43	1.28	3.27E-26	25.49	25.53
wgEncodeEH001801	HepG2	none	JUND	9.37E-27	2.15	1.11	3.17E-26	25.50	25.53
GSE96344	liver	none	FOXA1	9.38E-27	2.09	1.06	3.17E-26	25.50	25.52
wgEncodeEH002547	HFF	none	CTCF	1.32E-26	2.32	1.21	4.43E-26	25.35	25.39
wgEncodeEH002402	Dnd41	none	CTCF	1.37E-26	2.54	1.34	4.59E-26	25.34	25.38
wgEncodeEH001565	GM12878	none	MEF2A	2.14E-26	2.08	1.05	7.15E-26	25.15	25.17

GSM1218851	Rhabdomyosarcoma	none	MYOD1	2.49E-26	2.22	1.15	8.24E-26	25.08	25.11
GSE59703.1	hMADS-3	none	PPARG	2.45E-26	2.08	1.06	8.15E-26	25.09	25.11
wgEncodeEH002319	HepG2	none	NFIC	2.49E-26	2.08	1.05	8.24E-26	25.08	25.11
wgEncodeEH001211	K562	none	JUND	2.53E-26	2.17	1.12	8.35E-26	25.08	25.11
wgEncodeEH002090	K562	none	HDAC1	2.59E-26	2.08	1.05	8.52E-26	25.07	25.09
wgEncodeEH001501	HepG2	none	FOSL2	2.63E-26	2.14	1.10	8.61E-26	25.07	25.09
wgEncodeEH000392	GM06990	none	CTCF	2.84E-26	2.47	1.30	9.29E-26	25.03	25.08
wgEncodeEH001851	GM12878	none	CTCF	3.17E-26	2.59	1.38	1.03E-25	24.99	25.04
wgEncodeEH001842	HepG2	none	MAFK	3.23E-26	2.10	1.07	1.05E-25	24.98	25.00
GSE105218	HepG2	none	FOXA2	3.65E-26	2.21	1.14	1.18E-25	24.93	24.96
GSE69566.2	HepG2	none	ARID1B	3.75E-26	2.07	1.05	1.21E-25	24.92	24.94
wgEncodeEH001758	HUVEC	none	GATA2	4.27E-26	2.07	1.05	1.37E-25	24.86	24.89
wgEncodeEH001614	SK-N-SH_RA	none	RAD21	5.94E-26	2.52	1.34	1.90E-25	24.72	24.77
wgEncodeEH001000	HSMMtube	none	CTCF	6.53E-26	2.45	1.29	2.09E-25	24.68	24.72
wgEncodeEH000541	HeLa-S3	none	CTCF	6.79E-26	2.55	1.35	2.15E-25	24.67	24.72
wgEncodeEH002548	NHLF	none	CTCF	6.68E-26	2.30	1.20	2.13E-25	24.67	24.71
wgEncodeEH000690	GM12878	TNFa	NFKB1	6.72E-26	2.06	1.04	2.14E-25	24.67	24.69
wgEncodeEH000614	HeLa-S3	IFNg30	STAT1	6.95E-26	2.06	1.05	2.20E-25	24.66	24.68
wgEncodeEH000437	SAEC	none	CTCF	7.48E-26	2.42	1.28	2.36E-25	24.63	24.67
GSE105487	Liver	none	FOXA2	7.56E-26	2.06	1.05	2.38E-25	24.62	24.65
GSE97661.6	HepG2	none	KLF10	7.93E-26	2.06	1.04	2.49E-25	24.60	24.63

wgEncodeEH000981	HBMEC	none	CTCF	8.61E-26	2.63	1.39	2.69E-25	24.57	24.62
wgEncodeEH001529	H1-hESC	none	SP1	8.55E-26	2.08	1.05	2.67E-25	24.57	24.60
wgEncodeEH000449	GM12872	none	CTCF	9.86E-26	2.47	1.30	3.07E-25	24.51	24.56
wgEncodeEH001779	HEK293	none	TRIM28	1.15E-25	2.05	1.04	3.58E-25	24.45	24.47
wgEncodeEH002797	K562	none	CTCF	1.24E-25	2.55	1.35	3.84E-25	24.42	24.47
wgEncodeEH001871	MCF10A-Er-Src	EtOH_0.01pct_4hr	STAT3	1.33E-25	2.15	1.10	4.12E-25	24.39	24.41
wgEncodeEH000029	GM12878	none	CTCF	1.50E-25	2.43	1.28	4.60E-25	24.34	24.38
GSE105440	liver	none	HNF4G	1.66E-25	2.06	1.04	5.09E-25	24.29	24.32
wgEncodeEH001857	K562	none	BHLHE40	1.86E-25	2.15	1.10	5.71E-25	24.24	24.27
wgEncodeEH001621	SK-N-SH_RA	none	USF1	1.92E-25	2.11	1.07	5.85E-25	24.23	24.26
wgEncodeEH002343	GM12878	none	NFIC	1.93E-25	2.07	1.05	5.88E-25	24.23	24.25
wgEncodeEH001048	NHDF-Ad	none	CTCF	2.25E-25	2.45	1.29	6.81E-25	24.17	24.21
wgEncodeEH000404	Caco-2	none	CTCF	2.34E-25	2.41	1.27	7.04E-25	24.15	24.19
wgEncodeEH001604	K562	none	GABPA	2.23E-25	2.08	1.06	6.77E-25	24.17	24.19
wgEncodeEH002865	K562	none	ATF1	2.26E-25	2.05	1.04	6.84E-25	24.17	24.19
wgEncodeEH002088	K562	none	CHD1	2.34E-25	2.06	1.04	7.04E-25	24.15	24.18
GSE92077	GM12878	none	MYB	2.65E-25	2.05	1.03	7.95E-25	24.10	24.12
GSM1505700	HUES64	none	NANOG	4.00E-25	2.27	1.18	1.20E-24	23.92	23.96
wgEncodeEH000398	HeLa-S3	none	CTCF	4.08E-25	2.28	1.19	1.22E-24	23.92	23.95
wgEncodeEH001821	K562	none	CEBPB	4.47E-25	2.14	1.10	1.33E-24	23.88	23.90

wgEncodeEH001640	GM12878	none	RAD21	5.74E-25	2.26	1.18	1.70E-24	23.77	23.80
wgEncodeEH000746	HeLa-S3	none	JUN	5.85E-25	2.04	1.03	1.73E-24	23.76	23.78
wgEncodeEH000722	HeLa-S3	none	SMARCB1	6.27E-25	2.05	1.03	1.85E-24	23.73	23.76
GSE91565	IMR90	none	NFE2L2	6.34E-25	2.03	1.02	1.87E-24	23.73	23.75
wgEncodeEH000054	HUVEC	none	CTCF	6.79E-25	2.29	1.20	1.99E-24	23.70	23.74
wgEncodeEH001829	HepG2	none	CEBPB	6.89E-25	2.19	1.13	2.01E-24	23.70	23.73
wgEncodeEH001520	GM12891	none	POU2F2	6.77E-25	2.04	1.03	1.99E-24	23.70	23.72
wgEncodeEH000571	GM19239	none	CTCF	7.22E-25	2.35	1.23	2.11E-24	23.68	23.72
GSE76496.17	HEK293	none	ZNF260	7.50E-25	2.03	1.02	2.18E-24	23.66	23.68
wgEncodeEH000042	K562	none	CTCF	8.30E-25	2.45	1.29	2.41E-24	23.62	23.66
wgEncodeEH000448	GM12865	none	CTCF	9.60E-25	2.37	1.25	2.78E-24	23.56	23.60
wgEncodeEH001131	MCF-7	estrogen	CTCF	1.27E-24	2.53	1.34	3.66E-24	23.44	23.49
wgEncodeEH000671	K562	none	E2F4	1.23E-24	2.02	1.02	3.55E-24	23.45	23.47
wgEncodeEH001853	GM12878	none	ZNF143	1.39E-24	2.11	1.08	3.98E-24	23.40	23.43
GSM831040	H1 ES	none	SAP30	1.38E-24	2.02	1.02	3.95E-24	23.40	23.43
wgEncodeEH001770	SH-SY5Y	none	GATA2	1.40E-24	2.03	1.02	3.99E-24	23.40	23.42
wgEncodeEH002057	MCF-7	none	CTCF	1.54E-24	2.43	1.28	4.37E-24	23.36	23.40
wgEncodeEH002025	GM12878	none	BHLHE40	1.47E-24	2.02	1.02	4.19E-24	23.38	23.40
wgEncodeEH001464	GM12878	none	SRF	1.50E-24	2.03	1.02	4.26E-24	23.37	23.39
GSE59703.2	hMADS-3	none	PPARG	1.77E-24	2.02	1.01	4.99E-24	23.30	23.32
wgEncodeEH002804	K562	none	MAFF	1.77E-24	2.02	1.01	5.00E-24	23.30	23.32

wgEncodeEH001507	A549	DEX_100nM	CTCF	2.31E-24	2.25	1.17	6.48E-24	23.19	23.22
GSE106046	GM12878	none	RB1	2.25E-24	2.01	1.01	6.33E-24	23.20	23.22
wgEncodeEH001844	K562	none	MAFK	2.30E-24	2.01	1.01	6.46E-24	23.19	23.21
wgEncodeEH000536	K562	none	MYC	2.80E-24	2.01	1.01	7.83E-24	23.11	23.13
GSM2480802	LNCaP	none	AR	3.42E-24	2.03	1.02	9.52E-24	23.02	23.04
wgEncodeEH000447	GM12864	none	CTCF	3.59E-24	2.38	1.25	9.97E-24	23.00	23.04
wgEncodeEH002055	HCT-116	none	CTCF	3.68E-24	2.35	1.23	1.02E-23	22.99	23.03
wgEncodeEH001479	GM12878	none	BATF	4.00E-24	2.04	1.03	1.11E-23	22.96	22.98
wgEncodeEH001662	K562	none	ATF3	4.12E-24	2.04	1.03	1.14E-23	22.94	22.97
wgEncodeEH001841	HepG2	none	MAFF	5.33E-24	2.00	1.00	1.47E-23	22.83	22.86
wgEncodeEH001554	U87	none	REST	5.55E-24	2.00	1.00	1.52E-23	22.82	22.84
wgEncodeEH000452	GM12875	none	CTCF	6.59E-24	2.27	1.18	1.81E-23	22.74	22.78
wgEncodeEH001824	K562	none	TAL1	6.93E-24	2.02	1.01	1.90E-23	22.72	22.75
wgEncodeEH002834	K562	none	EP300	7.25E-24	2.05	1.04	1.98E-23	22.70	22.73
wgEncodeEH001890	A549	none	CTCF	7.82E-24	2.31	1.21	2.13E-23	22.67	22.71
wgEncodeEH000450	GM12873	none	CTCF	8.46E-24	2.43	1.28	2.30E-23	22.64	22.68
GSE67555	neonatal foreskin	none	TFAP2A	8.98E-24	2.00	1.00	2.43E-23	22.61	22.64
wgEncodeEH001753	HeLa-S3	none	ELK4	9.23E-24	2.03	1.02	2.50E-23	22.60	22.63
wgEncodeEH000988	HRPEpiC	none	CTCF	1.02E-23	2.40	1.26	2.75E-23	22.56	22.60
wgEncodeEH001814	HeLa-S3	none	BRCA1	9.91E-24	2.00	1.00	2.67E-23	22.57	22.60

wgEncodeEH000439	SK-N-SH_RA	none	CTCF	1.09E-23	2.37	1.24	2.93E-23	22.53	22.57
wgEncodeEH001041	Osteobl	none	CTCF	1.25E-23	2.40	1.26	3.36E-23	22.47	22.52
wgEncodeEH000551	HUVEC	none	CTCF	1.34E-23	2.24	1.16	3.59E-23	22.45	22.48
wgEncodeEH001891	BE2_C	none	CTCF	1.37E-23	2.37	1.24	3.66E-23	22.44	22.48
wgEncodeEH001868	MCF10A-Er-Src	EtOH_0.01pct_12hr	STAT3	1.45E-23	2.06	1.04	3.86E-23	22.41	22.44
GSE92062	K562	none	NCOR1	1.72E-23	2.01	1.01	4.57E-23	22.34	22.36
wgEncodeEH002382	K562	none	NR2F2	1.76E-23	1.99	0.99	4.65E-23	22.33	22.35
wgEncodeEH002318	HepG2	none	MBD4	2.04E-23	2.12	1.08	5.38E-23	22.27	22.30
wgEncodeEH000535	K562	none	CTCF	2.19E-23	2.39	1.26	5.77E-23	22.24	22.28
wgEncodeEH001132	MCF-7	vehicle	CTCF	2.45E-23	2.67	1.42	6.43E-23	22.19	22.25
wgEncodeEH001895	HFF-Myc	none	CTCF	2.53E-23	2.27	1.18	6.63E-23	22.18	22.21
wgEncodeEH002344	HepG2	none	MYBL2	2.54E-23	2.00	1.00	6.63E-23	22.18	22.20
GSE96455	GM12878	none	JUNB	2.91E-23	1.99	0.99	7.59E-23	22.12	22.14
wgEncodeEH000768	HUVEC	none	MAX	3.41E-23	1.98	0.98	8.88E-23	22.05	22.07
wgEncodeEH001639	T-47D	DMSO_0.02pct	GATA3	3.55E-23	2.01	1.00	9.22E-23	22.04	22.06
GSE91471	K562	none	CREB3	3.55E-23	1.98	0.99	9.22E-23	22.04	22.06
wgEncodeEH000080	HepG2	none	CTCF	4.01E-23	2.27	1.18	1.04E-22	21.98	22.02
wgEncodeEH000061	HUVEC	none	POLR2A	3.99E-23	2.08	1.06	1.03E-22	21.99	22.01
wgEncodeEH002065	GM12878	none	NFYB	4.10E-23	1.97	0.98	1.06E-22	21.98	22.00
wgEncodeEH001461	SK-N-MC	none	FOXP2	4.54E-23	1.97	0.98	1.17E-22	21.93	21.95
GSE92157	K562	none	NCOR1	5.10E-23	1.98	0.99	1.31E-22	21.88	21.91

wgEncodeEH000738	GM18951	TNFa	NFKB1	5.81E-23	1.97	0.98	1.49E-22	21.83	21.85
wgEncodeEH002304	HepG2	none	CEBPB	6.10E-23	1.97	0.98	1.56E-22	21.81	21.83
wgEncodeEH002798	K562	IFNa6h	IRF1	7.65E-23	2.01	1.00	1.95E-22	21.71	21.73
wgEncodeEH001776	U2OS	none	TRIM28	9.37E-23	1.96	0.97	2.38E-22	21.62	21.64
GSE76496.21	HEK293	none	ZNF134	1.25E-22	1.98	0.98	3.16E-22	21.50	21.52
wgEncodeEH002836	MCF10A-Er-Src	4OHTAM_1uM_12hr	STAT3	1.32E-22	2.02	1.02	3.33E-22	21.48	21.50
wgEncodeEH002279	K562	none	CTCF	1.57E-22	2.27	1.18	3.98E-22	21.40	21.44
wgEncodeEH001613	SK-N-SH_RA	none	CTCF	1.60E-22	2.18	1.13	4.04E-22	21.39	21.43
GSM1278507	primary epidermal keratinocytes	none	MAFB	1.67E-22	2.25	1.17	4.18E-22	21.38	21.42
GSM1574275	PDAC	none	HNF1B	1.64E-22	1.96	0.97	4.11E-22	21.39	21.41
wgEncodeEH001579	H1-hESC	none	JUND	1.99E-22	1.96	0.97	4.99E-22	21.30	21.32
GSE76496.24	HEK293	none	ZNF41	2.04E-22	2.01	1.01	5.10E-22	21.29	21.32
wgEncodeEH002858	HepG2	none	ARID3A	2.35E-22	1.95	0.97	5.86E-22	21.23	21.25
wgEncodeEH000669	K562	IFNa6h	MYC	2.43E-22	1.95	0.96	6.05E-22	21.22	21.24
wgEncodeEH002346	K562	none	CEBPB	2.63E-22	1.97	0.98	6.54E-22	21.18	21.21
wgEncodeEH000532	GM12878	none	CTCF	3.07E-22	2.28	1.19	7.59E-22	21.12	21.16
GSE91431	K562	none	RFX1	3.00E-22	2.00	1.00	7.44E-22	21.13	21.15
GSE96114	liver	none	FOXA1	3.13E-22	1.98	0.99	7.71E-22	21.11	21.14
wgEncodeEH002820	A549	none	MAX	3.49E-22	1.94	0.96	8.58E-22	21.07	21.09
wgEncodeEH002799	K562	IFNg30	IRF1	4.34E-22	1.97	0.98	1.07E-21	20.97	20.99
GSM1624706	MDA-MB-231	none	TP53	4.39E-22	1.95	0.96	1.08E-21	20.97	20.99

wgEncodeEH001820	HeLa-S3	none	EP300	4.50E-22	1.94	0.96	1.10E-21	20.96	20.98
wgEncodeEH001539	A549	EtOH_0.02pct	USF1	4.50E-22	1.94	0.96	1.10E-21	20.96	20.98
wgEncodeEH000545	HepG2	none	MYC	4.68E-22	2.06	1.04	1.14E-21	20.94	20.97
GSM1084800	OCI-Ly7	none	BACH2	7.47E-22	1.95	0.96	1.81E-21	20.74	20.76
wgEncodeEH002030	K562	none	ZNF143	7.77E-22	2.07	1.05	1.88E-21	20.72	20.75
wgEncodeEH001850	HepG2	none	MAFK	7.80E-22	1.93	0.95	1.89E-21	20.72	20.75
GSE105745	K562	none	ZNF197	8.90E-22	1.94	0.96	2.15E-21	20.67	20.69
GSE91490	K562	none	TAL1	9.06E-22	1.94	0.95	2.18E-21	20.66	20.68
wgEncodeEH000394	GM12878	none	CTCF	9.52E-22	2.17	1.12	2.29E-21	20.64	20.67
wgEncodeEH001516	HepG2	none	CTCF	9.79E-22	2.29	1.20	2.35E-21	20.63	20.67
wgEncodeEH001608	HepG2	none	RAD21	1.14E-21	2.25	1.17	2.71E-21	20.57	20.60
GSE76496.15	HEK293	none	ZNF449	1.10E-21	1.96	0.97	2.64E-21	20.58	20.60
wgEncodeEH001135	Glioblasts	none	CTCF	1.19E-21	2.38	1.25	2.84E-21	20.55	20.59
wgEncodeEH001210	K562	none	JUNB	1.13E-21	1.93	0.95	2.71E-21	20.57	20.59
wgEncodeEH000451	GM12874	none	CTCF	1.32E-21	2.14	1.10	3.13E-21	20.50	20.54
GSM1239462	LoVo	none	HOXA6	1.44E-21	1.95	0.96	3.41E-21	20.47	20.49
wgEncodeEH001860	HepG2	none	POLR2A	1.50E-21	2.21	1.14	3.55E-21	20.45	20.49
GSM1642517	MCF-7	none	MTA3	1.66E-21	1.93	0.95	3.91E-21	20.41	20.43
GSE91448	MCF-7	none	RFX1	2.03E-21	1.96	0.97	4.78E-21	20.32	20.34
wgEncodeEH000399	K562	none	CTCF	2.20E-21	2.17	1.12	5.17E-21	20.29	20.32
wgEncodeEH002329	GM12878	none	MTA3	2.32E-21	1.92	0.94	5.43E-21	20.26	20.29
GSE76496.1	HEK293	none	ZNF281	2.58E-21	1.96	0.97	6.05E-21	20.22	20.24

GSE91687	MCF-7	none	MTA1	2.89E-21	1.93	0.95	6.75E-21	20.17	20.19
GSE92075	K562	none	SREBF1	3.33E-21	1.91	0.94	7.77E-21	20.11	20.13
wgEncodeEH001794	HepG2	none	JUN	3.44E-21	1.95	0.96	7.99E-21	20.10	20.12
GSE91809	HepG2	none	NFE2L2	3.56E-21	1.91	0.93	8.26E-21	20.08	20.10
wgEncodeEH002292	A549	EtOH_0.02pct	EP300	3.97E-21	1.91	0.94	9.20E-21	20.04	20.06
wgEncodeEH001472	HepG2	none	USF1	4.15E-21	1.94	0.95	9.59E-21	20.02	20.04
wgEncodeEH002796	HepG2	none	MAX	4.45E-21	1.92	0.94	1.03E-20	19.99	20.01
GSM1505751	HUES64	none	SMAD4	6.25E-21	1.98	0.99	1.44E-20	19.84	19.87
wgEncodeEH001822	K562	none	CHD2	6.27E-21	1.92	0.94	1.44E-20	19.84	19.86
wgEncodeEH002067	HeLa-S3	none	NFYB	6.86E-21	1.95	0.96	1.57E-20	19.80	19.83
wgEncodeEH000749	GM12878	none	RAD21	8.60E-21	2.02	1.01	1.97E-20	19.71	19.73
wgEncodeEH000570	GM19238	none	CTCF	9.39E-21	2.24	1.17	2.14E-20	19.67	19.71
GSE95936	HepG2	none	MLX	1.17E-20	1.90	0.92	2.66E-20	19.57	19.60
wgEncodeEH001819	HeLa-S3	none	USF2	1.31E-20	1.90	0.92	2.98E-20	19.53	19.55
GSE73725	h9 hESC	none	NANOG	1.31E-20	1.91	0.93	2.98E-20	19.53	19.55
GSE38567.2	PBMCs	2.5h 500 U/ml of IFN-a2b	STAT4	1.42E-20	1.90	0.93	3.22E-20	19.49	19.51
wgEncodeEH000750	HepG2	forskolin	CEBPB	1.56E-20	1.90	0.93	3.52E-20	19.45	19.47
wgEncodeEH000684	K562	none	YY1	1.59E-20	1.98	0.98	3.60E-20	19.44	19.47
wgEncodeEH002848	K562	none	TBL1XR1	1.62E-20	1.91	0.93	3.66E-20	19.44	19.46
wgEncodeEH001484	GM12878	none	IRF4	1.65E-20	1.89	0.92	3.71E-20	19.43	19.45
GSE96385	HepG2	none	HNF1A	2.03E-20	1.91	0.93	4.55E-20	19.34	19.36
wgEncodeEH001504	HeLa-S3	none	GABPA	2.09E-20	1.90	0.93	4.68E-20	19.33	19.35

wgEncodeEH002294	A549	EtOH_0.02pct	GABPA	2.59E-20	1.89	0.92	5.80E-20	19.24	19.26
wgEncodeEH001508	A549	EtOH_0.02pct	CTCF	2.70E-20	2.05	1.04	6.03E-20	19.22	19.25
GSE97661.8	HepG2	none	NFATC3	2.96E-20	1.93	0.95	6.60E-20	19.18	19.20
wgEncodeEH001618	HepG2	none	HDAC2	3.00E-20	1.89	0.92	6.66E-20	19.18	19.20
GSE76496.4	HEK293	none	ZNF350	3.28E-20	1.90	0.93	7.28E-20	19.14	19.16
GSE72266.2	Th2	none	MYB	3.88E-20	1.90	0.92	8.60E-20	19.07	19.09
wgEncodeEH001578	K562	none	SP1	3.90E-20	1.90	0.92	8.62E-20	19.06	19.09
wgEncodeEH002078	A549	none	CTCF	5.08E-20	2.25	1.17	1.12E-19	18.95	18.99
GSE91537	HepG2	none	ZKSCAN1	5.14E-20	1.89	0.92	1.13E-19	18.95	18.97
wgEncodeEH000401	HepG2	none	CTCF	6.99E-20	2.13	1.09	1.53E-19	18.81	18.85
GSE56674.2	keratyinocytes	none	TP63	6.89E-20	1.87	0.91	1.51E-19	18.82	18.84
wgEncodeEH002071	PANC-1	none	TCF7L2	7.36E-20	1.88	0.91	1.61E-19	18.79	18.81
wgEncodeEH000600	ProgFib	none	CTCF	8.24E-20	2.10	1.07	1.80E-19	18.74	18.78
wgEncodeEH002802	H1-hESC	none	ZNF143	8.31E-20	1.99	0.99	1.81E-19	18.74	18.77
wgEncodeEH002856	HeLa-S3	none	MAFK	9.23E-20	1.88	0.91	2.01E-19	18.70	18.72
wgEncodeEH001898	NB4	none	CTCF	1.19E-19	2.02	1.02	2.58E-19	18.59	18.62
wgEncodeEH000703	K562	none	GTF2B	1.20E-19	2.10	1.07	2.61E-19	18.58	18.62
wgEncodeEH001656	T-47D	DMSO_0.02pct	CTCF	1.23E-19	1.95	0.96	2.67E-19	18.57	18.60
wgEncodeEH001491	A549	DEX_50nM	NR3C1	1.28E-19	1.86	0.90	2.78E-19	18.56	18.58
wgEncodeEH002822	A549	none	RAD21	1.31E-19	1.92	0.94	2.82E-19	18.55	18.57
GSM1505701	HUES64	none	NANOG	1.31E-19	1.87	0.90	2.82E-19	18.55	18.57

wgEncodeEH001207	K562	none	FOS	1.37E-19	1.87	0.90	2.95E-19	18.53	18.55
wgEncodeEH000396	HEK293	none	CTCF	1.51E-19	2.10	1.07	3.24E-19	18.49	18.52
wgEncodeEH000572	GM19240	none	CTCF	1.61E-19	2.12	1.09	3.45E-19	18.46	18.50
wgEncodeEH001812	GM12878	none	USF2	1.62E-19	1.88	0.91	3.46E-19	18.46	18.48
GSE76496.3	HEK293	none	MYNN	1.72E-19	1.86	0.90	3.67E-19	18.43	18.46
wgEncodeEH000559	NHEK	none	CTCF	1.90E-19	2.18	1.12	4.04E-19	18.39	18.43
wgEncodeEH001861	HepG2	none	SMC3	2.14E-19	1.94	0.96	4.54E-19	18.34	18.37
wgEncodeEH000543	HepG2	none	CTCF	2.46E-19	2.02	1.01	5.22E-19	18.28	18.31
wgEncodeEH000402	WERI-Rb-1	none	CTCF	2.72E-19	2.09	1.07	5.76E-19	18.24	18.27
GSE96210	HepG2	none	NR2F6	2.78E-19	1.88	0.91	5.88E-19	18.23	18.25
wgEncodeEH001593	H1-hESC	none	RAD21	2.97E-19	2.27	1.18	6.26E-19	18.20	18.25
GSE41629	SGBS	stimulation to differentiate	CEBPA	2.81E-19	1.85	0.89	5.93E-19	18.23	18.25
wgEncodeEH000569	GM12891	none	CTCF	3.02E-19	2.08	1.06	6.34E-19	18.20	18.23
GSE76496.6	HEK293	none	ZNF436	3.26E-19	1.86	0.89	6.84E-19	18.16	18.18
wgEncodeEH002806	GM12878	none	MAX	3.62E-19	1.87	0.90	7.58E-19	18.12	18.14
GSE90682	SK-N-SH	none	EBF3	4.03E-19	1.85	0.89	8.42E-19	18.07	18.09
wgEncodeEH001595	HepG2	none	HNF4A	4.05E-19	1.85	0.89	8.45E-19	18.07	18.09
wgEncodeEH002289	A549	EtOH_0.02pct	ELF1	4.47E-19	1.85	0.89	9.31E-19	18.03	18.05
wgEncodeEH001532	H1-hESC	none	USF1	4.74E-19	1.88	0.91	9.85E-19	18.01	18.03
wgEncodeEH001583	K562	none	USF1	4.79E-19	1.85	0.89	9.94E-19	18.00	18.02
GSE53041.3	GM10861	80 min calcitriol	VDR	5.25E-19	1.84	0.88	1.09E-18	17.96	17.98

GSE92059	MCF-7	none	RFX1	5.36E-19	1.86	0.90	1.11E-18	17.96	17.98
wgEncodeEH002843	H1-hESC	none	GTF2F1	6.24E-19	1.99	0.99	1.29E-18	17.89	17.92
wgEncodeEH000598	MCF-7	none	CTCF	7.83E-19	2.12	1.09	1.61E-18	17.79	17.83
wgEncodeEH001571	K562	none	BCLAF1	8.25E-19	1.94	0.95	1.70E-18	17.77	17.80
wgEncodeEH001576	K562	none	GATA2	8.27E-19	1.84	0.88	1.70E-18	17.77	17.79
wgEncodeEH001845	K562	none	SMC3	9.15E-19	1.89	0.92	1.88E-18	17.73	17.75
wgEncodeEH000712	GM12892	TNFa	NFKB1	9.51E-19	1.87	0.90	1.94E-18	17.71	17.73
wgEncodeEH000085	H1-hESC	none	CTCF	1.00E-18	2.19	1.13	2.04E-18	17.69	17.73
wgEncodeEH001651	HepG2	none	HNF4G	1.13E-18	1.84	0.88	2.31E-18	17.64	17.66
wgEncodeEH001846	GM12878	none	NRF1	1.23E-18	1.92	0.94	2.50E-18	17.60	17.63
wgEncodeEH000720	HeLa-S3	none	SMARCC1	1.25E-18	1.85	0.89	2.55E-18	17.59	17.61
wgEncodeEH002345	HepG2	none	TEAD4	1.37E-18	1.83	0.87	2.78E-18	17.56	17.58
GSE91422	HEK293	none	ZNF623	1.42E-18	1.83	0.87	2.88E-18	17.54	17.56
wgEncodeEH003434	MCF-7	serum_stimulated_media	CTCF	1.59E-18	2.14	1.10	3.21E-18	17.49	17.53
wgEncodeEH001836	H1-hESC	none	RAD21	2.00E-18	2.04	1.03	4.04E-18	17.39	17.42
wgEncodeEH001208	K562	none	GATA2	2.06E-18	1.85	0.89	4.14E-18	17.38	17.40
wgEncodeEH002805	K562	IFNa30	JUN	2.07E-18	1.83	0.87	4.15E-18	17.38	17.40
GSE106045	K562	none	NONO	2.22E-18	1.83	0.87	4.46E-18	17.35	17.37
GSM1693904	hTERT-RPE1	none	HINFP	2.42E-18	1.83	0.87	4.84E-18	17.31	17.33
wgEncodeEH000073	NHEK	none	POLR2A	2.54E-18	1.94	0.96	5.09E-18	17.29	17.32
wgEncodeEH001127	Fibroblasts	none	JUND	2.87E-18	2.07	1.05	5.72E-18	17.24	17.28

GSE77039.1	HT29	2h Rosiglitazone	PPARG	2.85E-18	1.83	0.87	5.69E-18	17.24	17.26
wgEncodeEH000734	GM10847	TNFa	NFKB1	3.08E-18	1.83	0.88	6.13E-18	17.21	17.23
GSM1692868	ATCC HTB-96	none	ARNTL	3.26E-18	1.82	0.86	6.47E-18	17.19	17.21
wgEncodeEH002383	K562	none	CBX3	4.20E-18	1.82	0.87	8.34E-18	17.08	17.10
wgEncodeEH001799	HeLa-S3	none	STAT3	4.89E-18	1.82	0.86	9.67E-18	17.01	17.03
GSE91518	K562	none	NR4A1	5.63E-18	1.81	0.86	1.11E-17	16.95	16.97
wgEncodeEH002269	SK-N-SH	none	REST	6.08E-18	1.82	0.86	1.20E-17	16.92	16.94
GSM1208791	LoVo	none	RORA	6.35E-18	1.98	0.98	1.25E-17	16.90	16.93
wgEncodeEH000647	HeLa-S3	none	FOS	6.52E-18	1.83	0.87	1.28E-17	16.89	16.91
wgEncodeEH001468	GM12878	none	USF1	8.21E-18	1.81	0.85	1.61E-17	16.79	16.81
wgEncodeEH002066	HeLa-S3	none	NFYA	8.92E-18	1.85	0.88	1.75E-17	16.76	16.78
GSM1505754	HUES64	none	SMAD4	9.20E-18	1.89	0.92	1.80E-17	16.75	16.77
GSE92196	K562	none	RFX1	9.17E-18	1.81	0.86	1.79E-17	16.75	16.77
GSE97661.1	GM12878	none	ETV6	1.14E-17	1.82	0.86	2.23E-17	16.65	16.67
wgEncodeEH001602	T-47D	DMSO_0.02pct	EP300	1.37E-17	1.82	0.86	2.67E-17	16.57	16.59
wgEncodeEH001486	GM12878	none	BCL11A	1.71E-17	1.80	0.85	3.31E-17	16.48	16.50
wgEncodeEH003210	K562	none	TRIM28	1.73E-17	1.80	0.85	3.35E-17	16.47	16.49
GSE91663	K562	none	ZEB2	1.75E-17	1.79	0.84	3.38E-17	16.47	16.49
wgEncodeEH002342	A549	EtOH_0.02pct	SIN3A	1.89E-17	1.86	0.89	3.65E-17	16.44	16.46
GSE94782.3	NB1643	none	MYCN	1.89E-17	1.81	0.85	3.65E-17	16.44	16.46
wgEncodeEH001585	K562	none	RAD21	1.97E-17	1.91	0.93	3.80E-17	16.42	16.44
wgEncodeEH001833	GM12878	none	SMC3	2.13E-17	1.83	0.87	4.10E-17	16.39	16.41

wgEncodeEH001574	H1-hESC	none	EP300	2.40E-17	1.81	0.85	4.60E-17	16.34	16.36
GSE96009	iPSC	none	POU5F1	2.70E-17	1.79	0.84	5.17E-17	16.29	16.31
wgEncodeEH002347	K562	none	STAT5A	3.21E-17	1.80	0.85	6.14E-17	16.21	16.23
wgEncodeEH001803	HepG2	none	RAD21	3.58E-17	1.91	0.93	6.84E-17	16.17	16.19
wgEncodeEH001506	HepG2	none	RXRA	3.97E-17	1.78	0.83	7.57E-17	16.12	16.14
GSM1407630	HEK293	none	ZNF189	4.35E-17	1.79	0.84	8.29E-17	16.08	16.10
wgEncodeEH001530	H1-hESC	none	SIN3A	4.71E-17	1.78	0.84	8.96E-17	16.05	16.07
wgEncodeEH002095	H1-hESC	none	CHD1	4.84E-17	1.85	0.88	9.19E-17	16.04	16.06
wgEncodeEH002315	GM12878	none	TCF3	5.23E-17	1.78	0.83	9.91E-17	16.00	16.02
wgEncodeEH001832	GM12878	none	EBF1	5.64E-17	1.78	0.83	1.07E-16	15.97	15.99
wgEncodeEH000562	GM12892	none	CTCF	5.95E-17	2.02	1.01	1.12E-16	15.95	15.98
GSE76496.22	HEK293	none	ZNF140	6.85E-17	1.78	0.83	1.29E-16	15.89	15.91
GSE91989	HEK293	none	ZNF146	8.02E-17	1.81	0.86	1.51E-16	15.82	15.84
wgEncodeEH001796	K562	none	NRF1	8.41E-17	1.86	0.90	1.58E-16	15.80	15.82
GSE105823	K562	none	MTA1	9.08E-17	1.78	0.83	1.70E-16	15.77	15.79
wgEncodeEH001568	HepG2	none	ATF3	9.28E-17	1.93	0.95	1.74E-16	15.76	15.79
wgEncodeEH003437	MCF-7	serum_starved_media	CTCF	1.01E-16	2.00	1.00	1.89E-16	15.72	15.75
wgEncodeEH001649	H1-hESC	none	CTCF	1.05E-16	1.98	0.99	1.96E-16	15.71	15.74
wgEncodeEH000659	K562	IFNa30	MYC	1.21E-16	1.78	0.83	2.25E-16	15.65	15.67
GSM1239545	LoVo	none	ZNF281	1.44E-16	1.79	0.84	2.68E-16	15.57	15.59
wgEncodeEH001648	GM12878	none	MEF2C	1.53E-16	1.80	0.85	2.84E-16	15.55	15.57

wgEncodeEH002032	HepG2	none	BHLHE40	1.65E-16	1.76	0.82	3.06E-16	15.51	15.53
GSE96327	K562	none	NR2F6	1.77E-16	1.76	0.82	3.27E-16	15.49	15.50
GSE76496.9	HEK293	none	ZSCAN31	1.78E-16	1.76	0.82	3.29E-16	15.48	15.50
wgEncodeEH000699	HeLa-S3	none	E2F1	2.15E-16	1.96	0.97	3.98E-16	15.40	15.43
GSE94577.2	VCaP-LTAD	DHT 10nM 24h	AR	2.41E-16	1.81	0.86	4.44E-16	15.35	15.37
wgEncodeEH002860	HepG2	none	MAZ	2.73E-16	1.80	0.84	5.02E-16	15.30	15.32
wgEncodeEH001654	K562	none	TAF7	2.94E-16	1.93	0.95	5.41E-16	15.27	15.30
wgEncodeEH001823	K562	none	GTF2F1	3.17E-16	1.91	0.93	5.82E-16	15.24	15.26
GSE58685	hESC	none	PDX1	3.14E-16	1.77	0.82	5.76E-16	15.24	15.26
wgEncodeEH002291	A549	EtOH_0.02pct	USF1	3.67E-16	1.77	0.82	6.70E-16	15.17	15.19
wgEncodeEH000727	K562	none	POLR2A	4.44E-16	1.76	0.81	8.11E-16	15.09	15.11
GSM1505702	HUES64	none	NANOG	4.91E-16	1.75	0.81	8.96E-16	15.05	15.07
wgEncodeEH001827	K562	none	MXI1	5.15E-16	1.77	0.82	9.37E-16	15.03	15.05
wgEncodeEH002028	HeLa-S3	none	ZNF143	8.06E-16	1.77	0.82	1.47E-15	14.83	14.85
GSM1208735	LoVo	none	ERG	8.96E-16	1.97	0.97	1.63E-15	14.79	14.82
wgEncodeEH002309	PFSK-1	none	TAF1	1.08E-15	1.97	0.98	1.95E-15	14.71	14.74
wgEncodeEH001563	GM12878	none	BCLAF1	1.05E-15	1.77	0.82	1.91E-15	14.72	14.74
GSM1407644	HEK293	none	ZNF322	1.18E-15	1.75	0.81	2.14E-15	14.67	14.69
GSM1239460	LoVo	none	HOXA4	1.24E-15	1.74	0.80	2.24E-15	14.65	14.67
GSM1239461	LoVo	none	HOXA5	1.24E-15	1.74	0.80	2.24E-15	14.65	14.67
wgEncodeEH001663	K562	none	MEF2A	1.33E-15	1.81	0.86	2.39E-15	14.62	14.64
wgEncodeEH000692	HeLa-S3	none	E2F6	1.83E-15	1.81	0.85	3.29E-15	14.48	14.51

wgEncodeEH000619	K562	none	FOS	1.92E-15	1.74	0.80	3.45E-15	14.46	14.48
wgEncodeEH000668	K562	IFNg6h	JUN	2.19E-15	1.76	0.82	3.92E-15	14.41	14.43
wgEncodeEH002288	A549	EtOH_0.02pct	ATF3	2.26E-15	1.78	0.84	4.04E-15	14.39	14.41
wgEncodeEH001462	GM12878	none	GABPA	3.03E-15	1.74	0.80	5.41E-15	14.27	14.29
wgEncodeEH000561	HUVEC	none	MYC	3.18E-15	1.78	0.83	5.66E-15	14.25	14.27
wgEncodeEH001772	MCF10A-Er-Src	EtOH_0.01pct	STAT3	3.29E-15	1.73	0.79	5.85E-15	14.23	14.25
GSM1239445	LoVo	none	NR5A2	3.61E-15	1.72	0.78	6.41E-15	14.19	14.21
GSE76496.19	HEK293	none	ZNF33A	3.78E-15	1.72	0.78	6.70E-15	14.17	14.19
GSE91996	HEK293	none	MYNN	4.13E-15	1.89	0.92	7.31E-15	14.14	14.16
wgEncodeEH002311	A549	EtOH_0.02pct	REST	4.42E-15	1.72	0.78	7.81E-15	14.11	14.13
wgEncodeEH002853	GM12878	none	TBL1XR1	6.43E-15	1.76	0.81	1.13E-14	13.95	13.97
wgEncodeEH001638	K562	none	REST	7.53E-15	1.70	0.77	1.33E-14	13.88	13.90
wgEncodeEH000723	HeLa-S3	none	NRF1	9.33E-15	1.92	0.94	1.64E-14	13.78	13.82
GSM1462461	CXCR5hi CD4 T cells	none	BCL6	1.08E-14	1.79	0.84	1.90E-14	13.72	13.74
GSE91713	MCF7	none	ELK1	1.12E-14	1.80	0.85	1.96E-14	13.71	13.73
GSE76496.8	HEK293	none	ZBTB18	1.11E-14	1.72	0.78	1.95E-14	13.71	13.73
wgEncodeEH000673	K562	IFNg30	JUN	1.20E-14	1.71	0.78	2.11E-14	13.68	13.69
GSE91907	HEK293	none	KLF7	1.22E-14	1.74	0.80	2.13E-14	13.67	13.69
wgEncodeEH002861	K562	none	ARID3A	1.44E-14	1.72	0.79	2.51E-14	13.60	13.62
wgEncodeEH000667	K562	IFNa6h	JUN	1.68E-14	1.76	0.81	2.93E-14	13.53	13.55
wgEncodeEH001622	K562	none	HDAC2	1.82E-14	1.73	0.79	3.16E-14	13.50	13.52
GSM2480806	LNCaP	none	ARID1A	1.94E-14	1.85	0.89	3.36E-14	13.47	13.50

wgEncodeEH003214	H1-hESC	none	TEAD4	1.97E-14	1.69	0.76	3.41E-14	13.47	13.49
GSE97661.2	GM12878	none	BATF	2.63E-14	1.69	0.75	4.56E-14	13.34	13.36
wgEncodeEH000649	K562	none	RAD21	2.82E-14	1.69	0.76	4.87E-14	13.31	13.33
wgEncodeEH000547	GM12878	none	MYC	3.08E-14	1.80	0.85	5.31E-14	13.27	13.30
wgEncodeEH001564	GM12878	none	ETS1	3.22E-14	1.77	0.83	5.54E-14	13.26	13.28
wgEncodeEH002022	HEK293	none	TCF7L2	3.29E-14	1.81	0.86	5.65E-14	13.25	13.27
GSE77039.2	HT29	48h Rosiglitazone	PPARG	3.91E-14	1.74	0.80	6.72E-14	13.17	13.19
wgEncodeEH002296	HepG2	none	ZBTB7A	4.98E-14	1.69	0.76	8.55E-14	13.07	13.09
GSE96119	K562	none	ZBTB11	5.34E-14	1.73	0.79	9.14E-14	13.04	13.06
wgEncodeEH000715	GM18505	TNFa	NFKB1	5.50E-14	1.70	0.77	9.41E-14	13.03	13.05
wgEncodeEH002827	H1-hESC	none	CHD2	6.59E-14	1.69	0.76	1.12E-13	12.95	12.97
wgEncodeEH002847	K562	none	RCOR1	6.67E-14	1.73	0.79	1.14E-13	12.94	12.96
wgEncodeEH000653	NT2-D1	none	YY1	8.29E-14	1.75	0.81	1.41E-13	12.85	12.87
GSM1537612	h9 hESC	none	NANOG	9.94E-14	1.67	0.74	1.69E-13	12.77	12.79
GSE92092	K562	none	HDAC8	1.28E-13	1.69	0.76	2.17E-13	12.66	12.68
GSE105998	GM12878	none	ZNF217	1.40E-13	1.67	0.74	2.37E-13	12.63	12.64
wgEncodeEH001652	K562	none	CTCF	1.42E-13	1.66	0.73	2.40E-13	12.62	12.64
wgEncodeEH001672	HCT-116	none	ZBTB33	1.52E-13	1.73	0.79	2.57E-13	12.59	12.61
wgEncodeEH002863	K562	none	UBTF	1.56E-13	1.81	0.86	2.63E-13	12.58	12.61
GSM1056832	macrophage	4h 1% O2	HIF1A	1.56E-13	1.72	0.78	2.64E-13	12.58	12.60
wgEncodeEH002300	ECC-1	DMSO_0.02pct	CTCF	1.95E-13	1.68	0.74	3.29E-13	12.48	12.50

wgEncodeEH000683	K562	none	GATA2	2.16E-13	1.67	0.74	3.63E-13	12.44	12.46
wgEncodeEH002072	MCF-7	none	TCF7L2	2.27E-13	1.70	0.77	3.81E-13	12.42	12.44
GSE97661.3	HepG2	none	ARID3A	2.62E-13	1.71	0.77	4.39E-13	12.36	12.38
GSE97661.9	GM12878	none	STAT2	2.74E-13	1.86	0.89	4.58E-13	12.34	12.37
GSE97661.4	HepG2	none	FOXO3	3.25E-13	1.87	0.90	5.44E-13	12.26	12.30
wgEncodeEH002266	PANC-1	none	SIN3A	3.44E-13	1.84	0.88	5.74E-13	12.24	12.27
wgEncodeEH001837	H1-hESC	none	USF2	3.40E-13	1.69	0.76	5.67E-13	12.25	12.27
wgEncodeEH002842	H1-hESC	none	BACH1	3.69E-13	1.65	0.72	6.14E-13	12.21	12.23
wgEncodeEH000620	K562	none	JUN	3.82E-13	1.65	0.72	6.34E-13	12.20	12.22
wgEncodeEH002295	A549	EtOH_0.02pct	ZBTB33	4.05E-13	1.68	0.75	6.71E-13	12.17	12.19
GSM1208762	LoVo	none	JDP2	4.49E-13	2.03	1.02	7.43E-13	12.13	12.17
wgEncodeEH002824	GM12878	none	EP300	4.71E-13	1.67	0.74	7.79E-13	12.11	12.13
GSE112326.1	aortic smooth muscle cells	none	SMAD2	5.46E-13	1.87	0.91	9.01E-13	12.05	12.08
wgEncodeEH001847	H1-hESC	none	NRF1	7.89E-13	1.72	0.78	1.30E-12	11.89	11.91
GSE91427	K562	none	TARDBP	8.07E-13	1.66	0.73	1.33E-12	11.88	11.90
GSM1208743	LoVo	none	ZFHX3	9.07E-13	1.71	0.78	1.49E-12	11.83	11.85
wgEncodeEH001658	GM12878	none	BCL3	1.00E-12	1.64	0.71	1.64E-12	11.78	11.80
wgEncodeEH002307	GM12878	none	NFATC1	1.01E-12	1.65	0.72	1.66E-12	11.78	11.80
wgEncodeEH002829	H1-hESC	none	MXI1	1.04E-12	1.66	0.73	1.70E-12	11.77	11.79
wgEncodeEH001637	K562	none	FOSL1	1.08E-12	1.63	0.71	1.76E-12	11.75	11.77
wgEncodeEH002303	A549	EtOH_0.02pct	BCL3	1.27E-12	1.67	0.74	2.08E-12	11.68	11.70
GSM1056836	macrophage	4h 1% O2	EPAS1	1.52E-12	1.75	0.81	2.48E-12	11.61	11.63

wgEncodeEH003212	GM12878	none	CEBPB	1.52E-12	1.71	0.77	2.48E-12	11.61	11.63
GSE76496.12	HEK293	none	ZFP28	1.62E-12	1.76	0.81	2.63E-12	11.58	11.61
GSE76496.25	HEK293	none	ZNF250	1.80E-12	1.77	0.82	2.92E-12	11.54	11.56
wgEncodeEH001477	GM12878	none	PBX3	1.91E-12	1.63	0.70	3.10E-12	11.51	11.53
wgEncodeEH002825	H1-hESC	none	CEBPB	1.94E-12	1.63	0.70	3.14E-12	11.50	11.52
GSM1867061	SK-N-BE(2)-C	none	MYCN	2.15E-12	1.64	0.72	3.47E-12	11.46	11.48
GSM2257668	U2OS	none	ARNTL	2.34E-12	1.64	0.71	3.78E-12	11.42	11.44
GSM1208631	LoVo	none	HINFP	2.42E-12	1.66	0.73	3.91E-12	11.41	11.43
wgEncodeEH003356	K562	none	ELK1	2.60E-12	1.78	0.83	4.18E-12	11.38	11.41
GSE94577.1	VCaP-LTAD	DHT 1nM 24h	AR	2.71E-12	1.68	0.75	4.35E-12	11.36	11.38
GSE91561	MCF7	none	SREBF1	3.30E-12	1.71	0.78	5.29E-12	11.28	11.30
GSE76496.18	HEK293	none	ZNF146	3.78E-12	1.62	0.70	6.06E-12	11.22	11.23
wgEncodeEH001538	H1-hESC	none	EGR1	3.90E-12	1.62	0.69	6.25E-12	11.20	11.22
GSM1438986	ICN12	none	BCL6	3.98E-12	1.68	0.75	6.36E-12	11.20	11.22
wgEncodeEH001566	H1-hESC	none	ATF3	4.22E-12	1.67	0.74	6.72E-12	11.17	11.19
GSE76496.14	HEK293	none	ZNF322	4.20E-12	1.61	0.69	6.71E-12	11.17	11.19
GSM2257669	U2OS	none	ARNTL	4.49E-12	1.62	0.70	7.15E-12	11.15	11.16
wgEncodeEH002849	K562	none	TBL1XR1	7.31E-12	1.70	0.76	1.16E-11	10.93	10.96
wgEncodeEH001541	GM12878	none	RXRA	7.95E-12	1.93	0.95	1.26E-11	10.90	10.94
GSM1505762	HUES64	none	SOX17	9.43E-12	1.67	0.74	1.49E-11	10.83	10.85
wgEncodeEH000397	HL-60	none	CTCF	1.01E-11	1.60	0.67	1.59E-11	10.80	10.81
wgEncodeEH000542	HeLa-S3	none	MYC	1.05E-11	1.77	0.82	1.67E-11	10.78	10.81
wgEncodeEH001611	HepG2	none	SRF	1.07E-11	1.68	0.75	1.69E-11	10.77	10.79

wgEncodeEH002316	H1-hESC	none	ATF2	1.44E-11	1.64	0.72	2.28E-11	10.64	10.66
GSM1208693	LoVo	none	TFE3	1.54E-11	1.73	0.79	2.43E-11	10.61	10.64
wgEncodeEH000717	GM19099	TNFa	NFKB1	1.80E-11	1.63	0.71	2.83E-11	10.55	10.57
GSE91535	K562	none	ARNT	2.06E-11	1.62	0.70	3.23E-11	10.49	10.51
GSE96003	HepG2	none	ATF1	2.53E-11	1.66	0.73	3.96E-11	10.40	10.42
wgEncodeEH001021	HeLa-S3	none	POLR2A	2.70E-11	1.77	0.82	4.22E-11	10.38	10.40
GSE91894	A549	none	NFE2L2	2.67E-11	1.68	0.75	4.18E-11	10.38	10.40
wgEncodeEH002321	GM12878	none	STAT5A	3.81E-11	1.61	0.69	5.94E-11	10.23	10.24
wgEncodeEH000621	K562	none	MYC	3.83E-11	1.62	0.70	5.97E-11	10.22	10.24
GSM1239498	LoVo	none	E2F8	5.32E-11	1.92	0.94	8.26E-11	10.08	10.13
wgEncodeEH002031	SH-SY5Y	none	GATA3	5.26E-11	1.68	0.75	8.19E-11	10.09	10.11
wgEncodeEH000753	HepG2	forskolin	HNF4A	5.27E-11	1.58	0.66	8.20E-11	10.09	10.10
GSM1495181	U2OS	10 min heat shock	HSF1	5.59E-11	1.57	0.65	8.67E-11	10.06	10.08
GSM1208644	LoVo	none	LHX2	5.85E-11	1.60	0.68	9.06E-11	10.04	10.06
GSE112326.2	aortic smooth muscle cells	TGFb 10 days	SMAD2	7.20E-11	1.66	0.74	1.11E-10	9.95	9.98
wgEncodeEH000639	GM12878	none	JUND	7.57E-11	1.90	0.93	1.17E-10	9.93	9.97
wgEncodeEH002812	MCF-7	none	GATA3	8.23E-11	1.61	0.69	1.27E-10	9.90	9.91
GSM1208720	LoVo	none	CREB3	9.06E-11	1.83	0.87	1.40E-10	9.86	9.89
GSE91643	K562	none	NCOR1	9.83E-11	1.63	0.70	1.51E-10	9.82	9.84
wgEncodeEH000560	H1-hESC	none	CTCF	1.23E-10	1.63	0.71	1.89E-10	9.72	9.74
GSE53041.1	GM10855	80 min calcitriol	VDR	1.29E-10	1.64	0.71	1.97E-10	9.70	9.73
GSE92148	A549	none	SREBF1	1.46E-10	1.76	0.81	2.23E-10	9.65	9.68

wgEncodeEH000759	HepG2	insulin	SREBF1	1.58E-10	1.75	0.81	2.43E-10	9.62	9.64
wgEncodeEH002851	GM12878	none	ELK1	1.69E-10	1.82	0.87	2.59E-10	9.59	9.62
wgEncodeEH001536	ECC-1	Estradiol_10nM	ESR1	1.94E-10	1.57	0.65	2.96E-10	9.53	9.55
wgEncodeEH001840	HepG2	none	CHD2	2.01E-10	1.72	0.78	3.07E-10	9.51	9.54
wgEncodeEH001810	GM12878	none	RFX5	2.29E-10	1.84	0.88	3.49E-10	9.46	9.49
wgEncodeEH002846	K562	none	BACH1	2.27E-10	1.69	0.76	3.46E-10	9.46	9.49
GSM1495182	U2OS	20 min heat shock	HSF1	2.39E-10	1.55	0.64	3.64E-10	9.44	9.46
GSE92187	MCF7	none	ESRRA	2.56E-10	1.56	0.64	3.88E-10	9.41	9.43
GSM1239506	LoVo	none	ID1	2.67E-10	1.71	0.77	4.04E-10	9.39	9.42
GSM1208776	LoVo	none	NFKB2	2.68E-10	1.68	0.75	4.06E-10	9.39	9.42
wgEncodeEH002290	A549	EtOH_0.02pct	ETS1	3.07E-10	1.59	0.67	4.64E-10	9.33	9.35
GSM1122314	ME-1	none	ERG	3.21E-10	1.55	0.64	4.84E-10	9.32	9.33
wgEncodeEH002314	GM12878	none	REST	3.38E-10	1.60	0.68	5.09E-10	9.29	9.31
wgEncodeEH001134	MCF-7	estrogen	MYC	3.48E-10	1.58	0.66	5.22E-10	9.28	9.30
wgEncodeEH001757	H1-hESC	none	MAX	3.47E-10	1.54	0.62	5.21E-10	9.28	9.30
GSM2466489	HEK293T	none	ZNF189	3.93E-10	1.67	0.74	5.89E-10	9.23	9.25
wgEncodeEH001575	ECC-1	Genistein_100nM	ESR1	4.20E-10	1.58	0.66	6.30E-10	9.20	9.22
GSE43111	EP156T	none	TP63	4.63E-10	1.66	0.73	6.92E-10	9.16	9.18
GSM2466520	HEK293T	none	ZNF287	1.12E-09	3.43	1.78	1.64E-09	8.79	9.12
GSM2131193	T47D	none	KDM5A	5.65E-10	1.67	0.74	8.44E-10	9.07	9.10
wgEncodeEH001600	K562	none	SRF	5.62E-10	1.60	0.68	8.40E-10	9.08	9.10
wgEncodeEH002024	K562	none	NFYB	5.70E-10	1.56	0.64	8.50E-10	9.07	9.09

wgEncodeEH001533	H1-hESC	none	SRF	6.63E-10	1.63	0.70	9.87E-10	9.01	9.03
wgEncodeEH001483	K562	none	SIX5	7.69E-10	1.62	0.69	1.14E-09	8.94	8.96
wgEncodeEH002795	H1-hESC	none	MYC	7.94E-10	1.59	0.67	1.18E-09	8.93	8.95
wgEncodeEH000716	GM18526	TNFa	NFKB1	8.35E-10	1.70	0.77	1.24E-09	8.91	8.94
wgEncodeEH002023	H1-hESC	none	JUND	8.74E-10	1.53	0.61	1.29E-09	8.89	8.90
GSM1703893	DOHH2	none	BACH2	9.27E-10	1.63	0.70	1.37E-09	8.86	8.88
wgEncodeEH001629	HeLa-S3	none	REST	9.75E-10	1.56	0.64	1.44E-09	8.84	8.86
wgEncodeEH002868	GM12878	none	SIN3A	1.01E-09	1.62	0.70	1.49E-09	8.83	8.85
wgEncodeEH001653	K562	none	SP2	1.04E-09	1.67	0.74	1.53E-09	8.82	8.84
GSE76496.2	HEK293	none	ZNF224	1.06E-09	1.62	0.69	1.56E-09	8.81	8.83
GSE53041.2	GM10855	none	VDR	1.11E-09	1.68	0.75	1.63E-09	8.79	8.81
GSM970261	ProEs	none	IRF2	1.36E-09	1.59	0.67	1.99E-09	8.70	8.72
wgEncodeEH001556	T-47D	Genistein_100nM	ESR1	1.47E-09	1.61	0.68	2.15E-09	8.67	8.69
wgEncodeEH001577	T-47D	Estradiol_10nM	ESR1	1.47E-09	1.61	0.68	2.15E-09	8.67	8.69
GSM558470	HeLa	none	PHF8	1.48E-09	1.57	0.65	2.16E-09	8.67	8.68
GSM2480810	LNCaP	none	SMARCA4	1.59E-09	1.73	0.79	2.31E-09	8.64	8.67
wgEncodeEH000665	K562	IFNa30	STAT2	1.98E-09	1.64	0.72	2.89E-09	8.54	8.56
GSE79496.20	HEK293	none	ZNF121	2.05E-09	1.62	0.70	2.98E-09	8.53	8.55
wgEncodeEH001843	HepG2	none	RFX5	2.13E-09	1.73	0.79	3.10E-09	8.51	8.54
wgEncodeEH002317	H1-hESC	none	SP4	2.55E-09	1.54	0.62	3.70E-09	8.43	8.45
GSE91618	HepG2	none	FOXA1	2.69E-09	1.62	0.70	3.90E-09	8.41	8.43
wgEncodeEH001635	H1-hESC	none	NANOG	2.85E-09	1.66	0.73	4.13E-09	8.38	8.41

wgEncodeEH001767	H1-hESC	none	CTBP2	3.02E-09	1.55	0.64	4.36E-09	8.36	8.38
wgEncodeEH002341	A549	EtOH_0.02pct	SIX5	3.27E-09	1.56	0.64	4.72E-09	8.33	8.35
GSM1290023	T3M-1 Cl-10	none	ELF4	3.66E-09	1.55	0.63	5.28E-09	8.28	8.30
GSM1208792	LoVo	none	SATB1	3.90E-09	1.79	0.84	5.62E-09	8.25	8.29
wgEncodeEH000624	K562	none	NFE2	3.94E-09	1.68	0.75	5.67E-09	8.25	8.27
GSM1438987	ICN12	none	TCF3	3.98E-09	1.51	0.60	5.72E-09	8.24	8.26
wgEncodeEH001542	GM12878	none	SIX5	4.08E-09	1.56	0.64	5.85E-09	8.23	8.25
wgEncodeEH001659	H1-hESC	none	HDAC2	4.70E-09	1.57	0.65	6.74E-09	8.17	8.19
GSE37589	GM11992	none	NFE2L2	5.41E-09	1.49	0.58	7.75E-09	8.11	8.13
GSE95952	MCF7	none	ZBTB11	6.80E-09	1.65	0.73	9.73E-09	8.01	8.04
GSE92043	K562	TNF	RELA	6.95E-09	1.61	0.69	9.92E-09	8.00	8.03
wgEncodeEH002833	K562	none	POLR2A	7.74E-09	1.57	0.65	1.10E-08	7.96	7.98
GSE76496.13	HEK293	none	ZFP82	8.48E-09	1.64	0.72	1.21E-08	7.92	7.94
GSM1208590	LoVo	none	ARNT	1.02E-08	1.55	0.63	1.46E-08	7.84	7.86
wgEncodeEH002069	HeLa-S3	none	TCF7L2	1.14E-08	1.67	0.74	1.62E-08	7.79	7.82
wgEncodeEH002096	H1-hESC	none	KDM5A	1.27E-08	1.81	0.85	1.80E-08	7.74	7.79
GSM1703916	OCI-LY-7	none	BACH2	1.44E-08	1.63	0.70	2.05E-08	7.69	7.71
wgEncodeEH002037	GM12878	none	EP300	1.48E-08	1.57	0.65	2.09E-08	7.68	7.70
GSE105635	K562	none	NFATC3	1.54E-08	1.62	0.70	2.17E-08	7.66	7.69
wgEncodeEH001492	A549	DEX_5nM	NR3C1	1.69E-08	1.56	0.64	2.39E-08	7.62	7.64
GSM1505679	HUES64	none	HEY1	1.82E-08	1.83	0.87	2.56E-08	7.59	7.64
GSM2466476	HEK293T	none	ZNF133	1.98E-08	1.84	0.88	2.78E-08	7.56	7.60
GSM1537615	NT2-D1	none	NANOG	1.88E-08	1.48	0.57	2.65E-08	7.58	7.59
GSM830122	keratinocytes	3 days Ca2+	TP63	1.90E-08	1.55	0.63	2.68E-08	7.57	7.59

wgEncodeEH002036	MCF-7	none	ZNF217	2.01E-08	1.55	0.63	2.82E-08	7.55	7.57
GSE97661.7	MCF7	none	KLF10	2.05E-08	1.55	0.63	2.88E-08	7.54	7.56
GSE96382	K562	none	JUNB	2.25E-08	1.59	0.67	3.15E-08	7.50	7.52
GSE76496.11	HEK293	none	ZFP28	2.43E-08	1.56	0.64	3.40E-08	7.47	7.49
GSE76496.5	HEK293	none	ZNF350	2.45E-08	1.56	0.64	3.42E-08	7.47	7.49
GSM1816446	NOMO-1	none	ARNTL	2.74E-08	1.58	0.66	3.82E-08	7.42	7.44
wgEncodeEH001540	ECC-1	DEX_100nM	NR3C1	2.97E-08	1.52	0.60	4.14E-08	7.38	7.40
wgEncodeEH001569	K562	none	ZBTB33	3.20E-08	1.61	0.68	4.45E-08	7.35	7.38
GSE88734.2	RKO	none	ZEB1	3.38E-08	1.49	0.58	4.69E-08	7.33	7.34
wgEncodeEH000718	GM19193	TNFa	NFKB1	3.58E-08	1.54	0.63	4.98E-08	7.30	7.32
wgEncodeEH002828	H1-hESC	none	MAFK	4.36E-08	1.50	0.58	6.05E-08	7.22	7.24
GSE99889	CD4+T	none	HIC1	4.47E-08	1.53	0.61	6.20E-08	7.21	7.23
wgEncodeEH000724	K562	none	SMARCA4	4.94E-08	1.66	0.73	6.83E-08	7.17	7.20
GSM1407634	HEK293	none	ZNF35	5.59E-08	1.61	0.69	7.73E-08	7.11	7.14
GSM558472	HeLa	none	PHF8	6.30E-08	1.48	0.57	8.69E-08	7.06	7.08
GSM830121	keratinocytes	none	TP63	6.62E-08	1.57	0.65	9.13E-08	7.04	7.06
wgEncodeEH000781	HeLa-S3	none	SMARCA4	7.45E-08	1.79	0.84	1.03E-07	6.99	7.03
GSE105407	MCF7	none	KLF4	8.59E-08	1.50	0.59	1.18E-07	6.93	6.95
wgEncodeEH000721	HeLa-S3	none	SMARCC2	8.98E-08	1.67	0.74	1.23E-07	6.91	6.94
wgEncodeEH002302	H1-hESC	none	SP2	9.06E-08	1.63	0.70	1.24E-07	6.91	6.93
GSM1505755	HUES64	none	SMAD4	1.32E-07	1.63	0.70	1.80E-07	6.74	6.77
wgEncodeEH000689	HeLa-S3	none	E2F4	1.45E-07	1.67	0.74	1.99E-07	6.70	6.74
GSM558471	HeLa	none	PHF8	1.52E-07	1.58	0.66	2.07E-07	6.68	6.71
wgEncodeEH001655	K562	none	THAP1	1.53E-07	1.54	0.62	2.09E-07	6.68	6.70

wgEncodeEH002864	HeLa-S3	none	ELK1	1.57E-07	1.61	0.68	2.15E-07	6.67	6.70
wgEncodeEH001209	K562	none	HDAC8	1.69E-07	1.70	0.77	2.31E-07	6.64	6.67
wgEncodeEH001804	HepG2	none	USF2	1.82E-07	1.56	0.64	2.48E-07	6.61	6.63
GSM1505753	HUES64	none	SMAD4	1.95E-07	1.62	0.69	2.65E-07	6.58	6.61
wgEncodeEH002801	H1-hESC	none	BRCA1	2.00E-07	1.63	0.71	2.71E-07	6.57	6.60
GSE56674.1	keratyinocytes	none	TP53	2.53E-07	1.72	0.78	3.43E-07	6.47	6.51
GSM1505761	HUES64	none	SOX17	2.43E-07	1.48	0.56	3.30E-07	6.48	6.50
wgEncodeEH001817	HeLa-S3	none	PRDM1	2.59E-07	1.61	0.68	3.50E-07	6.46	6.48
GSE76376	HEE	none	HNF1A	2.84E-07	1.85	0.89	3.84E-07	6.42	6.47
GSM1693910	U2OS	none	HINFP	2.83E-07	1.46	0.55	3.83E-07	6.42	6.43
wgEncodeEH001545	GM12891	none	PAX5	3.61E-07	1.55	0.64	4.86E-07	6.31	6.34
wgEncodeEH000666	K562	IFNa6h	STAT2	4.60E-07	1.66	0.73	6.18E-07	6.21	6.24
wgEncodeEH001586	ECC-1	DMSO_0.02pct	FOXA1	4.47E-07	1.49	0.58	6.01E-07	6.22	6.24
wgEncodeEH000675	HepG2	none	NR2C2	4.58E-07	1.54	0.62	6.15E-07	6.21	6.23
GSM1505703	HUES64	none	NANOG	5.07E-07	1.68	0.75	6.80E-07	6.17	6.21
wgEncodeEH003439	MCF-7	serum_starved_media	MYC	5.68E-07	1.49	0.57	7.61E-07	6.12	6.14
wgEncodeEH001487	GM12878	none	EP300	6.05E-07	1.48	0.57	8.09E-07	6.09	6.11
GSM1505763	HUES64	none	SOX17	6.05E-07	1.44	0.53	8.09E-07	6.09	6.11
wgEncodeEH001854	H1-hESC	none	JUN	6.83E-07	1.79	0.84	9.12E-07	6.04	6.09
GSE91909	HepG2	none	TBX3	8.25E-07	1.54	0.62	1.10E-06	5.96	5.98
GSM1842789	KH2 mESC	none	POU5F1	8.74E-07	1.57	0.65	1.16E-06	5.93	5.96
GSE76496.28	HEK293	none	KLF7	1.03E-06	1.43	0.52	1.37E-06	5.86	5.88
wgEncodeEH002841	GM12878	none	RCOR1	1.11E-06	1.69	0.75	1.47E-06	5.83	5.87

wgEncodeEH002091	K562	none	HDAC2	1.07E-06	1.46	0.55	1.42E-06	5.85	5.87
GSE53041.4	GM10861	none	VDR	1.25E-06	1.47	0.55	1.66E-06	5.78	5.80
GSE105428	HEK293	none	ZNF133	1.31E-06	1.56	0.64	1.74E-06	5.76	5.79
GSE88734.1	MiaPaCa2	none	ZEB1	1.35E-06	1.46	0.55	1.79E-06	5.75	5.77
wgEncodeEH001498	H1-hESC	none	REST	1.47E-06	1.42	0.50	1.94E-06	5.71	5.73
wgEncodeEH001488	GM12878	none	ZBTB33	1.66E-06	1.58	0.66	2.19E-06	5.66	5.69
wgEncodeEH002821	A549	none	POLR2A	1.81E-06	1.64	0.71	2.39E-06	5.62	5.66
wgEncodeEH002817	A549	none	BHLHE40	1.77E-06	1.49	0.57	2.33E-06	5.63	5.65
GSE79694	ES	none	FOXM1	1.97E-06	1.82	0.86	2.59E-06	5.59	5.65
wgEncodeEH002033	K562	none	RFX5	1.89E-06	1.62	0.69	2.49E-06	5.60	5.64
wgEncodeEH001797	K562	none	USF2	2.23E-06	1.51	0.60	2.92E-06	5.53	5.56
GSM1208594	LoVo	none	CAMTA2	2.31E-06	1.42	0.51	3.04E-06	5.52	5.53
wgEncodeEH002826	H1-hESC	none	CHD1	2.48E-06	1.66	0.73	3.25E-06	5.49	5.53
wgEncodeEH001787	GM12878	none	WRNIP1	2.57E-06	1.63	0.71	3.37E-06	5.47	5.51
GSM1239489	LoVo	none	ARNTL	2.69E-06	1.47	0.55	3.52E-06	5.45	5.47
GSM1208775	LoVo	none	NFATC4	2.93E-06	1.73	0.79	3.83E-06	5.42	5.47
wgEncodeEH000677	K562	none	SETDB1	3.03E-06	1.54	0.62	3.96E-06	5.40	5.43
wgEncodeEH002021	K562	none	NFYA	3.84E-06	1.45	0.54	5.00E-06	5.30	5.32
wgEncodeEH001805	K562	none	POLR2A	4.13E-06	1.70	0.77	5.35E-06	5.27	5.32
GSM1275921	U937	none	MIXL1	3.88E-06	1.37	0.46	5.05E-06	5.30	5.31
GSM1208721	LoVo	none	CREB3L4	4.09E-06	1.57	0.65	5.31E-06	5.27	5.31
wgEncodeEH000664	K562	IFNa6h	STAT1	4.26E-06	1.63	0.70	5.53E-06	5.26	5.30
GSM1239468	LoVo	none	KLF11	4.97E-06	1.97	0.98	6.41E-06	5.19	5.28

wgEncodeEH000681	K562	none	SIRT6	4.43E-06	1.53	0.61	5.73E-06	5.24	5.27
GSE105731	K562	none	ARID2	4.37E-06	1.47	0.56	5.66E-06	5.25	5.27
GSE91919	K562	none	HINFP	4.48E-06	1.51	0.60	5.80E-06	5.24	5.26
GSM1122312	ME-1	none	FLI1	4.87E-06	1.39	0.48	6.28E-06	5.20	5.22
GSM1407627	HEK293	none	ZNF250	5.29E-06	1.63	0.70	6.81E-06	5.17	5.20
GSM1208772	LoVo	none	MSX1	6.30E-06	1.97	0.98	8.10E-06	5.09	5.18
GSE53041.5	LS180	80 min calcitriol	VDR	6.64E-06	1.49	0.58	8.53E-06	5.07	5.09
GSM1239665	MDA-MB-231	none	XBP1	3.61E-05	3.43	1.78	4.57E-05	4.34	4.98
GSM1239547	LoVo	none	ZNF639	1.06E-05	1.82	0.87	1.36E-05	4.87	4.94
wgEncodeEH002823	GM12878	none	CHD1	1.17E-05	1.75	0.80	1.49E-05	4.83	4.88
wgEncodeEH000700	K562	none	ATF3	1.16E-05	1.67	0.74	1.49E-05	4.83	4.87
GSE53041.7	LX2	80 min calcitriol	VDR	1.16E-05	1.65	0.72	1.49E-05	4.83	4.87
wgEncodeEH001534	H1-hESC	none	GABPA	1.09E-05	1.38	0.47	1.40E-05	4.85	4.87
wgEncodeEH001859	HepG2	none	BRCA1	1.58E-05	2.09	1.06	2.02E-05	4.70	4.82
GSM1208722	LoVo	none	CREM	1.63E-05	1.59	0.67	2.08E-05	4.68	4.72
wgEncodeEH000754	HepG2	forskolin	HSF1	1.74E-05	1.61	0.68	2.21E-05	4.65	4.69
wgEncodeEH001645	GM12878	none	ZEB1	1.84E-05	1.39	0.47	2.34E-05	4.63	4.65
wgEncodeEH000761	K562	IFNg6h	STAT1	1.90E-05	1.49	0.57	2.42E-05	4.62	4.64
GSE106048	HepG2	none	SMARCC2	3.01E-05	2.02	1.01	3.81E-05	4.42	4.53
GSE76496.23	HEK293	none	ZNF41	2.64E-05	1.40	0.48	3.35E-05	4.47	4.49
GSM2480808	LNCaP	none	SMARCA4	3.64E-05	1.80	0.85	4.60E-05	4.34	4.41
wgEncodeEH001503	HepG2	none	ZBTB33	3.37E-05	1.51	0.59	4.26E-05	4.37	4.40
wgEncodeEH001570	K562	none	BCL3	3.67E-05	1.54	0.62	4.63E-05	4.33	4.37

wgEncodeEH001549	HepG2	none	REST	3.65E-05	1.39	0.48	4.61E-05	4.34	4.35
GSM1056831	macrophage	4h 1% O2	HIF1A	3.98E-05	1.63	0.71	5.01E-05	4.30	4.35
GSM991660	IMR90	none	E2F7	3.94E-05	1.41	0.50	4.96E-05	4.30	4.32
GSM2390643	HUVEC	Hypoxia	ARNT	4.37E-05	1.57	0.65	5.50E-05	4.26	4.30
GSM2480804	LNCaP	none	ARID1A	4.82E-05	1.69	0.76	6.05E-05	4.22	4.27
wgEncodeEH002064	GM12878	none	NFYA	4.59E-05	1.55	0.63	5.76E-05	4.24	4.27
wgEncodeEH001830	GM12878	none	BRCA1	6.04E-05	1.85	0.89	7.56E-05	4.12	4.21
wgEncodeEH001531	H1-hESC	none	TCF12	5.49E-05	1.35	0.43	6.88E-05	4.16	4.18
wgEncodeEH002293	A549	DEX_100nM	FOXA1	7.29E-05	1.95	0.96	9.08E-05	4.04	4.15
wgEncodeEH000760	K562	IFNg30	STAT1	6.30E-05	1.50	0.59	7.88E-05	4.10	4.13
wgEncodeEH000630	K562	none	ZNF263	6.31E-05	1.45	0.53	7.88E-05	4.10	4.13
GSE53041.8	LX2	none	VDR	7.14E-05	1.59	0.67	8.90E-05	4.05	4.09
wgEncodeEH000765	HeLa-S3	none	BRF2	3.36E-02	4.79	2.26	3.70E-02	1.43	4.05
wgEncodeEH001553	PFSK-1	none	REST	7.52E-05	1.38	0.46	9.36E-05	4.03	4.05
wgEncodeEH001762	U2OS	none	SETDB1	8.58E-05	1.35	0.44	1.07E-04	3.97	3.99
GSM1239595	LoVo	none	MYBL1	9.80E-05	1.57	0.65	1.22E-04	3.91	3.96
wgEncodeEH001855	HeLa-S3	none	SUPT20H	8.14E-02	4.79	2.26	8.77E-02	1.06	3.93
wgEncodeEH000638	K562	none	GATA1	1.04E-04	1.37	0.45	1.29E-04	3.89	3.91
wgEncodeEH001865	K562	IFNa30	IRF1	1.25E-04	1.84	0.88	1.54E-04	3.81	3.91
GSE91509	K562	none	CDC5L	1.07E-04	1.41	0.50	1.33E-04	3.88	3.90
GSM1526875	HMEC-TERT	none	MYBL2	1.08E-04	1.39	0.48	1.34E-04	3.87	3.89
GSM2466612	HEK293T	none	ZNF613	1.30E-04	1.81	0.86	1.59E-04	3.80	3.88
GSM1208637	LoVo	none	HSF2	1.14E-04	1.48	0.57	1.41E-04	3.85	3.88

GSM856790	786-O	none	EPAS1	1.29E-04	1.70	0.77	1.58E-04	3.80	3.87
wgEncodeEH001515	HepG2	none	BHLHE40	1.18E-04	1.42	0.50	1.46E-04	3.83	3.86
wgEncodeEH000687	HeLa-S3	none	NR2C2	1.27E-04	1.57	0.65	1.57E-04	3.80	3.85
wgEncodeEH001636	H1-hESC	none	POU5F1	1.28E-04	1.47	0.56	1.58E-04	3.80	3.83
wgEncodeEH001528	H1-hESC	none	SIX5	1.30E-04	1.40	0.49	1.59E-04	3.80	3.82
GSM1122317	ME-1	none	EP300	1.34E-04	1.32	0.40	1.65E-04	3.78	3.80
GSE97661.5	K562	none	FOXO3	1.69E-04	1.43	0.52	2.06E-04	3.69	3.71
wgEncodeEH001544	HepG2	none	TCF12	1.72E-04	1.48	0.57	2.10E-04	3.68	3.71
GSM1239529	LoVo	none	TCF7L1	2.05E-04	1.53	0.61	2.51E-04	3.60	3.64
wgEncodeEH002035	MCF-7	none	GATA3	2.08E-04	1.54	0.63	2.54E-04	3.60	3.64
wgEncodeEH002857	HeLa-S3	none	ZKSCAN1	2.01E-04	1.40	0.49	2.46E-04	3.61	3.63
GSE91727	MCF-7	none	MTA3	2.76E-04	1.55	0.63	3.36E-04	3.47	3.52
GSE96148	K562	none	DEAF1	2.97E-04	1.40	0.49	3.61E-04	3.44	3.47
GSE92078	K562	none	FOXA1	3.79E-04	1.90	0.93	4.60E-04	3.34	3.46
GSM1208671	LoVo	none	RFX2	3.46E-04	1.31	0.39	4.21E-04	3.38	3.39
GSM1505694	HUES64	none	NR5A2	3.68E-04	1.36	0.45	4.47E-04	3.35	3.37
GSE95956	K562	none	ATF1	3.79E-04	1.40	0.49	4.60E-04	3.34	3.36
GSM1208761	LoVo	none	ISL1	4.24E-04	1.61	0.69	5.14E-04	3.29	3.35
GSE92026	HEK293T	none	TARDBP	4.53E-04	1.62	0.70	5.47E-04	3.26	3.32
wgEncodeEH002811	GM12878	none	IKZF1	4.38E-04	1.49	0.57	5.30E-04	3.28	3.31
GSM1306364	MGG8	none	SALL2	4.64E-04	1.56	0.64	5.60E-04	3.25	3.30
wgEncodeEH001835	H1-hESC	none	RFX5	4.66E-04	1.52	0.61	5.62E-04	3.25	3.29
GSM1899558	VCaP	none	ERG	4.75E-04	1.45	0.54	5.73E-04	3.24	3.27
wgEncodeEH003093	K562	none	HDAC6	5.87E-04	1.60	0.68	7.05E-04	3.15	3.21

GSM1876186	MCF-7	none	FOXM1	5.66E-04	1.36	0.44	6.81E-04	3.17	3.19
wgEncodeEH001780	HepG2	none	TCF7L2	6.30E-04	1.46	0.54	7.57E-04	3.12	3.15
wgEncodeEH001802	HepG2	none	NRF1	7.65E-04	1.80	0.84	9.16E-04	3.04	3.14
wgEncodeEH000622	GM12878	none	FOS	7.00E-04	1.39	0.47	8.40E-04	3.08	3.10
wgEncodeEH000748	K562	none	GTF3C2	7.18E-04	1.44	0.53	8.60E-04	3.07	3.10
GSM1239483	LoVo	none	PPARG	8.49E-04	1.65	0.72	1.02E-03	2.99	3.06
GSM2390642	HUVEC	Hypoxia	EPAS1	9.61E-04	1.82	0.87	1.15E-03	2.94	3.05
wgEncodeEH001562	GM12878	none	ATF3	8.68E-04	1.42	0.50	1.04E-03	2.98	3.01
GSM1239589	LoVo	none	FOXO4	9.41E-04	1.61	0.69	1.12E-03	2.95	3.01
wgEncodeEH001773	HEK293	none	ELK4	1.41E-03	2.13	1.09	1.67E-03	2.78	3.00
GSM1056835	macrophage	4h 1% O2	EPAS1	9.77E-04	1.52	0.61	1.16E-03	2.93	2.98
GSE91936	K562	none	TEAD2	1.06E-03	1.49	0.58	1.26E-03	2.90	2.94
wgEncodeEH001785	PBDEFetal	none	GATA1	1.15E-03	1.44	0.52	1.37E-03	2.86	2.90
GSE91969	IMR90	none	ELK1	1.30E-03	1.68	0.75	1.54E-03	2.81	2.89
GSM1208687	LoVo	none	TBX3	1.20E-03	1.32	0.40	1.42E-03	2.85	2.86
GSM957606	T-ALL	none	TCF3	1.24E-03	1.31	0.39	1.47E-03	2.83	2.85
GSM1405078	HeLa	none	AFF4	1.29E-03	1.39	0.47	1.52E-03	2.82	2.84
wgEncodeEH001788	HeLa-S3	none	IRF3	1.48E-03	1.49	0.58	1.75E-03	2.76	2.80
wgEncodeEH000393	GM12801	none	CTCF	1.45E-03	1.33	0.41	1.71E-03	2.77	2.79
GSM1065714	K562	30 min heatshock (42C)	HSF1	1.46E-03	1.31	0.39	1.73E-03	2.76	2.78
GSM1407651	HEK293	none	ZNF45	1.62E-03	1.39	0.48	1.90E-03	2.72	2.75
GSM2466501	HEK293T	none	ZNF224	4.81E-03	2.54	1.34	5.58E-03	2.25	2.73
GSM1407637	HEK293	none	ZNF41	1.88E-03	1.63	0.70	2.22E-03	2.65	2.73
GSE91473	HEK293T	none	FOXA1	2.57E-03	2.03	1.02	3.01E-03	2.52	2.72

wgEncodeEH002845	HepG2	none	RCOR1	4.08E-03	2.22	1.15	4.74E-03	2.32	2.62
GSM1525104	Jurkat	KDM3A phosphorylation	KDM3A	2.09E-03	1.26	0.34	2.46E-03	2.61	2.62
GSM2480800	LNCaP	none	AR	2.76E-03	1.75	0.81	3.22E-03	2.49	2.60
GSE97661.10	GM12878	none	TP73	2.50E-03	1.46	0.55	2.93E-03	2.53	2.57
wgEncodeEH000751	HepG2	forskolin	ESRRA	2.58E-03	1.44	0.52	3.02E-03	2.52	2.56
GSM700945	MCF-7	0.5%02 16h	EPAS1	2.61E-03	1.41	0.49	3.05E-03	2.52	2.55
wgEncodeEH000766	HeLa-S3	none	POLR3A	2.68E-03	1.46	0.54	3.13E-03	2.50	2.55
wgEncodeEH000695	GM12878	none	YY1	2.97E-03	1.56	0.64	3.46E-03	2.46	2.52
wgEncodeEH001862	HepG2	none	EP300	3.07E-03	1.47	0.55	3.58E-03	2.45	2.49
GSM1239508	LoVo	none	ID3	3.46E-03	1.46	0.55	4.03E-03	2.39	2.44
GSM2466461	HEK293T	none	ZKSCAN3	1.24E-02	2.58	1.37	1.40E-02	1.85	2.43
GSM1239431	LoVo	none	MAFB	5.79E-03	2.02	1.02	6.68E-03	2.18	2.40
wgEncodeEH002089	K562	none	EZH2	6.66E-03	2.13	1.09	7.66E-03	2.12	2.40
GSM1024799	MCF-7	none	AHR	4.18E-03	1.36	0.45	4.86E-03	2.31	2.34
GSM1065715	K562	30 min heatshock (42C)	HSF2	4.23E-03	1.31	0.39	4.91E-03	2.31	2.33
GSM1505752	HUES64	none	SMAD4	4.41E-03	1.37	0.45	5.12E-03	2.29	2.32
wgEncodeEH000701	K562	none	NELFE	5.91E-03	1.72	0.79	6.80E-03	2.17	2.29
GSM1239482	LoVo	none	PPARA	5.18E-03	1.52	0.61	5.99E-03	2.22	2.28
wgEncodeEH001527	H1-hESC	none	BCL11A	5.37E-03	1.39	0.47	6.21E-03	2.21	2.24
GSE94577.5	LTAD	DHT 1nM 24h	AR	5.33E-03	1.26	0.34	6.16E-03	2.21	2.23
GSM1239442	LoVo	none	NR2F6	9.25E-03	2.01	1.01	1.05E-02	1.98	2.22
GSM1208612	LoVo	none	ELF2	5.60E-03	1.33	0.41	6.46E-03	2.19	2.21

wgEncodeEH002086	K562	none	EP300	6.97E-03	1.65	0.73	7.97E-03	2.10	2.20
wgEncodeEH000752	HepG2	forskolin	NR3C1	7.04E-03	1.60	0.68	8.06E-03	2.09	2.18
wgEncodeEH001660	H1-hESC	none	FOSL1	6.54E-03	1.42	0.50	7.53E-03	2.12	2.16
wgEncodeEH000767	K562	none	BRF2	6.48E-02	2.82	1.50	7.02E-02	1.15	2.16
GSM1526874	HMEC-TERT	none	FOXM1	6.71E-03	1.33	0.42	7.71E-03	2.11	2.14
GSE98983.2	HepG2	TNF	RELA	7.69E-03	1.39	0.47	8.79E-03	2.06	2.09
wgEncodeEH000682	K562	none	NR2C2	9.85E-03	1.54	0.62	1.12E-02	1.95	2.02
wgEncodeEH000663	K562	IFNa30	STAT1	9.45E-03	1.42	0.51	1.08E-02	1.97	2.01
GSM1208616	LoVo	none	ETV4	8.81E-03	1.23	0.29	1.01E-02	2.00	2.01
GSM1693103	SK-N-SH	retinoid acid	RARA	1.06E-02	1.54	0.62	1.20E-02	1.92	1.99
GSE60006	SW1353	none	HES1	1.24E-02	1.59	0.67	1.40E-02	1.85	1.94
wgEncodeEH001560	H1-hESC	none	RXRA	1.11E-02	1.38	0.46	1.25E-02	1.90	1.94
GSM856791	786-O	none	ARNT	1.16E-02	1.31	0.39	1.31E-02	1.88	1.91
GSM810993	HeLa	none	E2F7	1.17E-02	1.32	0.40	1.32E-02	1.88	1.91
GSM2466491	HEK293T	none	ZNF197	1.50E-02	1.65	0.72	1.69E-02	1.77	1.89
wgEncodeEH000680	K562	none	POLR3A	1.31E-02	1.43	0.51	1.47E-02	1.83	1.88
GSM2466596	HEK293T	none	ZNF561	1.35E-02	1.48	0.56	1.52E-02	1.82	1.88
GSM1239497	LoVo	none	E2F2	2.10E-02	1.93	0.95	2.34E-02	1.63	1.88
GSM1505760	HUES64	none	SOX17	1.50E-02	1.36	0.44	1.69E-02	1.77	1.81
GSM1239389	LoVo	none	CREB3L1	2.51E-02	1.92	0.94	2.78E-02	1.56	1.81
GSM955978	HUVEC	24h 1% O2	HIF1A	1.63E-02	1.47	0.55	1.83E-02	1.74	1.80
GSM700944	MCF-7	0.5%02 16h	HIF1A	1.79E-02	1.54	0.62	2.00E-02	1.70	1.78
GSE76496.10	HEK293	none	ZNF419	1.57E-02	1.31	0.39	1.77E-02	1.75	1.78
GSM957607	T-ALL	none	TCF3	1.92E-02	1.57	0.65	2.15E-02	1.67	1.76
GSM991659	IMR90	none	E2F7	1.69E-02	1.24	0.30	1.90E-02	1.72	1.74

GSE91880	K562	none	YBX3	2.13E-02	1.60	0.68	2.37E-02	1.62	1.73
GSE91784	HEK293	none	ZNF140	1.80E-02	1.32	0.40	2.02E-02	1.70	1.72
wgEncodeEH001872	HeLa-S3	none	ZZZ3	5.86E-02	2.18	1.13	6.39E-02	1.19	1.68
GSE98983.1	HepG2	none	RELA	2.09E-02	1.37	0.45	2.33E-02	1.63	1.67
GSM1525103	Jurkat	none	KDM3A	2.01E-02	1.22	0.29	2.24E-02	1.65	1.66
GSM1312994	Caki-2	100 M desferoxamine	HIF1A	2.14E-02	1.28	0.36	2.38E-02	1.62	1.65
wgEncodeEH000757	HepG2	forskolin	PPARGC1A	2.70E-02	1.56	0.64	2.99E-02	1.52	1.62
GSM1208674	LoVo	none	SMAD2	2.35E-02	1.22	0.29	2.61E-02	1.58	1.60
wgEncodeEH002867	GM12878	none	E2F4	2.56E-02	1.31	0.38	2.84E-02	1.55	1.58
GSE92159	MCF-7	none	NONO	2.63E-02	1.32	0.40	2.91E-02	1.54	1.57
GSM1065722	K562	30 min heatshock (42C)	HSF2	2.84E-02	1.20	0.27	3.14E-02	1.50	1.52
GSM1693101	SK-N-SH	none	RARA	3.17E-02	1.39	0.48	3.50E-02	1.46	1.51
GSM1505693	HUES64	none	NR5A2	3.11E-02	1.26	0.33	3.44E-02	1.46	1.49
GSM2466599	HEK293T	none	ZNF565	3.82E-02	1.44	0.53	4.21E-02	1.38	1.45
GSM2466525	HEK293T	none	ZNF304	4.03E-02	1.47	0.56	4.43E-02	1.35	1.43
GSM959625	LS180	none	NCOA1	6.25E-02	1.82	0.87	6.80E-02	1.17	1.43
GSM1239558	LoVo	none	CREBL2	3.94E-02	1.36	0.44	4.33E-02	1.36	1.41
wgEncodeEH001764	K562	none	TRIM28	4.43E-02	1.29	0.36	4.86E-02	1.31	1.34
wgEncodeEH001552	PANC-1	none	REST	4.49E-02	1.21	0.28	4.93E-02	1.31	1.32
GSM1208694	LoVo	none	TFEB	4.74E-02	1.29	0.37	5.19E-02	1.28	1.32
GSE53041.9	THP-1	80 min calcitriol	VDR	5.02E-02	1.37	0.46	5.49E-02	1.26	1.31
GSM744581	primary epidermal keratinocytes	none	NFKBIA	4.85E-02	1.31	0.38	5.32E-02	1.27	1.31
GSE105589	HEK293	none	ZNF211	7.00E-02	1.66	0.73	7.57E-02	1.12	1.30

GSM1239564	LoVo	none	ELF4	6.30E-02	1.57	0.65	6.84E-02	1.17	1.30
GSM2466544	HEK293T	none	ZNF383	5.43E-02	1.41	0.49	5.92E-02	1.23	1.29
GSM2466522	HEK293T	none	ZNF2	5.00E-02	1.21	0.27	5.47E-02	1.26	1.28
GSM2466588	HEK293T	none	ZNF548	7.35E-02	1.63	0.70	7.93E-02	1.10	1.27
GSM1589798	CXCR5hi CD4 T cells	none	BCL6	6.15E-02	1.46	0.55	6.69E-02	1.17	1.26
wgEncodeEH001490	A549	DEX_500pM	NR3C1	8.79E-02	1.68	0.75	9.45E-02	1.02	1.23
GSM1407645	HEK293	none	ZBTB18	6.41E-02	1.35	0.43	6.95E-02	1.16	1.21
GSM2466495	HEK293T	none	ZNF211	1.13E-01	1.76	0.81	1.22E-01	0.92	1.19
wgEncodeEH002264	HepG2	none	SP2	6.27E-02	1.21	0.28	6.82E-02	1.17	1.19
wgEncodeEH002068	K562	none	ZNF274	6.46E-02	1.26	0.33	7.00E-02	1.15	1.18
GSE91769	MCF7	none	ZKSCAN1	6.59E-02	1.22	0.28	7.13E-02	1.15	1.17
wgEncodeEH000697	GM12878	none	NR2C2	9.39E-02	1.42	0.51	1.01E-01	1.00	1.08
GSM1407653	HEK293	none	ZNF33A	8.46E-02	1.24	0.31	9.11E-02	1.04	1.07
GSE105684	HepG2	none	NONO	1.22E-01	1.58	0.66	1.30E-01	0.89	1.06
wgEncodeEH000698	GM12878	none	ZZZ3	1.25E-01	1.56	0.64	1.33E-01	0.88	1.04
GSM1239491	LoVo	none	BACH1	1.05E-01	1.31	0.39	1.13E-01	0.95	1.00
wgEncodeEH002299	T-47D	BPA_100nM	ESR1	1.26E-01	1.36	0.44	1.35E-01	0.87	0.94
GSM1208648	LoVo	none	MEF2A	2.09E-01	1.66	0.73	2.22E-01	0.65	0.93
GSM2466621	HEK293T	none	ZNF641	1.61E-01	1.52	0.61	1.71E-01	0.77	0.93
GSM2466601	HEK293T	none	ZNF567	1.79E-01	1.54	0.63	1.90E-01	0.72	0.90
GSM1898102	SH-SY5Y	none	SATB1	1.72E-01	1.33	0.41	1.83E-01	0.74	0.81
wgEncodeEH000747	HeLa-S3	none	GTF3C2	1.62E-01	1.26	0.33	1.72E-01	0.76	0.81
GSM1065721	K562	30 min heatshock (42C)	HSF1	1.57E-01	1.18	0.24	1.68E-01	0.78	0.80
GSM1239556	LoVo	none	CDC5L	1.90E-01	1.38	0.47	2.01E-01	0.70	0.80

GSE91793	A549	none	ESRRA	1.56E-01	1.16	0.22	1.67E-01	0.78	0.79
GSM1239416	LoVo	none	HOXD9	2.32E-01	1.45	0.53	2.45E-01	0.61	0.76
GSM1208677	LoVo	none	SMAD4	1.91E-01	1.17	0.23	2.02E-01	0.69	0.71
GSM1239583	LoVo	none	MYNN	2.68E-01	1.34	0.43	2.82E-01	0.55	0.65
GSM1239466	LoVo	none	IRF2	2.65E-01	1.28	0.36	2.80E-01	0.55	0.62
wgEncodeEH000769	K562	MNaseD	SETDB1	2.47E-01	1.20	0.26	2.60E-01	0.58	0.62
GSM1239481	LoVo	none	NR4A1	2.76E-01	1.29	0.36	2.90E-01	0.54	0.61
wgEncodeEH002313	ECC-1	BPA_100nM	ESR1	2.67E-01	1.26	0.33	2.81E-01	0.55	0.61
GSE38567.1	PBMCs	none	STAT4	3.33E-01	1.30	0.38	3.49E-01	0.46	0.55
GSM1024800	MCF-7	none	ARNT	3.17E-01	1.16	0.21	3.33E-01	0.48	0.50
GSM1208698	LoVo	none	XBP1	3.19E-01	1.11	0.14	3.35E-01	0.48	0.49
GSM1239549	LoVo	none	ARNTL2	3.92E-01	1.24	0.31	4.09E-01	0.39	0.46
GSE53041.10	THP-1	none	VDR	3.68E-01	1.17	0.23	3.85E-01	0.41	0.45
GSE94577.4	LNCaP	DHT 1nM 24h	AR	3.62E-01	1.10	0.14	3.79E-01	0.42	0.43
GSM959626	LS180	3h calcitriol	NCOA1	4.00E-01	1.20	0.27	4.16E-01	0.38	0.43
GSE91981	HEK293	none	ZNF792	3.77E-01	1.13	0.18	3.94E-01	0.40	0.43
wgEncodeEH001808	GM12878	none	NFE2	4.25E-01	1.21	0.27	4.42E-01	0.35	0.41
GSE106063	HEK293	none	ZNF548	4.10E-01	1.13	0.18	4.26E-01	0.37	0.39
GSE91444	MCF-7	none	HSF1	4.10E-01	1.13	0.18	4.26E-01	0.37	0.39
GSE91630	MCF-7	none	SIX4	4.68E-01	1.14	0.19	4.85E-01	0.31	0.34
GSM1065712	K562	none	HSF2	5.04E-01	1.16	0.22	5.20E-01	0.28	0.33
wgEncodeEH003080	Dnd41	none	EZH2	4.74E-01	1.10	0.14	4.91E-01	0.31	0.33
GSM1239537	LoVo	none	PBX2	5.28E-01	1.15	0.20	5.45E-01	0.26	0.30
GSM1239398	LoVo	none	FOXO1	6.10E-01	1.22	0.29	6.27E-01	0.20	0.30
GSM1410765	LNCaP	none	CTBP2	5.95E-01	1.19	0.25	6.12E-01	0.21	0.28
GSM1239562	LoVo	none	DRAP1	5.87E-01	1.12	0.17	6.05E-01	0.22	0.25
GSM2466455	HEK293T	none	ZIK1	6.71E-01	1.19	0.25	6.86E-01	0.16	0.25
GSM1239581	LoVo	none	MEOX2	6.52E-01	1.10	0.13	6.68E-01	0.18	0.20

GSM1239463	LoVo	none	HOXB4	6.65E-01	1.11	0.15	6.80E-01	0.17	0.20
GSE101045	H1-hESC	none	KDM5B	7.02E-01	1.11	0.15	7.15E-01	0.15	0.18
wgEncodeEH000678	K562	none	BDP1	7.21E-01	1.07	0.10	7.32E-01	0.14	0.15
GSM1239438	LoVo	none	NFKB1	8.08E-01	1.10	0.13	8.16E-01	0.09	0.13
wgEncodeEH002268	PFSK-1	none	SIN3A	7.65E-01	1.05	0.06	7.74E-01	0.11	0.12
GSM1239383	LoVo	none	ARNTL	7.69E-01	1.02	0.04	7.78E-01	0.11	0.11
wgEncodeEH001811	GM12878	none	STAT3	8.30E-01	1.04	0.05	8.37E-01	0.08	0.09
GSM1876187	MCF-7	none	FOXM1	8.44E-01	1.04	0.06	8.51E-01	0.07	0.08
GSM1239433	LoVo	none	MIXL1	8.89E-01	1.03	0.04	8.94E-01	0.05	0.06
GSM1239560	LoVo	none	DDIT3	8.89E-01	1.02	0.03	8.94E-01	0.05	0.05
wgEncodeEH001800	HepG2	none	IRF3	1.00E+00	1.04	0.06	1.00E+00	0.00	0.04
GSM1208696	LoVo	none	TP53	1.00E+00	1.00	-0.01	1.00E+00	0.00	0.00
wgEncodeEH000763	HeLa-S3	none	BDP1	1.00E+00	0.98	-0.03	1.00E+00	0.00	-0.02
GSM1239621	LoVo	none	ZBTB4	8.95E-01	0.91	-0.14	8.99E-01	0.05	-0.11
GSE101211	GM12878	none	CBX3	1.00E+00	0.90	-0.15	1.00E+00	0.00	-0.11
wgEncodeEH000652	NT2-D1	none	SUZ12	7.39E-01	0.95	-0.08	7.49E-01	0.13	-0.14
GSE105081	hESC	BMP4 3 days	TFAP2A	8.83E-01	0.88	-0.18	8.90E-01	0.05	-0.14
wgEncodeEH003084	HUVEC	none	EZH2	7.15E-01	0.96	-0.06	7.28E-01	0.14	-0.15
wgEncodeEH000596	H1-hESC	none	MYC	7.31E-01	0.92	-0.12	7.41E-01	0.13	-0.16
GSM1239485	LoVo	none	RELB	7.47E-01	0.91	-0.14	7.57E-01	0.12	-0.16
GSM1407646	HEK293	none	ZSCAN31	6.72E-01	0.92	-0.12	6.87E-01	0.16	-0.19
GSM2466569	HEK293T	none	ZNF480	7.16E-01	0.87	-0.20	7.28E-01	0.14	-0.20
wgEncodeEH003082	H1-hESC	none	EZH2	5.22E-01	0.92	-0.12	5.39E-01	0.27	-0.28
GSE101135	H1-hESC	none	CBX3	6.14E-01	0.83	-0.28	6.29E-01	0.20	-0.29

wgEncodeEH002866	GM08714	none	ZNF274	6.29E-01	0.77	-0.38	6.45E-01	0.19	-0.36
GSM955977	HUVEC	none	HIF1A	6.90E-01	0.75	-0.41	7.04E-01	0.15	-0.37
GSM1239443	LoVo	none	NR3C2	6.92E-01	0.72	-0.47	7.06E-01	0.15	-0.41
GSE53041.6	LS180	none	VDR	8.01E-01	0.71	-0.50	8.10E-01	0.09	-0.42
wgEncodeEH002416	HepG2	none	EZH2	3.84E-01	0.86	-0.21	4.00E-01	0.40	-0.43
wgEncodeEH000645	GM12878	none	POLR3G	4.94E-01	0.76	-0.39	5.11E-01	0.29	-0.43
wgEncodeEH000694	K562	none	POLR3G	4.76E-01	0.76	-0.40	4.93E-01	0.31	-0.45
wgEncodeEH003086	HeLa-S3	none	EZH2	4.33E-01	0.76	-0.40	4.49E-01	0.35	-0.47
wgEncodeEH000696	K562	none	ZNF274	5.32E-01	0.65	-0.62	5.48E-01	0.26	-0.60
wgEncodeEH001756	GM12878	none	ZNF274	1.00E+00	0.61	-0.71	1.00E+00	0.00	-0.63
GSM2466662	HEK293T	none	ZNF789	3.41E-01	0.65	-0.61	3.57E-01	0.45	-0.69
wgEncodeEH002412	HMEC	none	EZH2	1.59E-01	0.83	-0.26	1.69E-01	0.77	-0.80
wgEncodeEH001763	HeLa-S3	none	ZNF274	5.88E-01	0.55	-0.87	6.05E-01	0.22	-0.85
GSE28337	MCF7	none	KDM5B	2.22E-01	0.61	-0.72	2.34E-01	0.63	-0.90
wgEncodeEH000679	K562	none	BRF1	1.58E-01	0.58	-0.78	1.69E-01	0.77	-1.06
wgEncodeEH002418	NHEK	none	EZH2	7.19E-02	0.83	-0.27	7.76E-02	1.11	-1.13
wgEncodeEH002438	NHDF-Ad	none	EZH2	5.12E-02	0.80	-0.32	5.59E-02	1.25	-1.28
wgEncodeEH003083	HSMMtube	none	EZH2	9.79E-03	0.68	-0.56	1.11E-02	1.95	-2.01
wgEncodeEH002425	NH-A	none	EZH2	8.85E-03	0.75	-0.41	1.01E-02	2.00	-2.02
wgEncodeEH002422	NHLF	none	EZH2	6.95E-03	0.74	-0.43	7.96E-03	2.10	-2.13
wgEncodeEH001752	H1-hESC	none	SUZ12	6.87E-03	0.63	-0.67	7.89E-03	2.10	-2.18

wgEncodeEH001775	NT2-D1	none	ZNF274	2.15E-02	0.33	-1.58	2.39E-02	1.62	-2.56
wgEncodeEH001755	HepG2	none	ZNF274	9.55E-02	0.20	-2.31	1.03E-01	0.99	-4.08
