# Supplementary material for: Nutritional restriction during the peri-conceptional period alters the myometrial transcriptome during the peri-implantation period
Source: Sci Rep. 2021 Oct 27;11:21187. doi: 10.1038/s41598-021-00533-x (PMC8551329; doi:10.1038/s41598-021-00533-x)
Supplement: Supplementary file 9 — Supplementary Table 5. [file 41598_2021_533_MOESM9_ESM.pdf]

Nutritional restriction during the peri-conceptional period alters the myometrial transcriptome during the peri-implantation period

Ewa Monika Drzewiecka, Wiktoria Kozlowska, Agata Zmijewska, Anita Franczak*

Affiliation: Department of Animal Anatomy and Physiology, University of Warmia and Mazury in Olsztyn, Oczapowskiego 1A, 10-719 Olsztyn, Poland

*Corresponding Author: Anita Franczak, Department of Anatomy and Animal Physiology, Faculty of Biology and Biotechnology, University of Warmia and Mazury
in Olsztyn, Oczapowskiego 1A, 10-719 Olsztyn, Poland; e-mail: anitaf@uwm.edu.pl

Supplementary table 5. Common differentially expressed genes (DEGs) in the myometrium, endometrium, and embryos collected from sows during the peri-implantation period that were fed a restrictive diet during the peri-conceptional period compared to sows fed a normal diet.

Differentially expressed genes in restricted diet-fed pigs comparing to normal-diet-fed sows			
**Common DEGs for myometrium, endometrium and conceptuses**	**Common DEGs for myometrium and endometrium**	**Common DEGs for myometrium and conceptuses**	**Common DEGs for endometrium and conceptuses**
**Total count: 19**	**Total count: 69**	**Total count: 113**	**Total count: 76**
FTH1	ABCB7	VPS33A	TRA2B
LUM	SLC25A3	ATP5F1	GSTA2
C1S	CYP39A1	RGN	APOE
ND2	CYCS	PIKFYVE	GMPR
ACP5	RDH10	SNX16	PPP2R5A
OAS2	SOD2	SMC3	PAQR8
MX1	KPNA2	NPC2	LDLR
DHX58	BIRC5	FPGT	ANKRD22
TSPAN3	GHR	FRA10AC1	TPD52L1
PRDX6	COPS4	DYNC1H1	MYC
GSTA4	YWHAH	SLC35A5	TIMP1
KIFC1	TLK1	IFN-DELTA-6	CBX2
MYL6	PGM1	KLF4	POT1
MCL1	SNRPC	SH3D19	SPAG5
M6PR	CYB561	VDAC1P5	CA4
RHOB	EIF2S1	RPN1	MAPKAPK3
COL14A1	BAG3	UABP-2	CFL2
MICAL3	CDS2	LCMT2	TUSC3
GPX3	S100A16	CD86	THBS1
	MTF2	WWP1	CHEK2
	GLO1	PTTG1	CA3
	CD302	DGCR8	PKIA
	GHITM	COPS6	ZFAND5
	DNPEP	RBM4B	KDM1B
	PDE4B	PPP3CB	TOR4A
	MSMO1	SLA-5	ACP6
	CD164	ZDHHC7	FUT2
	ACTA1	NTRK2	SMAP2
	SDF2L1	GNAZ	ACSL4
	CYR61	DAZAP2	HOPX
	EEA1	ZFP36L1	CHSY1
	CKAP4	LGALS3	LOC100515931
	FZR1	BAZ2B	PN-1
	RAE1	LRP8	FAS
	PLAU	GBP1	C-ERBA-B1
	GJA1	LOC100152229	LOC100154269
	SLC39A14	RNF111	CFH
	RPP40	REPS1	JAG1
	PTPN11	SLC29A1	MTTP
	ACOX1	ST3GAL4	KLRK1
	CD40	C-FLIP	LOC100513756
	CS	ALG5	SULT1A1
	ZNF318	TTC14	ITGAV
	ITGA1	MUC13	COX1
	HOXA3	CYB5A	GTF2H4
	ANAPC7	ME1	ACSL5
	BZW1	MCM8	SLC35B3
	CLDND1	COL1A2	PRNP
	KIF21A	SLC16A1	HMGB2
	ATG5	ABLIM1	LIMK2
	SLC4A7	OAT	GPX6
	IFRD1	NMB	DDIT4
	OGT	SLC25A37	PPEF1

	CD59	FBXL6	DNMT1
	SPTLC2	ZBTB22	TSPO
	HSPA5	PGRMC2	MYCBP2
	SRPRB	SLCO3A1	TPK1
	BICC1	CHD6	GNB2L1
	RIT1	DYNC1LI2	LGALS9
	AKAP13	RHOA	DLX5
	ANGPTL2	PPIG	ETS1
	WSB2	ATP2C1	FLAD1
	HNRNPA3	TUBA1B	AAGAB
	YIPF5	HUWE1	S100A10
	HSD11B1	FABP3	PPAT
	SEPHS2	VLDLR	NCALD
	BTG3	LIPA	SLC34A2
	CCDC88A	TPM1	COL6A3
	RXRB	ISG20L2	CLDN8
		SMEK1	LOC100519683
		CD9	RAB27A
		TYSND1	MT-III
		NHEJ1	TIMP2
		SGMS1	GPRC5B
		DRG1	ACOX2
		UROS	CYP24A1
		PEX6	
		ATF4	
		TXNIP	
		RAN	
		ISG15	
		RTN4	
		ABCG2	
		CMAH	
		WDR11	
		PSAP	
		RBP4	
		CDO1	
		LAMB1	
		TRIM21	
		NFIB	
		IGFBP1	
		INTS9	
		IFI30	
		ANAPC16	
		CPNE3	
		SFRS18	
		IFNGR1	
		COX2	
		INSR	
		NFX1	
		JAK1	
		ITGB3	
		LOC100737714	
		PXK	
		BAX	
		FXYD3	
		SMEK2	
		SAR1B	
		ANXA11	
		PRKAG1	
		RPS6KA3	
		KBTBD7	
