# Supplementary material for: Nutritional restriction during the peri-conceptional period alters the myometrial transcriptome during the peri-implantation period
Source: Sci Rep. 2021 Oct 27;11:21187. doi: 10.1038/s41598-021-00533-x (PMC8551329; doi:10.1038/s41598-021-00533-x)
Supplement: Supplementary file 10 — Supplementary Table 6. [file 41598_2021_533_MOESM10_ESM.pdf]

Nutritional restriction during the peri-conceptional period alters the myometrial transcriptome during the peri-implantation period

Ewa Monika Drzewiecka, Wiktoria Kozlowska, Agata Zmijewska, Anita Franczak*

Affiliation: Department of Animal Anatomy and Physiology, University of Warmia and Mazury in Olsztyn, Oczapowskiego 1A, 10-719 Olsztyn, Poland

*Corresponding Author: Anita Franczak, Department of Anatomy and Animal Physiology, Faculty of Biology and Biotechnology, University of Warmia and Mazury in Olsztyn, Oczapowski 1A, 10-719 Olsztyn, Poland;
e-mail: anitaf@uwm.edu.pl

Supplementary table 6. A list of differentially expressed genes in the myometrium of pigs during the peri-implantation period that were fed a restrictive diet during the peri-conceptional period comparing to the myometrium of pigs during the peri-implantation period that were fed a normal diet during the peri-conceptional period, that were selected for validation of microarray results, with the indication of the sequences of primers used for this analysis.

Official gene symbol	Gene name	Primers sequence (5’→ 3’)	Accession No.	Annealing (ºC)
		Up-regulated genes		
PGRMC1	*Progesterone receptor membrane component 1*	F: TACGGGGTCTTTGCTG<br>R: ACGTGATGGTACTTGAAAGTG	NM_213911	56.5
SRC	*SRC proto-oncogene, non-receptor tyrosine kinase*	F: GGTCTATCCCAGGTCACCG<br>R: CCTCTGAGCCTCTGTTCTGG	XM_021077969	52
HSD17B8	*Hydroxysteroid (17β) dehydrogenase 8*	F: TTCTGCTCCGCATGTCTGAAG<br>R: CCATGTTTCCCACCTTCCCTA	NM_001130730	59
PCNA	*Proliferating cell nuclear antigen*	F: GCAGACACCTTGGCACTA<br>R: ACAGCTGTACTCTTGTTCTGGA	NM_001291925	59
ANXA2	*Annexin A2*	F: TCTGCATTTGGGGACG<br>R: CGCTGATGCAAGTTCCTT	NM_001005726	57.5
		Down-regulated genes		
SCAPER	*S-phase cyclin A-associated protein in the ER*	F: GGCTGACAAGGTAAAGGCT<br>R: GCAAGAACTTCCGCCATAG	XM_021099012	52
PTGFR	*Prostaglandin F receptor*	F: CTTTCCCCTAAGTGGTCGGG<br>R: TCCAGAGATCTTGGCATCCCC	XM_005665359	60
HOXA10	*Homeobox A10*	F: CTCACGGACAGACAAGTTAAA<br>R:ATGACAGAGGGAGGAGAAC	NM_001257354.1	60
COX2	*Cyclooxygenase 2*	F: ATGATCTACCCGCCTCACAC<br>R: AAAAGCAGCTCTGGGTCAAA	AF207824.1	57
PGRMC2	*Progesterone receptor membrane component 2*	F: AAACCAGGGGAAGAACCGTC<br>R: ACCCAAAGACGCTGGACATT	NM_001097521.2	60
		Reference genes		
ACTB	*Actin beta*	F: GGAGATCGTGCGGGACATCAAG<br>R: GGCGTAGAGGTCCTTCCTGATG	U07786.1	60
RNA 18S	*RNA, 18S ribosomal*	F: GGCTACCACATCCAAGGAAG<br>R: TCCAATGGATCCTCGCGGAA	NR_046261.1	60
